# Syntheses and Structure–Activity Relationships in Growth Inhibition Activity against Human Cancer Cell Lines of 12 Substituted Berberine Derivatives

**DOI:** 10.3390/molecules25081871

**Published:** 2020-04-18

**Authors:** Bo Wang, An-Jun Deng, Zhi-Hong Li, Nan Wang, Hai-Lin Qin

**Affiliations:** State Key Laboratory of Bioactive Substance and Function of Natural Medicines, Institute of Materia Medica, Chinese Academy of Medical Sciences and Peking Union Medical College, Beijing 100050, China; wangbping@yeah.net (B.W.); zhl@imm.ac.cn (Z.-H.L.); wangnan@imm.ac.cn (N.W.)

**Keywords:** quaternary berberine chloride, structure modification, berberine-12-amine derivatives, growth inhibition activities against cancer cell lines, structure–activity relationships

## Abstract

In this study, quaternary berberine chloride is used as a lead compound to design and synthesize a series of berberine-12-amine derivatives to evaluate the growth inhibition activity against human cancer cell lines. Forty-two compounds of several series were obtained. The quaternary berberine-12-*N,N*-di-*n*-alkylamine chlorides showed the targeted activities with the IC_50_ values of most active compounds being dozens of times those of the positive control. A significant structure–activity relationship (SAR) was observed. The activities of quaternary berberine-12-*N,N*-di-*n*-alkylamine chlorides are significantly stronger than those of the reduced counterparts. In the range of about 6-8 carbon atoms, the activities increase with the elongation of *n*-alkyl carbon chain of 12-*N,N*-di-*n*-alkylamino, and when the carbon atom numbers are more than 6-8, the activities decrease with the elongation of *n*-alkyl carbon chain. The activities of the tertiary amine structure are significantly higher than that of the secondary amine structure.

## 1. Introduction

Nearly all kinds of malignant tumors (cancers) are severe diseases endangering human health. The search for effective therapies against malignancies has gone through a long historical period. Currently, surgical therapy, radiotherapy, chemotherapy (including molecular-targeted drug therapy), endocrine therapy, and biotherapy are the most commonly used therapies to treat malignancies in the clinic. However, it is very worrying that while a series of the most commonly diagnosed malignant tumors in the clinic represent one of the leading causes of diseases-related deaths worldwide, the clinical benefits of these therapies are all limited and there are no specific drugs or other therapies available for the treatment of many cancers. Poor curative effect and obvious toxicity or hypertoxicity are the outstanding defects of current anticancer drugs [1]. Thus, better treatment options against malignancies, including innovative antitumor drugs, are urgently needed.

**Figure 1:**
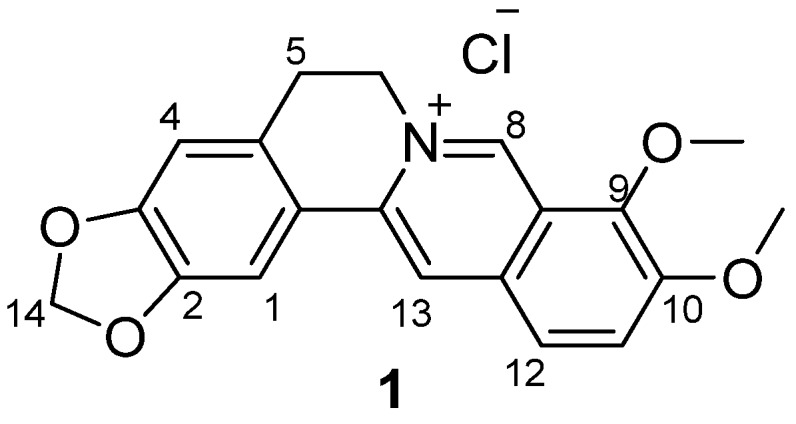
Structure of quaternary berberine chloride (QBC) (**1**).

Berberine-type alkaloids are a class of natural isoquinoline alkaloids. Quaternary berberine chloride (QBC, **1**, Figure 1) is an outstanding representative of berberine-type alkaloids which exists in many well-known medicinal plants, such as *Coptis chinensis* Franch. of Ranunculaceae [2,3] and many *Berberis* species of Berberidaceae [4]. QBC is rich in natural resources and now has been synthesized on a large scale in chemical industry [2,5]. QBC has a wide range of pharmacological activities such as anti-tumor [6,7], antidiarrheal [8], hypoglycemic [9], antibacterial [10,11], hypolipidemic [12], antihypertensive [13], antiarrhythmic [14], neuroprotective [15], and anti-inflammatory [16] activities. In terms of anti-tumor activity, the growth inhibitory effect of QBC against cancer cells was one of the major hot spots that were concerned. However, the problems of poor pharmacological effect and poor pharmacokinetic characteristics of QBC itself further limit its clinical application [17].

Research on berberine-type alkaloids is one of the hot topics in the field of medicinal chemistry at present, particularly research on structural modification and many other pharmacological activities. Our group has also been performing these investigations over the past few years, and has touched on many aspects of several berberine-type alkaloids in pharmaceutical chemistry, such as studies on the structural modifications of quaternary coptisine chloride and quaternary palmatine chloride, the exploration of the pharmacological activities of X-box-binding protein 1 (XBP1), which include transcriptional activation, anti-ulcerative colitis, and antibacterial [18,19,20,21,22]. Recently, we ran a study on the structural modification of QBC to explore the possibility of improving its activity for inhibiting human cancer cell growth, and to investigate the structure–activity relationship (SAR). In our study, the covalently connected hydrogen atom at position 12 of the QBC core was replaced by *N*-acylamino-, *N,N*-di-*n*-alkylamino-, and *N*-*n*-alkylamino-, respectively, and the different reduction states of the end-products were also studied in order to yield several classes of new berberine-type alkaloid derivatives as target compounds. All the synthesized compounds were screened for the in vitro inhibition of human cancer cell growth. Two classes of end-products exhibited definite activities and a structure–activity relationship, with one class of compounds showing the activity more than dozens of times that of fluorouracil (5-FU), which was used as a positive control. This article reports on the design and syntheses of the target compounds, the evaluation of the in vitro inhibition of cancer cell growth, and the SAR analysis.

## 2. Results and Discussion

### 2.1. Chemistry

By consulting published literature related to QBC, it was found that, in addition to the replacement of hydrogen atom at position-12 of the QBC core by halogens, there has been no research reports on the structural modification based on position-12. However, as far as organic chemistry is concerned, it is obvious that the electrophilic nitration reaction at position-12 is possibly more likely to occur due to the influence of the methoxy groups in positions-9 and -10. Following nitration, the reduction of the nitro group leads to the formation of a primary amino group, which can be capitalized on to carry out multiple other structural modifications.

Thus, in the current study, the QBC was firstly nitrated under the conditions of NaNO_2_ plus concentrated HNO_3_ to produce quaternary 12-nitroberberine chloride (**2**) (Figures S1 and S2, Supplementary Materials). Compound **2** was reduced under the condition of SnCl_2_•2H_2_O to yield quaternary 12-aminoberberine chloride (**3**) (Figures S3 and S4). Then, compound **3** was reacted with relevant acyl chlorides to successfully synthesize the targeted quaternary berberine-12-*N*-acylamine chloride derivatives (**4a**–**m**) (Scheme 1). On top of the signature signals of H-1 (s, 1H), 4 (s, 1H), 8 (s, 1H), 11 (s, 1H), 13 (s, 1H), MeO-9 (s, 3H), MeO-10 (s, 3H), CH_2_-5 (t, 2H), CH_2_-6 (t, 2H), and CH_2_-14 (s, 2H) of the QBC core, all the synthesized target compounds showed signals that corresponded to acyls in the ^1^H NMR spectra. All the positive ESIMS data was also consistent with the structures (see Materials and Methods section and Figures S5–S29).

To synthesize the targeted quaternary berberine-12-*N,N*-di-*n*-alkylamine chlorides, compound **2** was reduced using NaBH_4_ and NiCl_2_·6H_2_O as reagents to yield the tertiary 12-aminotetrahydroberberine (**5**). Compound **5** was reacted with relevant aliphatic aldehydes through a process of addition and reduction reactions to obtain tetrahydroberberine-12-*N,N*-di-*n*-alkylamine derivatives (**6a**–**l**). Compounds **6a**–**l** were oxidized using 2,3-dichloro-5,6-dicyano-1,4-benzoquinone (DDQ) as the reagent and then reacted with 2N hydrochloric acid (Scheme 2) to yield the targeted quaternary berberine-12-*N,N*-di-*n*-alkylamine chlorides (**7a**–**l**) (Scheme 2) [23]. All the structures of the synthesized compounds were confirmed by NMR and MS methods (see Materials and Methods section and Figures S5–S79).

**Scheme 3:**
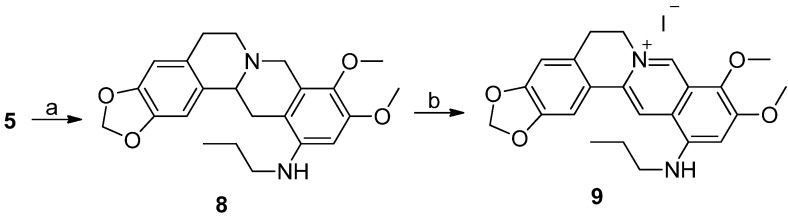
Syntheses of compounds **8** and **9**. Reagents and conditions: (**a**) RCHO, NaBH(OCOCH_3_)_3_, AcOH, CH_2_Cl_2_, rt, 1h; (**b**) I_2_, absolute EtOH, reflux, 24h.

In addition, in order to explore the effect of 12 *N*-monosubstituted amine counterparts of the synthesized compounds **6a**–**l** and **7a**–**l** on the inhibition of human cancer cell growth, tertiary tetrahydroberberine-12-*N*-*n*-propylamine derivatives (**8**) and quaternary berberine-12-*N*-*n*-propylamine chloride (**9**) as representatives were also designed and synthesized by capitalizing on the steric hindrance at position-12 and, in particular, carefully controlling for the amount of aldehyde. The synthesis is indicated in Scheme 3. The tertiary 12-aminotetrahydroberberine (**5**) was reacted with a carefully controlled amount of propionaldehyde to obtain **8** through the same process of addition and reduction reactions as synthesizing the tetrahydroberberine-12-*N,N*-di-*n*-alkylamine derivatives. Then, the target compound, quaternary berberine-12-*N*-*n*-propylamine iodide (**9**), was synthesized by oxidization under the condition of iodine. All the structures of the synthesized compounds were confirmed by NMR and MS methods (see Materials and Methods section and Figures S80 and S81).

### 2.2. Biological Activities

All the synthesized compounds were evaluated for the growth inhibition activity against several human cancer cell lines using the 3-(4,5-dimethylthiazol-2-yl)-2,5-diphenyltetrazolium bromide (MTT) assay that was modeled after our previous publication [24,25]. The cancer cells in this study included human colorectal cancer cells (HCT-8), human liver cancer cells (Bel7402), human cervical cancer cells (HeLa), human lung cancer cells (A549), and human gastric cancer cells (BGC-823). A clinically applied strong thymidylate synthetase inhibitor, 5-FU, was used as the positive control.

Experimental results showed that the intermediates quaternary 12-nitroberberine chloride (**2**), quaternary 12-aminoberberine chloride (**3**), tertiary 12-aminotetrahydroberberine (**5**), and tertiary tetrahydroberberine-12-*N*-*n*-propylamine (**8**), and the target compounds quaternary berberine-12-*N*-acylamine chloride derivatives (**4a**–**m**) and quaternary berberine-12-*N*-*n*-propylamine iodide (**9**) showed little or very little growth inhibition activity against the tested human cancer cell lines, each showing IC_50_ values greater than 10 µM (see Table 1, but data of **4a**–**m** not shown). Most of the tertiary tetrahydroberberine-12-*N,N*-di-*n*-alkylamine derivatives (**6a**–**l**) also showed little or very little growth inhibition activity against the tested human cancer cell lines, but a general SAR is obvious for this series of compounds. In the range of four carbon atoms for the *n*-alkyl carbon chain from 12-*N,N*-di-*n*-alkylaminos of series compounds **6a**–**l**, the title activity increases with the elongation of the *n*-alkyl carbon chain, and when there are more than four carbon atoms, the activity decreases with the elongation of the *n*-alkyl carbon chain. Tetrahydroberberine-12-*N,N*-di-*n*-butylamine (**6d**), as the most active compound in the **6a**–**l** series, only showed IC_50_ values inhibiting the growth of HCT-8, Bel7402, HeLa, A549, and BGC-823 by 12.81 μM, 16.38 μM, 9.74 μM, 11.40 μM, and 10.43 μM, respectively.

**Figure 2:**
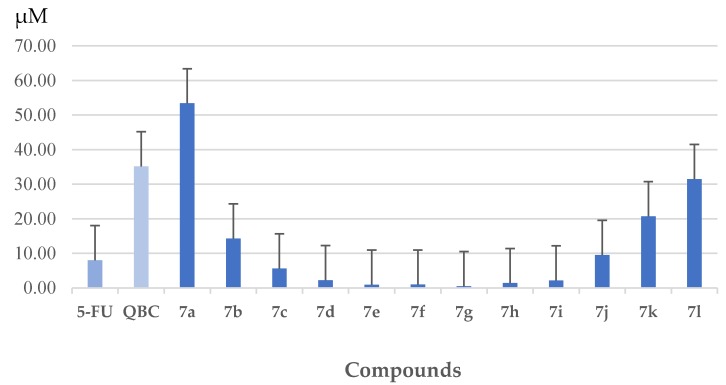
Effects of the active compounds inhibiting HCT-8 growth (*n* = 3).

**Figure 3:**
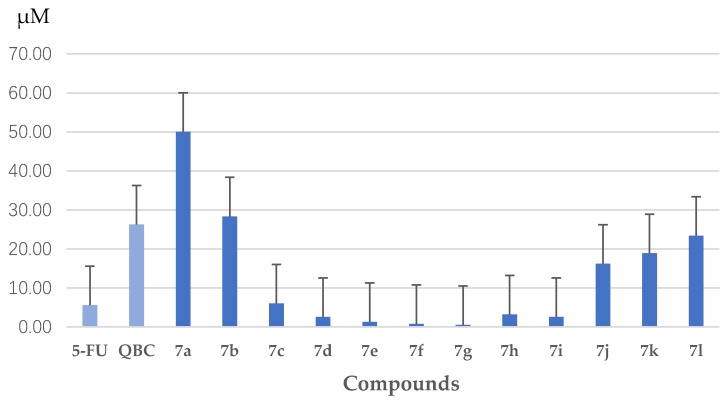
Effects of the active compounds inhibiting Bel7402 growth (*n* = 3).

**Figure 4:**
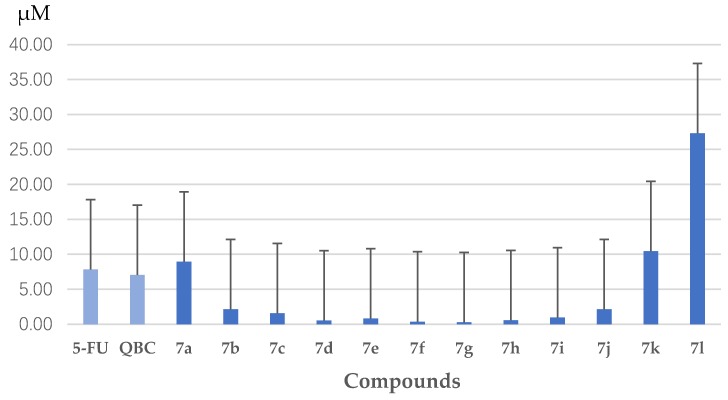
Effects of the active compounds inhibiting Hela growth (*n* = 3).

**Figure 5:**
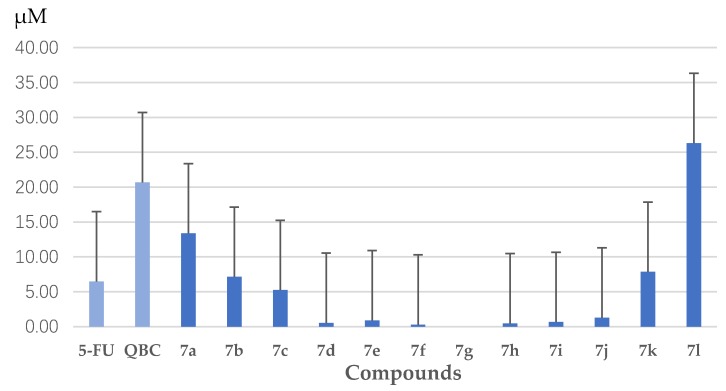
Effects of the active compounds inhibiting A549 growth (*n* = 3).

**Figure 6:**
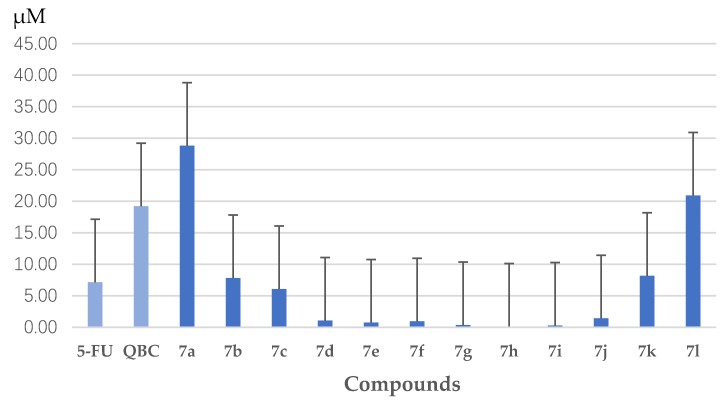
Effects of the active compounds inhibiting BGC-823 growth (*n* = 3).

However, the targeted quaternary berberine-12-*N,N*-di-*n*-alkylamine chloride series compounds **7a**–**l** exhibited, to varying degrees, some or significant growth inhibition activities against the tested human cancer cell lines. By comparison, **7a**–**l** are much more active than their reduction state counterparts **6a**–**l**, with the IC_50_ values of most compounds in the quaternary berberine-12-*N,N*-di-*n*-alkylamine chloride series being on or over the micromolar level (Table 1). This result demonstrated the impact of variations in the reduction states of the end-products on the title bioactivity. Still, the carbon chain length of the *n*-alkyl substituents was found to be an important factor to affect the activity of the end-products for all the tested human cancer cell lines. Quaternary berberine-12-*N,N*-dimethylamine chloride (**7a**), which was the nascent compound of the active quaternary berberine-12-*N,N*-di-*n*-alkylamine chloride series, showed growth inhibition activities against HCT-8, Bel7402, Hela, A549, and BGC-823 with IC_50_ values of 53.41 µM, 50.01 µM, 8.95 µM, 13.37 µM, and 28.82 µM, respectively. Then, an obvious trend was shown that in the range of about six to eight carbon atoms; the activity increases with the elongation of the *n*-alkyl carbon chain of 12-*N,N*-di-*n*-alkylaminos, and when there are more than six to eight carbon atoms, the activity decreases with the elongation of the *n*-alkyl carbon chain. This trend is basically the same as that observed in the tetrahydroberberine-12-*N,N*-di-*n*-alkylamine derivatives series (**6a**–**l**), although the activity of the latter series is very weak (Figure 2, Figure 3, Figure 4, Figure 5 and Figure 6). Under this demonstrated SAR, quaternary berberine-12-*N,N*-di-*n*-hexylamine chloride (**7f**) exhibited the most significant growth inhibition activity by IC_50_ value of 0.29 μM for A549, which translated to 22.4 times that of 5-FU and 71.4 times that of QBC (examined as the positive control and substrate comparison, respectively), in the same batch of experiments. The IC_50_ values of quaternary berberine-12-*N,N*-di-*n*-heptylamine chloride (**7g**) inhibiting the growth of HCT-8, Bel7402, and Hela were 0.49 μM, 0.51 μM, and 0.26 μM, respectively, which are the most active compounds for the corresponding cancer cells and are 16.3 times, 11.0 times, and 30.2 times the counterparts of 5-FU and 71.8 times, 51.5 times, and 27 times the counterparts of QBC, respectively. The IC_50_ value of quaternary berberine-12-*N,N*-di-*n*-octylamine chloride (**7h**) as the most active compound against BGC-823, is 0.11 μM, 65.0 times that of 5-FU and 174.5 times that of QBC, respectively.

## 3. Materials and Methods

### 3.1. Chemistry

#### 3.1.1. Reagents and Materials

Nuclear magnetic resonance (NMR) spectra (Figures S1–S81) were recorded on a Varian Mercury-400 NMR spectrometer and reported with tetramethylsilane (TMS) as an internal standard and chloroform-*d* (CDCl_3_) (D, 99.8% + 0.05% *v*/*v* TMS) or dimethyl sulfoxide-*d*_6_ (DMSO*-d*_6_) (D, 99.9% + 0.05% *v*/*v* TMS) (Cambridge Isotope Laboratories, Inc., Andover, MA, USA) as solvents. Chemical shifts (*δ* values) and coupling constants (*J* values) are given in ppm and Hz, respectively. ESIMS^+^ were obtained using an Agilent 1100 series Liquid Chromatograph/Mass Selective Detector (LC/MSD) Trap SL mass spectrometer. QBC was purchased from the market and the purity was determined to be over 98% by high-pressure liquid chromatography (HPLC) and the structure was confirmed on the basis of chemical and spectroscopic data (data not shown). All the reagents and solvents were reagent grade or were purified by standard methods before use. Anhydrous solvents and reagents were all analytically pure and dried through routine protocols. The reaction progress was monitored by thin-layer chromatography (TLC) on a high-efficiency TLC plate with precoated silica gel (GF_254_) produced by Qingdao Haiyang Chemical (Qingdao, China). The spots were visualized by I_2_ steam or under UV light (254 nm). Column chromatography (CC) was carried out with silica gel (200–300 mesh size; Qingdao Haiyang Chemical, Qingdao, China). The concentration of solution after reactions involved the use of a rotary evaporator operated at a reduced pressure of ca. 9.0 mbar.

#### 3.1.2. Synthesis

*Quaternary 12-nitroberberine chloride* (**2**). NaNO_2_ (18.6 g, 269.57 mmol) was added into a reaction bottle containing a solution of QBC (20 g, 53.84 mmol) in acetic acid (250 mL) under the condition of stirring at 0 °C batchwise. Then, concentrated HNO_3_ (30 mL) was added dropwise. The reaction mixture was stirred for 5 min at 0 °C, then heated at 50 °C under stirring for 1h until the raw material was completely reacted. Water (200 mL) was immediately added into the reaction mixture to quench the reaction, and the solution was extracted three times (200 mL/time) using a mixed solution of CHCl_3_/MeOH (*v*/*v* = 10:1) in a separatory funnel. The organic layer was incorporated and concentrated under reduced pressure to remove the solvent. The residue was purified using silica gel CC eluted using a mixed solution of CHCl_3_/CH_3_OH (*v*/*v* = 20:1) to yield **2** (9.65 g, 43% yield) as a red solid. ^1^H-NMR (400 MHz, DMSO-*d*_6_): *δ* 3.23 (t, *J* = 6 Hz, 2H, ArCH_2_CH_2_N), 4.16 (s, 3H, OCH_3_), 4.28 (s, 3H, OCH_3_), 4.96 (t, *J* = 6 Hz, 2H, ArCH_2_CH_2_N), 6.20 (s, 2H, OCH_2_O), 7.13 (s, 1H, Ar–H), 7.83 (s, 1H, Ar–H), 8.89 (s, 1H, Ar–H), 9.05 (s, 1H, Ar–H), 10.12 (s, 1H, Ar–H); ^13^C-NMR: (100 MHz, DMSO-d_6_) δ 26.02, 55.18, 57.67, 62.56, 102.22, 105.70, 108.41, 115.38, 119.90, 120.66, 124.43, 125.18, 131.71, 138.90, 139.94, 147.19, 147.86, 147.90, 149.65, 150.51; ESI–MS (*m*/*z*): 381.2 [M − Cl]^+^.

*Quaternary 12-aminoberberine chloride* (**3**). SnCl_2_·2H_2_O (1173 mg, 5.2 mmol) and concentrated HCl (1.3 mL) were added, in turn, into a solution containing compound **2** (540 mg, 1.3 mol) and absolute ethanol (20 mL) in a reaction bottle under stirring. The reaction mixture was refluxed for 0.5 h under stirring until the raw material was completely reacted according to thin-layer chromatography (TLC) test. The solution was concentrated under reduced pressure to remove the solvent. Aqueous 5% NaOH solution was added into the residue dropwise to make the solution alkaline (pH = 10). The solution was extracted using *n*-butanol three times (20 mL/time) in a separatory funnel. The organic layer was integrated and concentrated under reduced pressure to yield a residue, which was purified using silica gel CC eluted using a mixed solvent of CHCl_3_/CH_3_OH (*v*/*v* = 15:1) to yield **3** (200 mg, 40% yield) as a red solid. ^1^H-NMR (400 MHz, DMSO-*d*_6_): *δ* 3.18 (t, *J* = 6 Hz, 2H, ArCH_2_CH_2_N), 3.89 (s, 3H, OCH_3_), 3.96 (s, 3H, OCH_3_), 4.87 (t, *J* = 6 Hz, 2H, ArCH_2_CH_2_N), 6.16 (s, 2H, OCH_2_O), 6.62 (s, 2H, Ar–NH_2_), 7.06 (s, 1H, Ar–H), 7.14 (s, 1H, Ar–H), 7.88 (s, 1H, Ar–H), 8.85 (s, 1H, Ar–H), 9.61 (s, 1H, Ar–H); ^13^C-NMR: (100 MHz, DMSO-*d*_6_) *δ* 26.28, 54.82, 56.17, 61.74, 101.88, 104.10, 105.12, 108.31, 116.10, 120.09, 120.89, 121.44, 129.82, 133.21, 134.72, 143.19, 144.50, 147.49, 149.18, 152.32; ESI–MS (*m*/*z*): 351.5 [M − Cl]^+^.

*Quaternary berberine-12-N-p-trifluoromethylphenylcarbonylamine chloride* (**4a**). Pyridine (63 μL, 0.78 mmol) was added into a solution containing compound **3** (150 mg, 0.39 mmol) in anhydrous CH_2_Cl_2_ (6 mL) in a reaction bottle under stirring. The reaction mixture was stirred at 0°C for 10 min. Then, *p*-trifluoromethylbenzoyl chloride (63 μL, 0.429 mmol) was added into the reaction mixture, and the reaction solution was stirred at room temperature for 8 h until the raw material was completely reacted according to TLC analysis. After adding a small amount of water (10 mL), the solution was extracted three times (20 mL/time) using *n*-butanol in a separatory funnel. The organic layer was integrated, dried using anhydrous MgSO_4_, and filtered. The filtrate was concentrated under reduced pressure to remove the solvent. The residue was purified using silica gel CC eluted using a mixed solvent of CHCl_3_/CH_3_OH (*v*/*v* = 20:1) to yield **4a** (60 mg, 28% yield) as a yellow amorphous solid. ^1^H-NMR (400 MHz, DMSO-*d*_6_): *δ* 3.21 (t, *J* = 6 Hz, 2H, ArCH_2_CH_2_N), 4.07 (s, 3H, OCH_3_), 4.11 (s, 3H, OCH_3_), 4.95 (t, *J* = 6 Hz, 2H, ArCH_2_CH_2_N), 6.15 (s, 2H, OCH_2_O), 7.10 (s, 1H, Ar–H), 7.96 (s, 1H, Ar–H), 8.01 (d, *J* = 8 Hz, 2H, Ar–H), 8.39 (d, *J* = 8 Hz, 2H, Ar–H), 8.41 (s, 1H, Ar–H), 8.78 (s, 1H, Ar–H), 9.95 (s, 1H, Ar–H), 11.17 (s, 1H, ArNHCO); ESI–MS (*m*/*z*): 523.2 [M − Cl]^+^.

*Quaternary berberine-12-N-methylcarbonylamine chloride* (**4b**). Target compound **4b** was obtained (51 mg, 51% yield) as a yellow amorphous solid from compound **3** (90 mg, 0.233 mmol), pyridine (75 μL, 0.932 mmol), and acetyl chloride (36 μL, 0.513 mmol) using a procedure similar to that for synthesizing compound **4a**. ^1^H-NMR (400 MHz, DMSO-*d*_6_): *δ* 2.31 (s, 3H, NHCOCH_3_), 3.21 (t, *J* = 6 Hz, 2H, ArCH_2_CH_2_N), 4.03 (s, 3H, OCH_3_), 4.06 (s, 3H, OCH_3_), 4.93 (t, *J* = 6 Hz, 2H, ArCH_2_CH_2_N), 6.18 (s, 2H, OCH_2_O), 7.10 (s, 1H, Ar–H), 8.01 (s, 1H, Ar–H), 8.47 (s, 1H, Ar–H), 8.88 (s, 1H, Ar–H), 9.87 (s, 1H, Ar–H), 10.54 (s, 1H, ArNHCO); ^13^C-NMR: (100 MHz, DMSO-*d*_6_) *δ* 23.99, 26.31, 55.14, 56.93, 62.03, 102.14, 105.90, 108.49, 116.00, 119.30, 120.62, 121.22, 126.03, 130.78, 130.82, 136.98, 140.42, 145.62, 147.68, 149.86, 150.09, 169.59; ESI–MS (*m*/*z*): 393.3 [M − Cl]^+^.

*Quaternary berberine-12-N-isopropylcarbonylamine chloride* (**4c**). Target compound **4c** was obtained (31 mg, 59.6% yield) as a yellow amorphous solid from compound **3** (44 mg, 0.114 mmol), pyridine (37 μL, 0.456 mmol), and isobutyryl chloride (26 μL, 0.251 mmol) using a procedure similar to that for synthesizing **4a**. ^1^H-NMR (400 MHz, DMSO-*d*_6_): *δ* 1.22 (d, *J* = 6.8 Hz, 6H, NHCOCHC_2_H_6_), 3.08 (septet, 1H, *J* = 6.8 Hz, NHCOCHC_2_H_6_), 3.20 (t, *J* = 6 Hz, 2H, ArCH_2_CH_2_N), 4.04 (s, 3H, OCH_3_), 4.06 (s, 3H, OCH_3_), 4.93 (t, *J* = 6 Hz, 2H, ArCH_2_CH_2_N), 6.18 (s, 2H, OCH_2_O), 7.10 (s, 1H, Ar–H), 8.02 (s, 1H, Ar–H), 8.51 (s, 1H, Ar–H), 8.91 (s, 1H, Ar–H), 9.88 (s, 1H, Ar–H), 10.58 (s, 1H, ArNHCO); ^13^C-NMR: (100 MHz, DMSO-*d*_6_) *δ* 19.62 (2 × C), 26.31, 34.53, 55.11, 56.92, 62.03, 102.12, 105.85, 108.46, 116.05, 119.10, 120.67, 121.22, 125.92, 130.80, 130.87, 136.88, 140.29, 145.56, 147.68, 149.85, 150.07, 176.48; ESI–MS (*m*/*z*): 421.3 [M − Cl]^+^.

*Quaternary berberine-12-N-p-bromophenylcarbonylamine chloride* (**4d**). Target compound **4d** was obtained (84 mg, 56.9% yield) as a yellow amorphous solid from compound **3** (100 mg, 0.259 mmol), pyridine (83 μL, 1.036 mmol), and *p*-bromobenzoyl chloride (125μL, 0.57 mmol) using a procedure similar to that for **4a**. ^1^H-NMR (400 MHz, DMSO-*d*_6_): *δ* 3.21 (t, *J* = 6 Hz, 2H, ArCH_2_CH_2_N), 4.06 (s, 3H, OCH_3_), 4.11 (s, 3H, OCH_3_), 4.95 (t, *J* = 6 Hz, 2H, ArCH_2_CH_2_N), 6.15 (s, 2H, OCH_2_O), 7.10 (s, 1H, Ar–H), 7.83 (d, *J* = 8 Hz, 2H, Ar–H), 7.93 (s, 1H, Ar–H), 8.13 (d, *J* = 8 Hz, 2H, Ar–H), 8.39 (s, 1H, Ar–H), 8.75 (s, 1H, Ar–H), 9.93 (s, 1H, Ar–H), 10.94 (s, 1H, ArNHCO); ^13^C-NMR: (100 MHz, DMSO-*d*_6_) *δ* 26.32, 55.20, 57.11, 62.08, 102.06, 106.09, 108.41, 116.89, 120.60, 121.26, 122.39, 125.85, 127.66, 130.44 (2 × C), 130.64, 130.83, 131.44 (2 × C), 133.22, 136.93, 141.64, 145.82, 147.60, 149.81, 149.99,165.84; ESI–MS (*m*/*z*): 535.0 [M − Cl]^+^.

*Quaternary berberine-12-N-phenylcarbonylamine chloride* (**4e**). Target compound **4e** was obtained (74 mg, 58.3% yield) as a yellow amorphous solid from compound **3** (100 mg, 0.259 mmol), pyridine (83 μL, 1.036 mmol), and benzoyl chloride (66 μL, 0.57 mmol) using a procedure similar to that for **4a**. ^1^H-NMR (400 MHz, DMSO-*d*_6_): *δ* 3.22 (t, *J* = 6 Hz, 2H, ArCH_2_CH_2_N), 4.07 (s, 3H, OCH_3_), 4.11 (s, 3H, OCH_3_), 4.95 (t, *J* = 6 Hz, 2H, ArCH_2_CH_2_N), 6.15 (s, 2H, OCH_2_O), 7.10 (s, 1H, Ar–H), 7.62 (t, *J* = 7.6 Hz, 2H, Ar–H), 7.66 (t, *J* = 7.2 Hz, 1H, Ar–H) 7.91 (s, 1H, Ar–H), 8.17 (m, 2H, Ar–H), 8.43 (s, 1H, Ar–H), 8.74 (s, 1H, Ar–H), 9.93 (s, 1H, Ar–H), 10.80 (s, 1H, ArNHCO); ^13^C-NMR: (100 MHz, DMSO-*d*_6_) *δ* 26.33, 55.19, 57.09, 62.09, 102.06, 106.02, 108.42, 116.89, 120.63, 121.27, 122.09, 127.53, 128.32 (2 × C), 128.46(2 × C), 130.83, 130.89, 132.07, 134.11, 136.87, 141.47, 145.78, 147.61, 149.80, 150.02, 166.75; ESI-MS (*m*/*z*): 455.2 [M − Cl]^+^.

*Quaternary berberine-12-N-p-fluorophenylcarbonylamine chloride* (**4f**). Target compound **4f** was obtained (46 mg, 35% yield) as a yellow amorphous solid from compound **3** (100 mg, 0.259 mmol), pyridine (83 μL, 1.036 mmol), and *p*-fluorobenzoyl chloride (68 μL, 0.57 mmol) using a procedure similar to that for **4a**. ^1^H-NMR (400 MHz, DMSO-*d*_6_): *δ* 3.21 (t, *J* = 6 Hz, 2H, ArCH_2_CH_2_N), 4.06 (s, 3H, OCH_3_), 4.11 (s, 3H, OCH_3_), 4.95 (t, *J* = 6 Hz, 2H, ArCH_2_CH_2_N), 6.15 (s, 2H, OCH_2_O), 7.09 (s, 1H, Ar–H), 7.46 (t, *J* = 8.8 Hz, 2H, Ar–H), 7.93 (s, 1H, Ar–H), 8.26 (m, 2H, Ar–H), 8.40 (s, 1H, Ar–H), 8.76 (s, 1H, Ar–H), 9.93 (s, 1H, Ar–H), 10.88 (s, 1H, ArNHCO); ^13^C-NMR: (150 MHz, DMSO-*d*_6_) *δ* 26.33, 55.17, 57.09, 62.07, 102.02, 106.12, 108.36, 115.32 (d, *J*=21 Hz, 2C), 117.08, 120.63, 121.24, 122.39, 127.64, 130.48, 130.50, 130.78, 131.17 (d, *J*=9 Hz, 2C), 136.80, 141.52, 145.70, 147.58, 149.76, 149.96, 164.37 (d, *J* = 249 Hz, 1C), 165.62; ESI–MS (*m*/*z*): 473.2 [M − Cl]^+^.

*Quaternary berberine-12-N-p-methylphenylcarbonylamine chloride* (**4g**). Target compound **4g** was obtained (54 mg, 41.3% yield) as a yellow amorphous solid from compound **3** (100 mg, 0.259 mmol), pyridine (83 μL, 1.036 mmol), and *p*-methylbenzoyl chloride (76 μL, 0.57 mmol) using a procedure similar to that for **4a**. ^1^H-NMR (400 MHz, DMSO-*d*_6_): *δ* 2.44 (s, 3H, NHCOArCH_3_), 3.21 (t, *J* = 6 Hz, 2H, ArCH_2_CH_2_N), 4.06 (s, 3H, OCH_3_), 4.11 (s, 3H, OCH_3_), 4.95 (t, *J* = 6 Hz, 2H, ArCH_2_CH_2_N), 6.15 (s, 2H, OCH_2_O), 7.10 (s, 1H, Ar–H), 7.42 (d, *J* = 8 Hz, 2H, Ar–H), 7.88 (s, 1H, Ar–H), 8.06 (d, *J* = 8 Hz, 2H, Ar–H), 8.41 (s, 1H, Ar–H), 8.71 (s, 1H, Ar–H), 9.92 (s, 1H, Ar–H), 10.70 (s, 1H, ArNHCO); ^13^C-NMR: (150 MHz, DMSO-*d*_6_) *δ* 21.08, 26.30, 55.14, 57.05, 62.04, 102.01, 105.93, 108.37, 116.91, 120.59, 121.22, 122.05, 127.48, 128.32 (2 × C), 128.90 (2 × C) 130.77, 130.94, 131.20, 136.74, 141.34, 142.09, 145.68, 147.57, 149.75, 149.98, 166.53; ESI–MS (*m*/*z*): 469.1 [M − Cl]^+^.

*Quaternary berberine-12-N-m-methylphenylcarbonylamine chloride* (**4h**). Target compound **4h** was obtained (90 mg, 68.8% yield) as a yellow amorphous solid from compound **3** (100 mg, 0.259 mmol), pyridine (83 μL, 1.036 mmol), and *m*-methylbenzoyl chloride (75 μL, 0.57 mmol) using a procedure similar to that for **4a**. ^1^H-NMR (400 MHz, DMSO-*d*_6_): *δ* 2.45 (s, 3H, NHCOArCH_3_), 3.21 (t, *J* = 6 Hz, 2H, ArCH_2_CH_2_N), 4.06 (s, 3H, OCH_3_), 4.11 (s, 3H, OCH_3_), 4.95 (t, *J* = 6 Hz, 2H, ArCH_2_CH_2_N), 6.15 (s, 2H, OCH_2_O), 7.09 (s, 1H, Ar–H), 7.49 (m, 2H, Ar–H), 7.91 (s, 1H, Ar–H), 7.98 (m, 2H, Ar–H), 8.39 (s, 1H, Ar–H), 8.74 (s, 1H, Ar–H), 9.93 (s, 1H, Ar–H), 10.82 (s, 1H, ArNHCO); ^13^C-NMR: (100 MHz, DMSO-*d*_6_) *δ* 21.02, 26.33, 55.18, 57.08, 62.08, 102.05, 106.00, 108.41, 116.96, 120.63, 121.27, 122.15, 125.47, 127.57, 128.35, 128.80, 130.82, 130.95, 132.61, 134.09, 136.83, 137.74, 141.47, 145.78, 147.61, 149.80, 150.02, 166.86; ESI–MS (*m*/*z*): 469.2 [M − Cl]^+^.

*Quaternary berberine-12-N-ethylcarbonylamine chloride* (**4i**). Target compound **4i** was obtained (46 mg, 40.1% yield) as a yellow amorphous solid from compound **3** (100 mg, 0.259 mmol), pyridine (83 μL, 1.036 mmol), and propionyl chloride (50 μL, 0.57 mmol) using a procedure similar to that for **4a**. ^1^H-NMR (400 MHz, DMSO-*d*_6_): *δ* 1.18 (t, *J* = 7.6 Hz, 3H, NHCOCH_2_CH_3_), 2.67 (q, *J* = 7.6 Hz, 2H, NHCOCH_2_CH_3_), 3.21 (t, *J* = 6 Hz, 2H, ArCH_2_CH_2_N), 4.03 (s, 3H, OCH_3_), 4.06 (s, 3H, OCH_3_), 4.93 (t, *J* = 6 Hz, 2H, ArCH_2_CH_2_N), 6.18 (s, 2H, OCH_2_O), 7.09 (s, 1H, Ar-H), 8.02 (s, 1H, Ar-H), 8.51 (s, 1H, Ar–H), 8.91 (s, 1H, Ar–H), 9.87 (s, 1H, Ar–H), 10.53 (s, 1H, ArNHCO); ^13^C-NMR: (150 MHz, DMSO-*d*_6_) *δ* 9.48, 26.30, 29.22, 55.10, 56.88, 62.00, 102.06, 106.09, 108.37, 116.24, 118.93, 120.67, 121.18, 125.85, 130.69, 130.95, 136.85, 140.13, 145.44, 147.63, 149.77, 150.04, 173.27; ESI–MS (*m*/*z*): 407.2 [M − Cl]^+^.

*Quaternary berberine-12-N-n-butylcarbonylamine chloride* (**4j**). Target compound **4j** was obtained (60 mg, 49.2% yield) as a yellow amorphous solid from compound **3** (100 mg, 0.259 mmol), pyridine (83 μL, 1.036 mmol), and *n*-pentanoyl chloride (68 μL, 0.57 mmol) using a procedure similar to that for **4a**. ^1^H-NMR (400 MHz, DMSO-*d*_6_): *δ* 0.95 (t, *J* = 7.6 Hz, 3H, NHCOCH_2_CH_2_CH_2_CH_3_), 1.42 (m, 2H, NHCOCH_2_CH_2_CH_2_CH_3_), 1.68 (m, 2H, NHCOCH_2_CH_2_CH_2_CH_3_), 2.65 (t, *J* = 7.6 Hz, 2H, NHCOCH_2_CH_2_CH_2_CH_3_), 3.21 (t, *J* = 6 Hz, 2H, ArCH_2_CH_2_N), 4.03 (s, 3H, OCH_3_), 4.06 (s, 3H, OCH_3_), 4.93 (t, *J* = 6 Hz, 2H, ArCH_2_CH_2_N), 6.18 (s, 2H, OCH_2_O), 7.10 (s, 1H, Ar–H), 8.02 (s, 1H, Ar–H), 8.50 (s, 1H, Ar–H), 8.90 (s, 1H, Ar–H), 9.87 (s, 1H, Ar–H), 10.60 (s, 1H, ArNHCO); ^13^C-NMR: (100 MHz, DMSO-*d*_6_) *δ* 13.86, 21.89, 26.30, 27.09, 35.73, 55.11, 56.91, 62.02, 102.12, 105.89, 108.45, 116.09, 119.11, 120.65, 121.21, 125.93, 130.77, 130.85, 136.90, 140.29, 145.55, 147.67, 149.83, 150.08, 172.53; ESI–MS (*m*/*z*): 435.3 [M − Cl]^+^.

*Quaternary berberine-12-N-p-methoxyphenylcarbonylamine chloride* (**4k**). Target compound **4k** was obtained (60 mg, 44.4% yield) as a yellow amorphous solid from compound **3** (100 mg, 0.259 mmol), pyridine (83 μL, 1.036 mmol), and *p*-methoxybenzoyl chloride (97 mg, 0.57 mmol) using a procedure similar to that for **4a**. ^1^H-NMR (400 MHz, DMSO-*d*_6_): *δ* 3.21 (t, *J* = 6 Hz, 2H, ArCH_2_CH_2_N), 3.88 (s, 3H, NHCOArOCH_3_), 4.06 (s, 3H, OCH_3_), 4.10 (s, 3H, OCH_3_), 4.95 (t, *J* = 6 Hz, 2H, ArCH_2_CH_2_N), 6.15 (s, 2H, OCH_2_O), 7.09 (s, 1H, Ar–H), 7.14 (d, *J* = 8.8 Hz, 2H, Ar–H), 7.92 (s, 1H, Ar–H), 8.19 (d, *J* = 8.8 Hz, 2H, Ar–H), 8.40 (s, 1H, Ar–H), 8.75 (s, 1H, Ar–H), 9.92 (s, 1H, Ar–H), 10.77 (s, 1H, ArNHCO); ^13^C-NMR: (100 MHz, DMSO-*d*_6_) *δ* 26.35, 55.17, 55.54, 57.06, 62.07, 102.05, 106.00, 108.42, 113.67 (2 × C), 117.00, 120.66, 121.26, 121.96, 126.12, 127.49, 130.34 (2 × C), 130.81, 131.13, 136.73, 141.27, 145.71, 147.62, 149.78, 150.04, 162.33, 166.08; ESI–MS (*m*/*z*): 485.2 [M − Cl]^+^.

*Quaternary berberine-12-N-p-nitrophenylcarbonylamine chloride* (**4l**)**.** Target compound **4l** was obtained (36 mg, 26% yield) as a yellow amorphous solid from compound **3** (100 mg, 0.259 mmol), pyridine (83 μL, 1.036 mmol), and *p*-nitrobenzoyl chloride (106 mg, 0.57 mmol) using a procedure similar to that for **4a**. ^1^H-NMR (400 MHz, DMSO-*d*_6_): *δ* 3.22 (t, *J* = 6 Hz, 2H, ArCH_2_CH_2_N), 4.07 (s, 3H, OCH_3_), 4.12 (s, 3H, OCH_3_), 4.95 (t, *J* = 6 Hz, 2H, ArCH_2_CH_2_N), 6.15 (s, 2H, OCH_2_O), 7.09 (s, 1H, Ar–H), 7.93 (s, 1H, Ar–H), 8.39-8.47 (m, 5H, Ar–H), 8.77 (s, 1H, Ar–H), 9.94 (s, 1H, Ar–H), 11.17 (s, 1H, ArNHCO); ^13^C-NMR: (100 MHz, DMSO-*d*_6_) *δ* 26.32, 55.23, 57.16, 62.11, 102.07, 106.11, 108.42, 116.82, 120.56, 121.27, 122.69, 123.55 (2 × C), 127.77, 129.85 (2 × C), 130.28, 130.86, 137.07, 139.87, 141.93, 145.92, 147.60, 149.42, 149.84, 149.98, 165.22; ESI–MS (*m*/*z*): 500.2 [M − Cl]^+^.

*Quaternary berberine-12-N-t-butylcarbonylamine chloride* (**4m**). Target compound **4m** was obtained (30 mg, 24.6% yield) as a yellow amorphous solid from compound **3** (100 mg, 0.259 mmol), pyridine (83 μL, 1.036 mmol), and pivaloyl chloride (70 μL, 0.57 mmol) using a procedure similar to that for **4a**. ^1^H-NMR (400 MHz, DMSO-*d*_6_): *δ* 1.39 (s, 9H, NHCOC(CH_3_)_3_), 3.21 (t, *J* = 6 Hz, 2H, ArCH_2_CH_2_N), 4.05 (s, 3H, OCH_3_), 4.09 (s, 3H, OCH_3_), 4.94 (t, *J* = 6 Hz, 2H, ArCH_2_CH_2_N), 6.18 (s, 2H, OCH_2_O), 7.11 (s, 1H, Ar–H), 7.68 (s, 1H, Ar–H), 8.11 (s, 1H, Ar–H), 8.38 (s, 1H, Ar–H), 9.91 (s, 1H, Ar–H), 10.02 (s, 1H, ArNHCO); ^13^C-NMR: (100 MHz, DMSO-*d*_6_) *δ* 26.33, 27.30 (3 × C), 55.08, 57.13, 62.06, 102.14, 105.12, 108.53, 116.54, 120.54, 121.22, 123.06, 127.87, 130.77, 130.96, 136.58, 141.50, 145.85, 147.77, 149.91, 150.03, 177.67; ESI–MS (*m*/*z*): 435.3 [M − Cl]^+^.

*12-Aminotetrahydroberberine* (**5**). NiCl_2_·6H_2_O (855.7 mg, 3.6 mmol) was added into a solution containing compound **2** (300 mg, 0.72 mmol) in a mixed solution of THF/MeOH (12 mL, *v*/*v* = 1:1). Then, NaBH_4_ (272 mg, 7.2 mmol) was added batchwise. The reaction was performed at 66 °C for 20 min under stirring until the raw material was completely reacted. The reaction mixture was filtered. The filtrate was evaporated under reduced pressure. The residue was dissolved in CH_2_Cl_2_ solvent. The solution was washed in a separatory funnel, first, three times using water, then one time using saturated aqueous NaCl solution. The organic layer was dried using anhydrous MgSO_4_, and then filtered. The filtrate was concentrated under reduced pressure to yield compound **5** (213 mg, 83.5% yield) as a hazel amorphous solid. ^1^H-NMR (400 MHz, DMSO-*d*_6_): *δ* 2.07–3.34 (m, 8H), 3.59 (s, 3H, OCH_3_), 3.69 (s, 3H, OCH_3_), 3.96 (d, *J* = 15.2 Hz, 1H), 4.68 (s, 2H, ArNH_2_), 5.94, 5.96 (2 × br s, 2H, OCH_2_O), 6.24 (s, 1H, Ar-H), 6.66 (s, 1H, Ar-H), 6.96 (s, 1H, Ar-H); ^13^C-NMR: (100 MHz, DMSO-*d*_6_) *δ* 28.96, 31.63, 50.95, 53.67, 55.29, 59.09, 59.72, 97.39, 100.52, 105.90, 108.00, 110.63, 127.40, 128.07, 131.40, 135.26, 142.24, 145.36, 145.64, 150.08; ESI–MS (*m*/*z*): 355.3 [M + H]^+^.

*Tetrahydroberberine-12-N,N-dimethylamine* (**6a**)**.** To a stirred solution of compound **5** (200 mg, 0.564 mmol) in CH_2_Cl_2_ (8 mL), we added aqueous 37% formaldehyde (186 μL, 2.48 mmol), sodium triacetoxyborohydride (597 mg, 2.82 mmol) and HOAc (16 drops). The reaction mixture was stirred for 2h at room temperature until the raw material was completely reacted. A small amount of saturated aqueous NaHCO_3_ solution was added into the reaction mixture dropwise to make the solution alkaline (pH = 8). The solution was stirred for 2h at room temperature, and then extracted using CH_2_Cl_2_ three times in a separatory funnel. The organic layer was washed, first, three times using water, then, one time using saturated aqueous NaCl solution. Then, the organic layer was dried with anhydrous MgSO_4_ and filtered. The filtrate was concentrated under reduced pressure. The residue was purified using silica gel CC eluted using a mixed solvent of CHCl_3_/CH_3_OH (*v*/*v* = 80:1) to yield **6a** (205 mg, 95% yield) as a hazel solid. ^1^H-NMR (400 MHz, DMSO-*d*_6_): *δ* 2.32–3.41 (m, 8H), 2.59 (s, 6H, N(CH_3_)_2_), 3.68 (s, 3H, OCH_3_), 3.79 (s, 3H, OCH_3_), 4.04 (d, *J* = 16 Hz, 1H), 5.94, 5.96 (2 × br s, 2H, OCH_2_O), 6.65 (s, 1H, Ar–H), 6.68 (s, 1H, Ar–H), 6.86 (s, 1H, Ar–H); ^13^C-NMR (100 MHz, DMSO-*d*_6_): *δ* 29.08, 33.45, 44.08 (2 × C), 50.54, 53.80, 55.67, 58.96, 59.51, 100.57, 102.32, 105.77, 108.11, 121.61, 127.63, 128.89, 131.05, 140.06, 145.46, 145.70, 148.09, 149.84; ESI–MS (*m*/*z*): 383.4 [M + H]^+^.

*Tetrahydroberberine-12-N,N-diethylamine* (**6b**). Target compound **6b** was obtained (277 mg, 96% yield) as a hazel solid from compound **5** (250 mg, 0.705 mmol), aqueous 40% acetaldehyde (313 μL, 3.1 mmol), sodium triacetoxyborohydride (747 mg, 3.525 mmol), and HOAc (16 drops) using a procedure similar to that for **6a**. ^1^H-NMR (400 MHz, CDCl_3_): *δ* 0.98 (t, *J* = 7.2 Hz, 6H, N(CH_2_CH_3_)_2_), 2.47-2.68 (m, 3H), 2.94 (t, *J* = 7.2 Hz, 4H, N(CH_2_CH_3_)_2_), 3.11-3.21 (m, 2H), 3.41–3.56 (m, 3H), 3.82 (s, 3H, OCH_3_), 3.84 (s, 3H, OCH_3_), 4.23 (d, *J* = 16 Hz,1H), 5.93 (s, 2H, OCH_2_O), 6.60 (s, 1H, Ar–H), 6.61 (s, 1H, Ar–H), 6.82 (s, 1H, Ar–H); ^13^C-NMR: (100 MHz, CDCl_3_) *δ* 12.76 (2 × C), 29.71, 33.76, 47.89 (2 × C), 51.60, 54.53, 56.09, 59.91, 60.33, 100.89, 105.68, 105.97, 108.50, 125.29, 127.93, 129.02, 131.41, 141.47, 145.51, 145.99, 146.23, 150.29; ESI–MS (*m*/*z*): 411.3 [M + H]^+^.

*Tetrahydroberberine-12-N,N-di-n-propylamine* (**6c**). Target compound **6c** was obtained (200 mg, 54% yield) as a hazel solid from compound **5** (300 mg, 0.846 mmol), propionaldehyde (147.4 μL, 2.03 mmol), sodium triacetoxyborohydride (537.8 mg, 2.538 mmol), and HOAc (12 drops) using a procedure similar to that for **6a**. ^1^H-NMR (400 MHz, CDCl_3_): *δ* 0.83 (t, *J* = 7.2 Hz, 6H, N(CH_2_CH_2_CH_3_)_2_), 1.43 (m, 4H), 2.51 (m, 1H), 2.61 (m, 1H), 2.67 (ov, 1H), 2.82 (m, 4H), 3.13 (ov, 1H), 3.19 (ov, 1H), 3.40 (d, *J* = 10.0 Hz, 1H), 3.49 (m, 1H), 3.53 (d, *J* = 16.0 Hz), 3.82 (s, 3H, 10-OCH_3_), 3.83 (s, 3H, 9-OCH_3_), 4.23 (d, *J* = 16.0 Hz, 1H), 5.92, 5.93 (2 × br s, 2H, OCH_2_O), 6.60 (s, 1H, Ar–H), 6.62 (s, 1H, Ar–H), 6.78 (s, 1H, Ar–H); ^13^C-NMR: (100 MHz, CDCl_3_) *δ* 11.92 (2 × C), 20.53 (2 × C), 29.73, 33.79, 51.64, 54.57, 56.11 (3 × C), 59.93, 60.32, 100.88, 105.71, 105.86, 108.51, 124.97, 127.96, 128.98, 131.39, 141.38, 145.98, 146.26 (2 × C), 150.28; ESI–MS (*m*/*z*): 439.3 [M + H]^+^.

*Tetrahydroberberine-12-N,N-di-n-butylamine* (**6d**)**.** Target compound **6d** was obtained (287 mg, 87.2% yield) as a hazel solid from compound **5** (250 mg, 0.705 mmol), *n*-butyraldehyde (152 μL, 1.69 mmol), sodium triacetoxyborohydride (448 mg, 2.115 mmol), and HOAc (10 drops) using a procedure similar to that for **6a**. ^1^H-NMR (400 MHz, DMSO-*d*_6_): *δ* 0.83 (t, *J* = 7.2 Hz, 6H, N(CH_2_CH_2_CH_2_CH_3_)_2_), 1.21–1.40 (m, 8H), 2.24–3.39 (m, 8H), 2.83 (t, *J* = 6.8 Hz, 4H, N(CH_2_CH_2_CH_2_CH_3_)_2_), 3.70 (s, 3H, OCH_3_), 3.76 (s, 3H, OCH_3_), 4.04 (d, *J* = 16 Hz, 1H), 5.96 (s, 2H, OCH_2_O), 6.68 (s, 1H, Ar-H), 6.73 (br s, 2H, Ar–H); ^13^C-NMR: (100 MHz, DMSO-*d*_6_) *δ* 13.92 (2 × C), 19.93 (2 × C), 28.98, 29.12 (2 × C), 33.34, 50.63, 53.36 (2 × C), 53.71, 55.73, 58.97, 59.51, 100.62, 105.17, 105.55, 108.20, 124.41, 127.63, 128.58, 131.10, 140.65, 145.47, 145.55, 145.73, 149.85; ESI–MS (*m*/*z*): 467.3 [M + H]^+^.

*Tetrahydroberberine-12-N,N-di-n-pentylamine* (**6e**). Target compound **6e** was obtained (320 mg, 91.8% yield) as a hazel oil from compound **5** (250 mg, 0.705 mmol), *n*-valeraldehyde (180 μL, 1.69 mmol), sodium triacetoxyborohydride (448 mg, 2.115 mmol), and HOAc (10 drops) using a procedure similar to that for **6a**. ^1^H-NMR (400 MHz, CDCl_3_): *δ* 0.85 (t, *J* = 6.8 Hz, 6H, N(CH_2_CH_2_CH_2_CH_2_CH_3_)_2_), 1.24 (m, 8H), 1.40 (m, 4H), 2.46–2.68 (m, 3H), 2.85 (t, *J* = 7.2 Hz, 4H, N(CH_2_CH_2_CH_2_CH_2_CH_3_)_2_), 3.11–3.20 (m, 2H), 3.39-3.55 (m, 3H), 3.83 (br s, 6H, OCH_3_), 4.23 (d, *J* = 15.6 Hz,1H), 5.92 (s, 2H, OCH_2_O), 6.60 (s, 1H, Ar–H), 6.62 (s, 1H, Ar–H), 6.79 (s, 1H, Ar-H); ^13^C-NMR: (100 MHz, CDCl_3_) *δ* 14.30 (2 × C), 22.74 (2 × C), 27.06 (2 × C), 29.72, 29.79 (2 × C), 33.79, 51.67, 54.19 (2 × C), 54.55, 56.09, 59.91, 60.33, 100.89, 105.65, 105.88, 108.50, 124.94, 127.93, 128.95, 131.41, 141.33, 145.98, 146.29 (2 × C), 150.26; ESI–MS (*m*/*z*): 495.4 [M + H]^+^.

*Tetrahydroberberine-12-N,N-di-n-hexylamine* (**6f**). Target compound **6f** was obtained (230 mg, 78% yield) as a hazel oil from compound **5** (200 mg, 0.564 mmol), *n*-hexanal (164 μL, 1.354 mmol), sodium triacetoxyborohydride (358 mg, 1.692 mmol), and HOAc (10 drops) using a procedure similar to that for **6a**. ^1^H-NMR (400 MHz, CDCl_3_): *δ* 0.85 (t, *J* = 6.8 Hz, 6H, N(CH_2_CH_2_CH_2_CH_2_CH_2_CH_3_)_2_), 1.23 (m, 12H), 1.40 (m, 4H), 2.46-2.68 (m, 3H), 2.85 (t, *J* = 7.2 Hz, 4H, N(CH_2_CH_2_CH_2_CH_2_CH_2_CH_3_)_2_)), 3.12–3.20 (m, 2H), 3.39–3.56 (m, 3H), 3.82 (s, 3H, OCH_3_), 3.83 (s, 3H, OCH_3_), 4.23 (d, *J* = 15.6 Hz,1H), 5.92 (s, 2H, OCH_2_O), 6.60 (s, 1H, Ar-H), 6.62 (s, 1H, Ar-H), 6.79 (s, 1H, Ar-H); ^13^C-NMR: (100 MHz, CDCl_3_) *δ* 14.17 (2 × C), 22.84 (2 × C), 27.22 (2 × C), 27.33 (2 × C), 29.69, 31.88 (2 × C), 33.77, 51.63, 54.21 (2 × C), 54.52, 56.08, 59.89, 60.32, 100.88, 105.64, 105.87, 108.48, 124.88, 127.89, 128.92, 131.38, 141.31, 145.99, 146.29 (2 × C), 150.25; ESI–MS (*m*/*z*): 523.4 [M + H]^+^.

*Tetrahydroberberine-12-N,N-di-n-heptylamine* (**6g**). Target compound **6g** was obtained (272 mg, 87.6% yield) as a hazel oil from compound **5** (200 mg, 0.564 mmol), *n*-heptanal (189 μL, 1.354 mmol), sodium triacetoxyborohydride (358 mg, 1.692 mmol), and HOAc (10 drops) using a procedure similar to that for **6a**. ^1^H-NMR (400 MHz, CDCl_3_): *δ* 0.85 (t, *J* = 6.8 Hz, 6H, N(CH_2_CH_2_(CH_2_)_4_CH_3_)_2_)), 1.23 (m, 16H), 1.40 (m, 4H), 2.46–2.68 (m, 3H), 2.85 (t, *J* = 7.2 Hz, 4H, N(CH_2_CH_2_(CH_2_)_4_CH_3_)_2_)), 3.12–3.21 (m, 2H), 3.39-3.55 (m, 3H), 3.82 (s, 3H, OCH_3_), 3.83 (s, 3H, OCH_3_), 4.23 (d, *J* = 16 Hz,1H), 5.92 (s, 2H, OCH_2_O), 6.60 (s, 1H, Ar–H), 6.62 (s, 1H, Ar–H), 6.79 (s, 1H, Ar–H); ^13^C-NMR: (100 MHz, CDCl_3_) *δ* 14.20 (2 × C), 22.75 (2 × C), 27.39 (2 × C), 27.52 (2 × C), 29.36 (2 × C), 29.68, 32.05 (2 × C), 33.74, 51.63, 54.21 (2 × C), 54.51, 56.08, 59.89, 60.32, 100.88, 105.65, 105.87, 108.48, 124.90, 127.88, 128.89, 131.36, 141.32, 146.01, 146.30 (2 × C), 150.26; ESI–MS (*m*/*z*): 551.4 [M + H]^+^.

*Tetrahydroberberine-12-N,N-di-n-octylamine* (**6h**). Target compound **6h** was obtained (290 mg, 88.8% yield) as a hazel oil from compound **5** (200 mg, 0.564 mmol), *n*-octanal (300 μL, 1.92 mmol), sodium triacetoxyborohydride (478 mg, 2.256 mmol), and HOAc (13 drops) using a procedure similar to that for **6a**. ^1^H-NMR (400 MHz, CDCl_3_): *δ* 0.85 (t, *J* = 6.8 Hz, 6H, N(CH_2_CH_2_(CH_2_)_5_CH_3_)**_2_**), 1.23 (m, 20H), 1.40 (m, 4H), 2.46-2.68 (m, 3H), 2.85 (t, *J* = 7.2 Hz, 4H, N(CH_2_CH_2_(CH_2_)_5_CH_3_)_2_), 3.12-3.21 (m, 2H), 3.39–3.55 (m, 3H), 3.82 (s, 3H, OCH_3_), 3.83 (s, 3H, OCH_3_), 4.23 (d, *J* = 16 Hz, 1H), 5.92 (s, 2H, OCH_2_O), 6.60 (s, 1H, Ar–H), 6.61 (s, 1H, Ar–H), 6.78 (s, 1H, Ar–H); ^13^C-NMR: (150 MHz, CDCl_3_) *δ* 14.23 (2 × C), 22.78 (2 × C), 27.39 (2 × C), 27.57 (2 × C), 29.51 (2 × C), 29.66 (3 × C), 31.98 (2 × C), 33.76, 51.65, 54.23 (2 × C), 54.53, 56.10, 59.91, 60.34, 100.89, 105.68, 105.88, 108.49, 124.92, 127.89, 128.93, 131.39, 141.33, 146.01, 146.31 (2 × C), 150.27; ESI–MS (*m*/*z*): 579.4 [M + H]^+^.

*Tetrahydroberberine-12-N,N-di-n-nonylamine* (**6i**). Target compound **6i** was obtained (303 mg, 88.5% yield) as a hazel oil from compound **5** (200 mg, 0.564 mmol), *n*-nonanal (330 μL, 1.92 mmol), sodium triacetoxyborohydride (478 mg, 2.256 mmol), and HOAc (13 drops) using a procedure similar to that for **6a**. ^1^H-NMR (400 MHz, CDCl_3_): *δ* 0.86 (t, *J* = 6.8 Hz, 6H, N(CH_2_CH_2_(CH_2_)_6_CH_3_)_2_), 1.23 (m, 24H), 1.39 (m, 4H), 2.46-2.68 (m, 3H), 2.85 (t, *J* = 7.2 Hz, 4H, N(CH_2_CH_2_(CH_2_)_6_CH_3_)_2_), 3.12–3.20 (m, 2H), 3.39–3.55 (m, 3H), 3.82 (s, 3H, OCH_3_), 3.83 (s, 3H, OCH_3_), 4.23 (d, *J* = 15.6 Hz, 1H), 5.92 (s, 2H, OCH_2_O), 6.60 (s, 1H, Ar-H), 6.61 (s, 1H, Ar-H), 6.79 (s, 1H, Ar-H); ^13^C NMR: (100 MHz, CDCl_3_) *δ* 14.25 (2 × C), 22.80 (2 × C), 27.38 (2 × C), 27.57 (2 × C), 29.43 (2 × C), 29.71 (3 × C), 29.81 (2 × C), 32.00 (2 × C), 33.75, 51.65, 54.22 (2 × C), 54.53, 56.08, 59.90, 60.32, 100.87, 105.65, 105.88, 108.48, 124.91, 127.89, 128.91, 131.38, 141.32, 146.00, 146.29, 146.30, 150.25; ESI-MS (*m*/*z*): 607.5 [M + H]^+^.

*Tetrahydroberberine-12-N,N-di-n-decylamine* (**6j**). Target compound **6j** was obtained (323 mg, 90% yield) as a hazel oil from compound **5** (200 mg, 0.564 mmol), *n*-decanal (362 μL, 1.92 mmol), sodium triacetoxyborohydride (478 mg, 2.256 mmol), and HOAc (13 drops) using a procedure similar to that for **6a**. ^1^H-NMR (400 MHz, CDCl_3_): *δ* 0.87 (t, *J* = 6.8 Hz, 6H, N(CH_2_CH_2_(CH_2_)_7_CH_3_)_2_), 1.23 (m, 28H), 1.39 (m, 4H), 2.46-2.68 (m, 3H), 2.85 (t, *J* = 7.2 Hz, 4H, N(CH_2_CH_2_(CH_2_)_7_CH_3_)_2_), 3.12-3.20 (m, 2H), 3.39-3.55 (m, 3H), 3.82 (s, 3H, OCH_3_), 3.83 (s, 3H, OCH_3_), 4.23 (d, *J* = 16 Hz, 1H), 5.92 (s, 2H, OCH_2_O), 6.60 (s, 1H, Ar–H), 6.61 (s, 1H, Ar–H), 6.79 (s, 1H, Ar–H); ^13^C-NMR: (100 MHz, CDCl_3_) *δ* 14.25 (2 × C), 22.82 (2 × C), 27.38 (2 × C), 27.58 (2 × C), 29.46 (2 × C), 29.72 (3 × C), 29.73 (2 × C), 29.87 (2 × C), 32.04 (2 × C), 33.77, 51.66, 54.21 (2 × C), 54.55, 56.08, 59.91, 60.32, 100.87, 105.65, 105.88, 108.49, 124.92, 127.90, 128.93, 131.40, 141.32, 145.99, 146.30 (2 × C), 150.25; ESI–MS (*m*/*z*): 635.5 [M + H]^+^.

*Tetrahydroberberine-12-N,N-di-n-undecylamine* (**6k**). Target compound **6k** was obtained (344 mg, 92% yield) as a hazel oil from compound **5** (200 mg, 0.564 mmol), *n*-undecaldehyde (393 μL, 1.92 mmol), sodium triacetoxyborohydride (478 mg, 2.256 mmol), and HOAc (13 drops) using a procedure similar to that for **6a**. ^1^H-NMR (400 MHz, CDCl_3_): *δ* 0.87 (t, *J* = 6.8 Hz, 6H, N(CH_2_CH_2_(CH_2_)_8_CH_3_)_2_), 1.23 (m, 32H), 1.39 (m, 4H), 2.45-2.68 (m, 3H), 2.85 (t, *J* = 7.2 Hz, 4H, N(CH_2_CH_2_(CH_2_)_8_CH_3_)_2_), 3.12–3.20 (m, 2H), 3.38–3.55 (m, 3H), 3.82 (s, 3H, OCH_3_), 3.83 (s, 3H, OCH_3_), 4.23 (d, *J* = 16 Hz, 1H), 5.92 (s, 2H, OCH_2_O), 6.60 (s, 1H, Ar-H), 6.61 (s, 1H, Ar-H), 6.78 (s, 1H, Ar-H); ^13^C–NMR: (100 MHz, CDCl_3_) *δ* 14.27 (2 × C), 22.83 (2 × C), 27.39 (2 × C), 27.59 (2 × C), 29.49 (2 × C), 29.73 (3 × C) 29.76 (2 × C), 29.79 (2 × C), 29.87 (2 × C), 32.06 (2 × C), 33.77, 51.67, 54.22 (2 × C), 54.55, 56.09, 59.92, 60.34, 100.88, 105.66, 105.89, 108.50, 124.93, 127.90, 128.95, 131.41, 141.33, 145.99, 146.30 (2 × C), 150.27; ESI–MS (*m*/*z*): 663.6 [M + H]^+^.

*Tetrahydroberberine-12-N,N-di-n-dodecylamine* (**6l**). Target compound **6l** was obtained (330 mg, 84.7% yield) as a hazel oil from compound **5** (200 mg, 0.564 mmol), *n*-dodecaldehyde (300 μL, 1.35 mmol), sodium triacetoxyborohydride (358 mg, 1.69 mmol), and HOAc (10 drops) using a procedure similar to that for **6a**. ^1^H NMR (400 MHz, CDCl_3_): *δ* 0.87 (t, *J* = 6.8 Hz, 6H, N(CH_2_CH_2_(CH_2_)_9_CH_3_)_2_), 1.23 (m, 36H), 1.40 (m, 4H), 2.45–2.68 (m, 3H), 2.85 (t, *J* = 7.2 Hz, 4H, N(CH_2_CH_2_(CH_2_)_9_CH_3_)_2_), 3.11–3.21 (m, 2H), 3.39–3.55 (m, 3H), 3.82 (s, 3H, OCH_3_), 3.83 (s, 3H, OCH_3_), 4.23 (d, *J* = 15.6 Hz,1H), 5.92 (s, 2H, OCH_2_O), 6.60 (s, 1H, Ar–H), 6.61 (s, 1H, Ar–H), 6.78 (s, 1H, Ar–H); ^13^C-NMR: (100 MHz, CDCl_3_) *δ* 14.27 (2 × C), 22.83 (2 × C), 27.39 (2 × C), 27.59 (2 × C), 29.51 (2 × C), 29.72 (3 × C), 29.79 (6 × C), 29.88 (2 × C), 32.06 (2 × C), 33.73, 51.63, 54.22 (2 × C), 54.52, 56.09, 59.90, 60.33, 100.88, 105.66, 105.89, 108.50, 124.91, 127.89, 128.90, 131.37, 141.28, 146.00, 146.30 (2 × C), 150.26; ESI–MS (*m*/*z*): 691.6 [M + H]^+^.

*Quaternary berberine-12-N,N-dimethylamine chloride* (**7a**). DDQ (220 mg) was weighed and dissolved in 8 mL of CH_2_Cl_2._ The DDQ solution was added dropwise into a solution containing compound **6a** (185 mg, 0.484 mmol) in CH_2_Cl_2_ (4 mL) under stirring. The reaction solution was stirred for 2h at room temperature until the raw material was completely reacted. The reaction mixture was concentrated to remove the solvent under reduced pressure, then aqueous 10% HCl solution (8 mL) was added into the residue. After stirring the mixture for 2h at room temperature, aqueous 1 N NaOH solution was added to make the solution alkaline. Then, the mixture was stirred for 0.5 h at room temperature and extracted using a mixed solution of CHCl_3_/CH_3_OH (*v*/*v* = 10:1) in a separatory funnel. The organic layer was washed, first, three times using water, then, one time using saturated aqueous NaCl solution. The organic layer was dried using anhydrous MgSO_4_ and filtered. The filtrate was concentrated under reduced pressure to remove the solvent. The residue was purified using silica gel CC eluted using a mixed solution of CHCl_3_/MeOH (*v*/*v* = 25:1) to yield **7a** (38 mg, 19% yield) as a red solid. ^1^H-NMR (400 MHz, DMSO-*d*_6_): *δ* 2.95 (s, 6H, N(CH_3_)_2_), 3.20 (t, *J* = 6 Hz, 2H, ArCH_2_CH_2_N), 4.00 (s, 3H, OCH_3_), 4.09 (s, 3H, OCH_3_), 4.92 (t, *J* = 6 Hz, 2H, ArCH_2_CH_2_N), 6.17 (s, 2H, OCH_2_O), 7.09 (s, 1H, Ar-H), 7.56 (s, 1H, Ar–H), 7.87 (s, 1H, Ar–H), 8.59 (s, 1H, Ar–H), 9.81 (s, 1H, Ar–H); ^13^C-NMR (100 MHz, DMSO-*d*_6_): *δ* 26.40, 44.83 (2 × C), 55.10, 56.95, 61.90, 102.05, 105.82, 108.40, 114.15, 116.65, 120.56, 122.14, 127.61, 130.71, 136.75, 138.24, 145.49, 147.57, 147.76, 149.77, 150.89; ESI–MS (*m*/*z*): 379.3 [M − Cl]^+^.

*Quaternary berberine-12-N,N-diethylamine chloride* (**7b**). Target compound **7b** was obtained (32 mg, 11.6% yield) as a red solid from compound **6b** (257 mg, 0.626 mmol) and DDQ (285 mg DDQ in 8 mL CH_2_Cl_2_) using a procedure similar to that for **7a**. ^1^H-NMR (400 MHz, DMSO-*d*_6_): *δ* 1.02 (t, *J* = 7.2 Hz, 6H, N(CH_2_CH_3_)_2_), 3.20 (t, *J* = 6 Hz, 2H, ArCH_2_CH_2_N), 3.27 (q, *J* = 7.2 Hz, 4H, N(CH_2_CH_3_)_2_), 4.04 (s, 3H, OCH_3_), 4.07 (s, 3H, OCH_3_), 4.92 (t, *J* = 6 Hz, 2H, ArCH_2_CH_2_N), 6.18 (s, 2H, OCH_2_O), 7.10 (s, 1H, Ar–H), 7.72 (s, 1H, Ar–H), 7.76 (s, 1H, Ar–H), 8.60 (s, 1H, Ar–H), 9.84 (s, 1H, Ar–H); ^13^C-NMR: (100 MHz, DMSO-*d*_6_) *δ* 12.04 (2 × C), 26.39, 47.68 (2 × C), 55.08, 57.15, 61.93, 102.09, 105.51, 108.46, 116.25, 118.78, 120.56, 122.02, 130.41, 130.85, 137.02, 139.37, 144.48, 145.59, 147.79, 149.82, 150.79; ESI–MS (*m*/*z*): 407.3 [M − Cl]^+^.

*Quaternary berberine-12-N,N-di-n-propylamine chloride* (**7c**). Target compound **7c** was obtained (50 mg, 46.6% yield) as a red solid from compound **6c** (100 mg, 0.228 mmol) and DDQ (103 mg DDQ in 6 mL CH_2_Cl_2_) using a procedure similar to that for **7a.** ^1^H-NMR (400 MHz, DMSO-*d*_6_): *δ* 0.85 (t, *J* = 7.2 Hz, 6H, N(CH_2_CH_2_CH_3_)_2_), 1.47 (m, 4H, N(CH_2_CH_2_CH_3_)_2_), 3.19 (m, 6H, ArCH_2_CH_2_N, N(CH_2_CH_2_CH_3_)_2_), 4.03 (s, 3H, OCH_3_), 4.07 (s, 3H, OCH_3_), 4.92 (t, *J* = 6 Hz, 2H, ArCH_2_CH_2_N), 6.18 (s, 2H, OCH_2_O), 7.10 (s, 1H, Ar–H), 7.65 (s, 1H, Ar–H), 7.78 (s, 1H, Ar–H), 8.61 (s, 1H, Ar–H), 9.84 (s, 1H, Ar–H); ^13^C-NMR: (150 MHz, DMSO-*d*_6_) *δ* 11.62 (2 × C), 19.83 (2 × C), 26.38, 55.06, 55.75 (2 × C), 57.17, 61.93, 102.12, 105.11, 108.52, 115.94, 119.14, 120.50, 121.97, 130.25, 130.87, 136.97, 139.53, 145.08, 145.71, 147.78, 149.85, 150.85; ESI–MS (*m*/*z*): 435.3 [M − Cl]^+^.

*Quaternary berberine-12-N,N-di-n-butylamine chloride* (**7d**). Target compound **7d** was obtained (100 mg, 46.7% yield) as a red solid from compound **6d** (200 mg, 0.429 mmol) and DDQ (195 mg DDQ in 8 mL CH_2_Cl_2_) using a procedure similar to that for **7a**. ^1^H-NMR (400 MHz, DMSO-*d*_6_): *δ* 0.84 (t, *J* = 7.2 Hz, 6H, N(CH_2_CH_2_CH_2_CH_3_)_2)_, 1.28(m, 4H, N(CH_2_CH_2_CH_2_CH_3_)_2_), 1.45 (m, 4H, N(CH_2_CH_2_CH_2_CH_3_)_2_), 3.21 (m, 6H, ArCH_2_CH_2_N, N(CH_2_CH_2_CH_2_CH_3_)_2_), 4.03 (s, 3H, OCH_3_), 4.06 (s, 3H, OCH_3_), 4.92 (t, *J* = 6 Hz, 2H, ArCH_2_CH_2_N), 6.19 (s, 2H, OCH_2_O), 7.10 (s, 1H, Ar–H), 7.63 (s, 1H, Ar–H), 7.76 (s, 1H, Ar–H), 8.57 (s, 1H, Ar–H), 9.84 (s, 1H, Ar–H); ^13^C-NMR: (100 MHz, DMSO-*d*_6_) *δ* 13.86 (2 × C), 19.93 (2 × C), 26.37, 28.74 (2 × C), 53.65 (2 × C), 55.06, 57.18, 61.95, 102.14, 105.09, 108.55, 115.89, 118.86, 120.50, 121.99, 130.14, 130.88, 136.95, 139.43, 145.15, 145.76, 147.79, 149.86, 150.84; ESI–MS (*m*/*z*): 463.3 [M − Cl]^+^.

*Quaternary berberine-12-N,N-di-n-pentylamine chloride* (**7e**). Target compound **7e** was obtained (70 mg, 11.4% yield) as a red solid from compound **6e** (273 mg, 0.552 mmol) and DDQ (251 mg DDQ in 10 mL CH_2_Cl_2_) using a procedure similar to that for **7a**. ^1^H-NMR (400 MHz, DMSO-*d*_6_): *δ* 0.82 (t, *J* = 6.4 Hz, 6H, N(CH_2_CH_2_CH_2_CH_2_CH_3_)_2_), 1.26(m, 8H, N(CH_2_CH_2_CH_2_CH_2_CH_3_)_2_), 1.47 (m, 4H, N(CH_2_CH_2_CH_2_CH_2_CH_3_)_2_), 3.20 (m, 6H, ArCH_2_CH_2_N, N(CH_2_CH_2_CH_2_CH_2_CH_3_)_2_), 4.04 (s, 3H, OCH_3_), 4.06 (s, 3H, OCH_3_), 4.92 (t, *J* = 6 Hz, 2H, ArCH_2_CH_2_N), 6.18 (s, 2H, OCH_2_O), 7.11 (s, 1H, Ar–H), 7.60 (s, 1H, Ar–H), 7.78 (s, 1H, Ar–H), 8.59 (s, 1H, Ar–H), 9.84 (s, 1H, Ar–H); ^13^C-NMR: (100 MHz, DMSO-*d*_6_) *δ* 13.99 (2 × C), 21.96 (2 × C), 26.31 (2 × C), 26.37, 29.03 (2 × C), 53.85 (2 × C), 55.04, 57.19, 61.95, 102.16, 104.96, 108.58, 115.87, 118.95, 120.50, 121.99, 130.19, 130.88, 136.94, 139.49, 145.22, 145.77, 147.80, 149.87, 150.86; ESI–MS (*m*/*z*): 491.3 [M − Cl]^+^.

*Quaternary berberine-12-N,N-di-n-hexylamine chloride* (**7f**). Target compound **7f** was obtained (52 mg, 29.4% yield) as a red solid from compound **6f** (167 mg, 0.32 mmol) and DDQ (145 mg DDQ in 6 mL CH_2_Cl_2_) using a procedure similar to that for **7a**. ^1^H-NMR (400 MHz, DMSO-*d*_6_): *δ* 0.81 (t, *J* = 6.4 Hz, 6H, N(CH_2_CH_2_CH_2_CH_2_CH_2_CH_3_)_2_), 1.22(m, 12H, N(CH_2_CH_2_CH_2_CH_2_CH_2_CH_3_)_2_), 1.47 (m, 4H, N(CH_2_CH_2_CH_2_CH_2_CH_2_CH_3_)_2_), 3.20 (m, 6H, ArCH_2_CH_2_N, N(CH_2_CH_2_CH_2_CH_2_CH_2_CH_3_)_2_), 4.04 (s, 3H, OCH_3_), 4.06 (s, 3H, OCH_3_), 4.92 (t, *J* = 6 Hz, 2H, ArCH_2_CH_2_N), 6.18 (s, 2H, OCH_2_O), 7.11 (s, 1H, Ar–H), 7.59 (s, 1H, Ar–H), 7.78 (s, 1H, Ar–H), 8.58 (s, 1H, Ar–H), 9.85 (s, 1H, Ar–H); ^13^C-NMR: (100 MHz, DMSO-*d*_6_) *δ* 13.84 (2 × C), 22.13 (2 × C), 26.37, 26.43 (2 × C), 26.60 (2 × C), 31.05 (2 × C), 53.85 (2 × C), 55.04, 57.18, 61.94, 102.17, 104.93, 108.58, 115.87, 118.97, 120.49, 121.98, 130.21, 130.88, 136.93, 139.50, 145.27, 145.76, 147.80, 149.86, 150.87; ESI–MS (*m*/*z*): 519.4 [M − Cl]^+^.

*Quaternary berberine-12-N,N-di-n-heptylamine chloride* (**7g**). Target compound **7g** was obtained (50 mg, 22.7% yield) as a red solid from compound **6g** (208 mg, 0.378 mmol) and DDQ (172 mg DDQ in 8 mL CH_2_Cl_2_) using a procedure similar to that for **7a**. ^1^H-NMR (400 MHz, DMSO-*d*_6_): *δ* 0.80 (m, 6H, N(CH_2_CH_2_(CH_2_)_4_CH_3_)_2_), 1.20 (m, 16H, ArN(CH_2_CH_2_(CH_2_)_4_CH_3_)_2_), 1.47 (m, 4H, N(CH_2_CH_2_(CH_2_)_4_CH_3_)_2_), 3.19 (m, 6H, ArCH_2_CH_2_N, N(CH_2_CH_2_(CH_2_)_4_CH_3_)_2_), 4.04 (s, 3H, OCH_3_), 4.07 (s, 3H, OCH_3_), 4.93 (t, *J* = 6 Hz, 2H, ArCH_2_CH_2_N), 6.18 (s, 2H, OCH_2_O), 7.12 (s, 1H, Ar–H), 7.58 (s, 1H, Ar–H), 7.79 (s, 1H, Ar-H), 8.59 (s, 1H, Ar-H), 9.87 (s, 1H, Ar-H); ^13^C-NMR: (100 MHz, DMSO-*d*_6_) *δ* 13.88(2 × C), 22.02 (2 × C), 26.38, 26.65 (2 × C), 26.73 (2 × C), 28.49 (2 × C), 31.32 (2 × C), 53.83 (2 × C), 55.04, 57.18, 61.95, 102.16, 104.87, 108.59, 115.84, 119.09, 120.49, 121.96, 130.26, 130.89, 136.91, 139.57, 145.28, 145.80, 147.80, 149.86, 150.89; ESI–MS (*m*/*z*): 547.4 [M − Cl]^+^.

*Quaternary berberine-12-N,N-di-n-octylamine chloride* (**7h**). Target compound **7h** was obtained (70 mg, 30% yield) as a red solid from compound **6h** (220 mg, 0.380 mmol) and DDQ (173 mg DDQ in 8 mL CH_2_Cl_2_) using a procedure similar to that for **7a**. ^1^H-NMR (400 MHz, DMSO-*d*_6_): *δ* 0.80 (t, *J* = 6.4 Hz, 6H, N(CH_2_CH_2_(CH_2_)_5_CH_3_)_2_), 1.20 (m, 20H, N(CH_2_CH_2_(CH_2_)_5_CH_3_)_2_), 1.46 (m, 4H, N(CH_2_CH_2_(CH_2_)_5_CH_3_)_2_), 3.19 (m, 6H, ArCH_2_CH_2_N, N(CH_2_CH_2_(CH_2_)_5_CH_3_)_2_), 4.03 (s, 3H, OCH_3_), 4.06 (s, 3H, OCH_3_), 4.92 (t, *J* = 6 Hz, 2H, ArCH_2_CH_2_N), 6.17 (s, 2H, OCH_2_O), 7.11 (s, 1H, Ar–H), 7.58 (s, 1H, Ar–H), 7.78 (s, 1H, Ar–H), 8.58 (s, 1H, Ar–H), 9.84 (s, 1H, Ar–H); ^13^C-NMR: (100 MHz, DMSO-*d*_6_) *δ* 13.87 (2 × C), 22.04 (2 × C), 26.38, 26.62 (2 × C), 26.76 (2 × C), 28.74 (2 × C), 28.77 (2 × C), 31.19 (2 × C), 53.79(2 × C), 55.05, 57.17, 61.94, 102.16, 104.87, 108.59, 115.84, 119.06, 120.49, 121.97, 130.24, 130.88, 136.91, 139.55, 145.29, 145.80, 147.81, 149.86, 150.89; ESI–MS (*m*/*z*): 575.5 [M − Cl]^+^.

*Quaternary berberine-12-N,N-di-n-nonylamine chloride* (**7i**). Target compound **7i** was obtained (87 mg, 34.4% yield) as a red solid from compound **6i** (240 mg, 0.396 mmol) and DDQ (180 mg DDQ in 6 mL CH_2_Cl_2_) using a procedure similar to that for **7a**. ^1^H-NMR (400 MHz, DMSO-*d*_6_): *δ* 0.81 (t, *J* = 6.4 Hz, 6H, N(CH_2_CH_2_(CH_2_)_6_CH_3_)_2_), 1.18 (m, 24H, N(CH_2_CH_2_(CH_2_)_6_CH_3_)_2_), 1.46 (m, 4H, N(CH_2_CH_2_(CH_2_)_6_CH_3_)_2_), 3.18 (m, 6H, ArCH_2_CH_2_N, N(CH_2_CH_2_(CH_2_)_6_CH_3_)_2_), 4.04 (s, 3H, OCH_3_), 4.06 (s, 3H, OCH_3_), 4.92 (t, *J* = 5.6 Hz, 2H, ArCH_2_CH_2_N), 6.17 (s, 2H, OCH_2_O), 7.11 (s, 1H, Ar–H), 7.57 (s, 1H, Ar–H), 7.79 (s, 1H, Ar–H), 8.58 (s, 1H, Ar–H), 9.85 (s, 1H, Ar–H); ^13^C-NMR: (100 MHz, DMSO-*d*_6_) *δ* 13.91 (2 × C), 22.03 (2 × C), 26.38, 26.62 (2 × C), 26.75 (2 × C), 28.61 (2 × C), 28.81 (2 × C), 29.03 (2 × C), 31.23 (2 × C), 53.78 (2 × C), 55.05, 57.17, 61.94, 102.15, 104.85, 108.60, 115.82, 119.09, 120.48, 121.97, 130.24, 130.88, 136.90, 139.57, 145.30, 145.81, 147.81, 149.86, 150.89; ESI–MS (*m*/*z*): 603.5 [M − Cl]^+^.

*Quaternary berberine-12-N,N-di-n-decylamine chloride* (**7j**). Target compound **7j** was obtained (47 mg, 14.2% yield) as a red solid from compound **6j** (315 mg, 0.496 mmol) and DDQ (225 mg DDQ in 8 mL CH_2_Cl_2_) using a procedure similar to that for **7a**. ^1^H-NMR (400 MHz, DMSO-*d*_6_): *δ* 0.82 (t, *J* = 6.4 Hz, 6H, N(CH_2_CH_2_(CH_2_)_7_CH_3_)_2_), 1.18 (m, 28H, N(CH_2_CH_2_(CH_2_)_7_CH_3_)_2_), 1.46 (m, 4H, N(CH_2_CH_2_(CH_2_)_7_CH_3_)_2_), 3.18 (m, 6H, ArCH_2_CH_2_N, N(CH_2_CH_2_(CH_2_)_7_CH_3_)_2_), 4.04 (s, 3H, OCH_3_), 4.06 (s, 3H, OCH_3_), 4.92 (t, *J* = 6 Hz, 2H, ArCH_2_CH_2_N), 6.17 (s, 2H, OCH_2_O), 7.11 (s, 1H, Ar-H), 7.57 (s, 1H, Ar–H), 7.79 (s, 1H, Ar–H), 8.58 (s, 1H, Ar–H), 9.85 (s, 1H, Ar–H); ^13^C-NMR: (100 MHz, DMSO-*d*_6_) *δ* 13.92 (2 × C), 22.07 (2 × C), 26.37, 26.61 (2 × C), 26.74 (2 × C), 28.66 (2 × C), 28.79 (2 × C), 28.91 (2 × C), 29.08 (2 × C), 31.23 (2 × C), 53.76 (2 × C), 55.05, 57.16, 61.93, 102.14, 104.84, 108.60, 115.81, 119.08, 120.48, 121.96, 130.23, 130.87, 136.89, 139.55, 145.30, 145.80, 147.80, 149.85, 150.88; ESI–MS (*m*/*z*): 631.5 [M − Cl]^+^.

*Quaternary berberine-12-N,N-di-n-undecylamine chloride* (**7k**). Target compound **7k** was obtained (55 mg, 16% yield) as a red solid from compound **6k** (328 mg, 0.495 mmol) and DDQ (225 mg DDQ in 8 mL CH_2_Cl_2_) using a procedure similar to that for **7a**. ^1^H-NMR (400 MHz, DMSO-*d*_6_): *δ* 0.83 (t, *J* = 6.4 Hz, 6H, N(CH_2_CH_2_(CH_2_)_8_CH_3_)_2_), 1.18 (m, 32H, N(CH_2_CH_2_(CH_2_)_8_CH_3_)_2_), 1.46 (m, 4H, N(CH_2_CH_2_(CH_2_)_8_CH_3_)_2_), 3.18 (m, 6H, ArCH_2_CH_2_N, N(CH_2_CH_2_(CH_2_)_8_CH_3_)_2_), 4.04 (s, 3H, OCH_3_), 4.06 (s, 3H, OCH_3_), 4.92 (t, *J* = 5.6 Hz, 2H, ArCH_2_CH_2_N), 6.17 (s, 2H, OCH_2_O), 7.11 (s, 1H, Ar–H), 7.57 (s, 1H, Ar–H), 7.79 (s, 1H, Ar–H), 8.58 (s, 1H, Ar–H), 9.85 (s, 1H, Ar–H); ^13^C-NMR: (100 MHz, DMSO-*d*_6_) *δ* 13.93 (2 × C), 22.07 (2 × C), 26.37, 26.60 (2 × C), 26.73 (2 × C), 28.66 (2 × C), 28.79 (2 × C), 28.96 (4×C), 29.07 (2 × C), 31.27 (2 × C), 53.74 (2 × C), 55.05, 57.16, 61.93, 102.14, 104.83, 108.60, 115.80, 119.08, 120.47, 121.96, 130.23, 130.87, 136.89, 139.56, 145.30, 145.81, 147.80, 149.85, 150.88; ESI–MS (*m*/*z*): 659.6 [M − Cl]^+^.

*Quaternary berberine-12-N,N-di-n-dodecylamine chloride* (**7l**). Target compound **7l** was obtained (100 mg, 38.3% yield) as a red solid from compound **6l** (250 mg, 0.362 mmol) and DDQ (165 mg DDQ in 8 mL CH_2_Cl_2_) using a procedure similar to that for **7a**. ^1^H-NMR (400 MHz, DMSO-*d*_6_): *δ* 0.84 (t, *J* = 6.4 Hz, 6H, N(CH_2_CH_2_(CH_2_)_9_CH_3_)_2_), 1.18 (m, 36H, N(CH_2_CH_2_(CH_2_)_9_CH_3_)_2_), 1.47 (m, 4H, N(CH_2_CH_2_(CH_2_)_9_CH_3_)_2_), 3.18 (m, 6H, ArCH_2_CH_2_N, N(CH_2_CH_2_(CH_2_)_9_CH_3_)_2_), 4.04 (s, 3H, OCH_3_), 4.06 (s, 3H, OCH_3_), 4.92 (t, *J* = 6 Hz, 2H, ArCH_2_CH_2_N), 6.17 (s, 2H, OCH_2_O), 7.11 (s, 1H, Ar–H), 7.56 (s, 1H, Ar–H), 7.79 (s, 1H, Ar–H), 8.58 (s, 1H, Ar–H), 9.84 (s, 1H, Ar–H); ^13^C-NMR: (100 MHz, DMSO-*d*_6_) *δ* 13.94 (2 × C), 22.09 (2 × C), 26.37, 26.60 (2 × C), 26.73 (2 × C), 28.70 (2 × C), 28.78 (2 × C), 28.96 (4×C), 29.01 (2 × C), 29.07 (2 × C), 31.28 (2 × C), 53.74 (2 × C), 55.05, 57.17, 61.94, 102.14, 104.81, 108.61, 115.79, 119.10, 120.47, 121.97, 130.22, 130.88, 136.89, 139.59, 145.31, 145.83, 147.80, 149.86, 150.89; ESI–MS (*m*/*z*): 687.6 [M − Cl]^+^.

*Tetrahydroberberine-12-N-n-propylamine derivatives* (**8**). To a stirred solution of compound **5** (200 mg, 0.564 mmol) in CH_2_Cl_2_ (4 mL), we added propionaldehyde (41 μL, 0.564 mmol), sodium sodium triacetoxyborohydride (143 mg, 0.677 mmol), and acetic acid (10 drops), respectively. The reaction solution was stirred for 2h at room temperature until the raw material was completely reacted. Then, aqueous saturated NaHCO_3_ solution was added dropwise to make the solution alkaline (pH = 8). The solution was extracted three times (20mL/time) using CH_2_Cl_2_ in a separatory funnel. The organic layer was washed, first, three times using water, then one time using aqueous saturated NaCl solution. The organic layer was dried using anhydrous MgSO_4_ and filtered. The filtrate was concentrated under reduced pressure to remove the solvent. The residue was purified using silica gel CC eluted using a mixed solution of CH_2_Cl_2_/MeOH (*v*/*v* = 80:1) to yield **8** (140 mg, 62.8% yield) as a light grey solid. ^1^H-NMR (400 MHz, CDCl_3_): *δ* 1.02 (t, *J* = 7.6 Hz, 3H, NHCH_2_CH_2_CH_3_), 1.69 (m, 2H), 2.34–3.58 (m, 10H), 3.77 (s, 3H, OCH_3_), 3.86 (s, 3H, OCH_3_), 4.20 (d, *J* = 15.2 Hz,1H), 5.93 (s, 2H, OCH_2_O), 6.17 (s, 1H, Ar–H), 6.60 (s, 1H, Ar–H), 6.77 (s, 1H, Ar–H); ESI–MS (*m*/*z*): 397.3 [M + H]^+^.

*Quaternary berberine-12-N-n-propylamine iodide* (**9**). To a stirred solution of compound 8 (130 mg, 0.328 mmol) in anhydrous ethanol (8 mL), we added iodine (250 mg, 0.984 mmol). After the reaction solution was refluxed for 10 h, a second batch of iodine (125 mg, 0.492 mmol) was added to the solution. The reaction solution was refluxed for another 10 h and a third batch of iodine (83 mg, 0.328 mmol) was added. After the reaction was refluxed for another 10 h, aqueous saturated sodium thiosulfate solution (2 mL) was added to quench the reaction. The reaction mixture was filtered. The filter cake was dissolved in a mixed solution of CHCl_3_/MeOH (*v*/*v* = 10:1). The organic layer was washed, first, three times using water, then, one time using aqueous saturated NaCl solution in a separatory funnel. Then, the organic layer was dried by anhydrous MgSO_4_ and filtered. The filtrate was concentrated under reduced pressure to remove the solvent. The residue was purified by silica gel CC eluted using a mixed solution of CH_2_Cl_2_/MeOH (*v*/*v* = 200:1) to yield **9** (35 mg, 15% yield) as a reddish brown solid. ^1^H-NMR (400 MHz, DMSO-*d*_6_): *δ* 1.04 (t, *J* = 7.2 Hz, 3H, NHCH_2_CH_2_CH_3_), 1.38 (m, 2H, NHCH_2_CH_2_CH_3_), 1.77 (m, 2H, NHCH_2_CH_2_CH_3_), 3.19 (t, *J* = 6 Hz, 2H, Ar-CH_2_CH_2_N), 3.90 (s, 3H, OCH_3_), 4.04 (s, 3H, OCH_3_), 4.88 (t, *J* = 6 Hz, 2H, Ar–CH_2_CH_2_N), 6.18 (s, 2H, OCH_2_O), 6.84 (s, 1H, Ar–H), 6.97 (t, *J* = 5.6 Hz, 1H, NHCH_2_CH_2_CH_3_), 7.08 (s, 1H, Ar–H), 7.88 (s, 1H, Ar–H), 8.87 (s, 1H, Ar–H), 9.61 (s, 1H, Ar–H); ESI–MS (*m*/*z*): 393.3 [M − I]^+^.

### 3.2. Growth Inhibition Activity Assay Against Human Cancer Cell Lines

The growth inhibitory activity of all the synthesized compounds against the human HCT-8, Bel7402, HeLa, A549, and BGC-823 cell lines were examined using a published method of our group [25]. The compounds were dissolved in DMSO (100 μL), and then the solutions containing the compounds were diluted to working solutions with RPMI 1640 culture medium containing 10% serum. The corresponding human tumor cells were added to 96-well microplates (100 μL/well), and cultured in an incubator with 5% CO_2_ at 37 °C for 24 h. The working solutions of the compounds were added to microplates at a final concentration of 0.1 μmol/L, 1 μmol/L, 10 μmol/L, and 100 μmol/L (4 replicate wells per concentration). After 72 h, the culture solution was discarded, and RPMI 1640 culture medium (10% serum, 100 μL) containing 0.5 mg/mL MTT was added to each well. After incubating at 37 °C, 5% CO_2_ for 4 h, the solution was discarded. DMSO (150 μL) was added to each well, and the plates were shaken at room temperature for 10 min to completely dissolve the blue crystals in order to detect the optical density (OD) value at 570 nm (detection wavelength) and 655 nm (reference wavelength) using a Bio-Rad 450 microplate reader (Hercules, CA, USA). The inhibition rate of the test compound was calculated according to the following formula:

Growth inhibition rate % = (negative control OD − pending compound OD)/(negative control OD − background OD) × 100%(1)

## 4. Conclusions

In this article, quaternary 12-nitroberberine chloride (**2**), quaternary 12-aminoberberine chloride (**3**), tertiary 12-aminotetrahydroberberine (**5**), and five series of 12-aminoberberine derivatives of different reduction states, including quaternary berberine-12-*N*-acylamine chlorides (**4a**–**m**), tertiary tetrahydroberberine-12-*N,N*-di-*n*-alkylamine derivatives (**6a**–**l**), quaternary berberine-12-*N,N*-di-*n*-alkylamine chlorides (**7a**–**l**), tertiary tetrahydroberberine-12-*N*-*n*-propylamine (**8**), and quaternary berberine-12-*N*-*n*-propylamine iodide (**9**) were designed and synthesized. The growth inhibition activities of these synthesized compounds against several human cancer cell lines were screened. The series of quaternary berberine-12-*N,N*-di-*n*-alkylamine chlorides (**7a**–**l**) showed some or significant growth inhibition activities against the tested human cancer cell lines, with the IC_50_ values of most compounds being on or over the micromolar level. In addition, significant SAR was observed. Firstly, the activities of quaternary berberine-12-*N,N*-di-*n*-alkylamine chlorides series (**7a**–**l**) are obviously stronger than those of the reduced counterparts, the tertiary tetrahydroberberine-12-*N,N*-di-*n*-alkylamine derivatives series (**6a**–**l**). Secondly, the length of the *n*-alkyl carbon chain of 12-*N,N*-di-*n*-alkylaminos has a significant effect on the activities. In the range of about six to eight carbon atoms, the activity increases with the elongation of the *n*-alkyl carbon chain of 12-*N,N*-di-*n*-alkylaminos, and when there are more than six to eight carbon atoms, the activity decreases with the elongation of the *n*-alkyl carbon chain. Regarding the activity feature of 12-*N,N*-di-*n*-alkylaminos, we guess that it is relevant to their log P values and their ability to cross cell membranes; this is an area of further research for us. Thirdly, the activities are also affected by the number of *n*-alkyl groups on the amino nitrogen atom. The activities of the tertiary amine structure of the 12-amino are significantly higher than the secondary amine structure. These findings are very helpful for the further medicinal chemistry study of berberine-type alkaloids.

## Figures and Tables

**Scheme 1:**
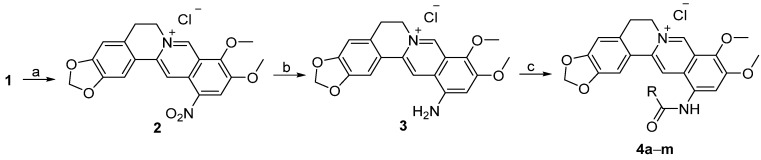
Syntheses of compounds **2**, **3**, and **4a–m**. Reagents and conditions: (a) NaNO_2_, concentrated HNO_3_, AcOH, 50°C, 1h; (b) SnCl_2_·2H_2_O, concentrated HCl, EtOH, reflux, 30min; (c) RCOCl, pyridine, CH_2_Cl_2_, room temperature (rt), 12h. R= **a**: *p*-trifluoromethylphenyl; **b**: Me; **c**: isopropyl; **d**: *p*-bromophenyl; **e**: phenyl; **f**: *p*-fluorophenyl; **g**: *p*-methylphenyl; **h**: *m*-methylphenyl; **i**: Et; **j**: *n*-Bu; **k**: *p*-methoxyphenyl; **l**: *p*-nitrophenyl; **m**: *t*-Bu.

**Scheme 2:**
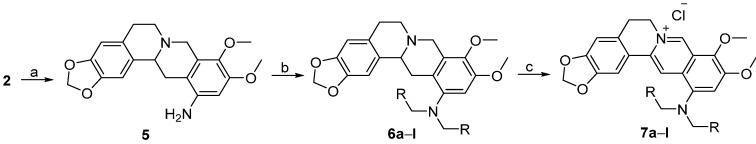
Syntheses of compounds **5**, **6a**–**l**, and **7a**–**l**. Reagents and conditions: (**a**) NaBH_4_, NiCl_2_·6H_2_O, THF/CH_3_OH, 66 °C, 20 min; (**b**) RCHO, NaBH(OCOCH_3_)_3_, AcOH, CH_2_Cl_2_, rt, 1h; (**c**) 2,3-dichloro-5,6-dicyano-1,4-benzoquinone (DDQ), CH_2_Cl_2_, 2N HCl, rt, 2h. R = **a**–**l:** C_n_H_2n + 1_ (n = 0–11).

**Table 1:** IC_50_ values of the active compounds inhibiting human cancer cell growth (μM).

Compounds	HCT-8	Bel7402	HeLa	A549	BGC-823
Fluorouracil (5-FU)	8.00	5.61	7.84	6.49	7.15
QBC	35.16	26.26	7.03	20.70	19.20
**2**	50.36	31.41	17.29	50.41	27.89
**3**	- ^a^	- ^a^	56.32	41.10	90.57
**5**	94.63	57.29	62.33	91.33	- ^a^
**6a**	34.79	34.39	31.09	55.96	38.19
**6b**	32.68	41.98	26.37	35.47	60.62
**6c**	29.68	32.02	18.72	31.21	22.53
**6d**	12.81	16.38	9.74	11.40	10.43
**6e**	17.28	22.92	14.54	19.03	12.34
**6f**	18.07	28.78	13.09	14.73	18.29
**6g**	26.80	54.00	26.43	19.16	17.88
**6h**	26.22	42.76	26.80	17.31	18.54
**6i**	- ^a^	- ^a^	62.78	73.38	59.13
**6j**	- ^a^	- ^a^	44.77	47.17	81.70
**7a**	53.41	50.01	8.95	13.37	28.82
**7b**	14.29	28.34	2.14	7.14	7.83
**7c**	5.63	6.03	1.57	5.26	6.07
**7d**	2.21	2.57	0.53	0.54	1.04
**7e**	0.92	1.30	0.82	0.90	0.75
**7f**	0.95	0.80	0.36	0.29	0.94
**7g**	0.49	0.51	0.26	- ^a^	0.35
**7h**	1.40	3.21	0.55	0.46	0.11
**7i**	2.15	2.59	0.95	0.66	0.28
**7j**	9.51	16.21	2.14	1.29	1.42
**7k**	20.71	18.90	10.44	7.87	8.18
**7l**	31.48	23.40	27.30	26.32	20.89

^a^ Data not available.

## Supplementary Materials

The following are available online, Figures S1–S81: NMR spectra of all the synthesized compounds.
