# Supplementary material for: Syntheses and Structure–Activity Relationships in Growth Inhibition Activity against Human Cancer Cell Lines of 12 Substituted Berberine Derivatives
Source: Molecules. 2020 Apr 18;25(8):1871. doi: 10.3390/molecules25081871 (PMC7221678; doi:10.3390/molecules25081871)
Supplement: Supplementary file 1 [file molecules-25-01871-s001.pdf]

Supplementary Materials: Syntheses and Structure–Activity Relationships in Growth Inhibition Activity against Human Cancer Cell Lines of 12 Substituted Berberine Derivatives

Bo Wang, An-Jun Deng *, Zhi-Hong Li, Nan Wang and Hai-Lin Qin *

State Key Laboratory of Bioactive Substance and Function of Natural Medicines, Institute of Materia Medica, Chinese Academy of Medical Sciences and Peking Union Medical College, Beijing 100050, China; wangbping@yeah.net (B.W.); zhl@imm.ac.cn (Z.-H.L.); wangnan@imm.ac.cn (N.W.)

* Correspondences: denganjun@imm.ac.cn (A.-J.D.); qinhailin@imm.ac.cn (H.-L. Q.)

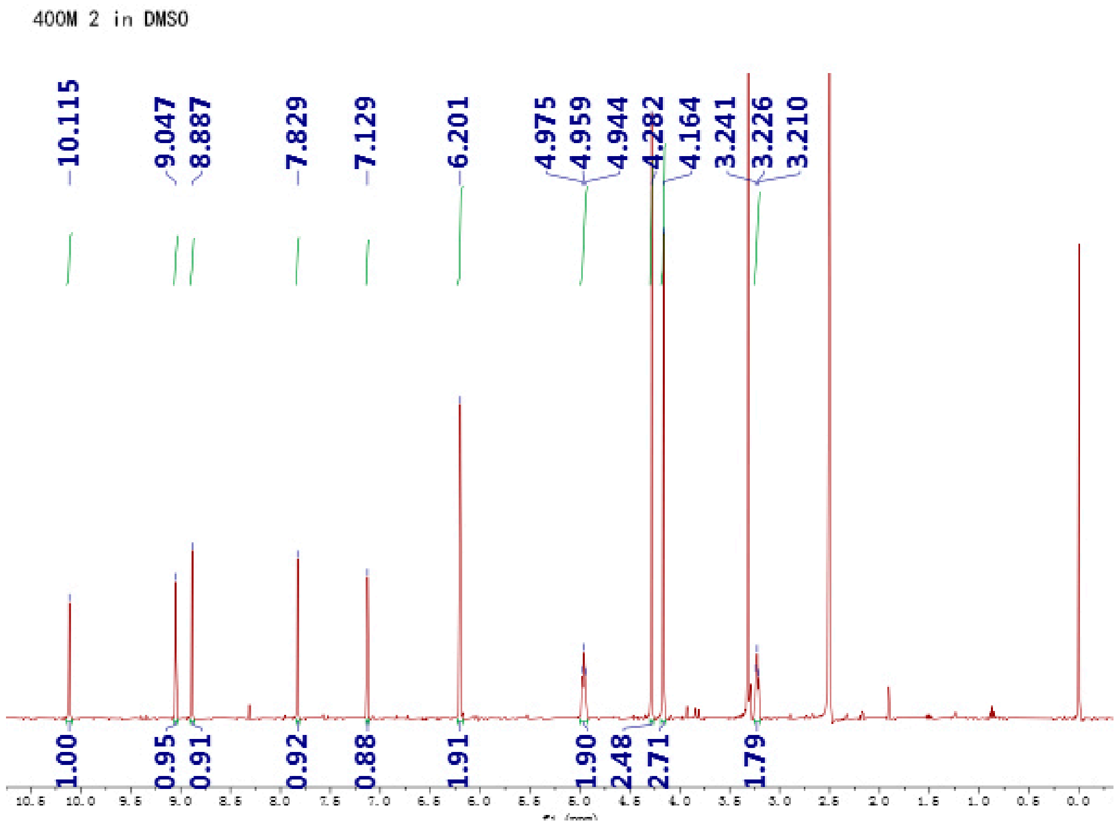

Figure S1. ^{1}H-NMR spectrum of compound **2**

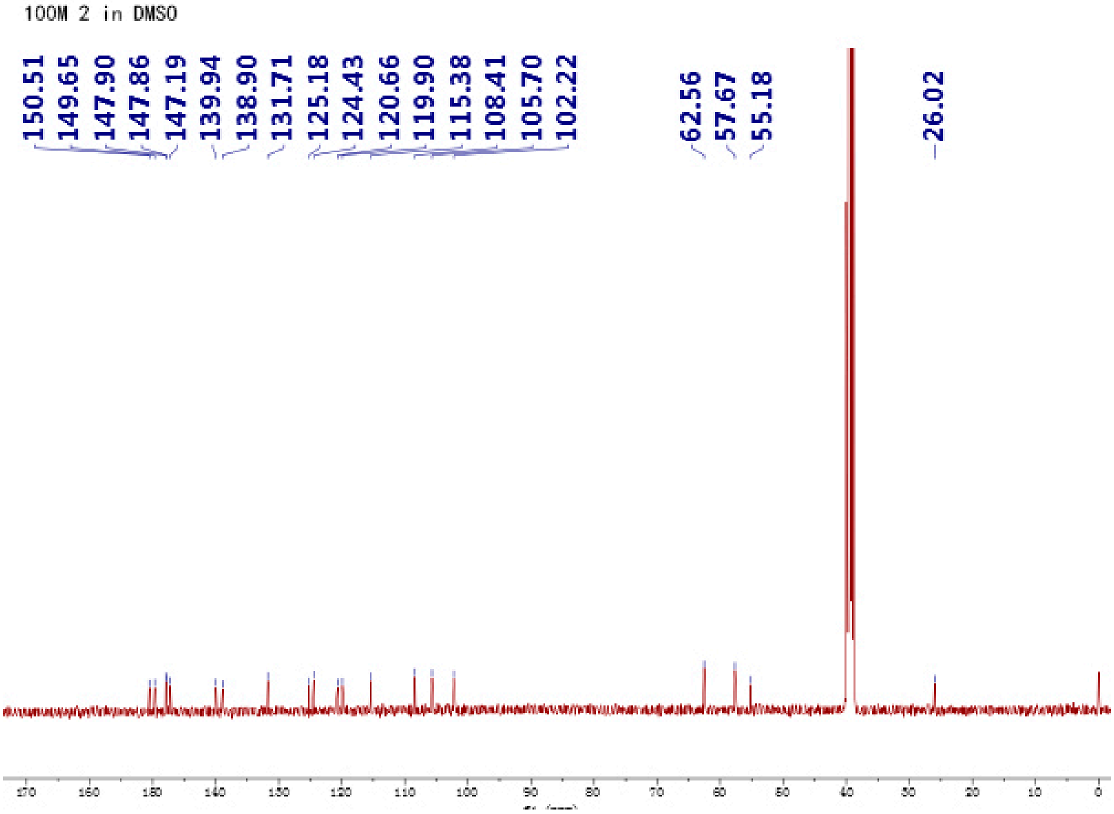

Figure S2. ^{13}C-NMR spectrum of compound **2**

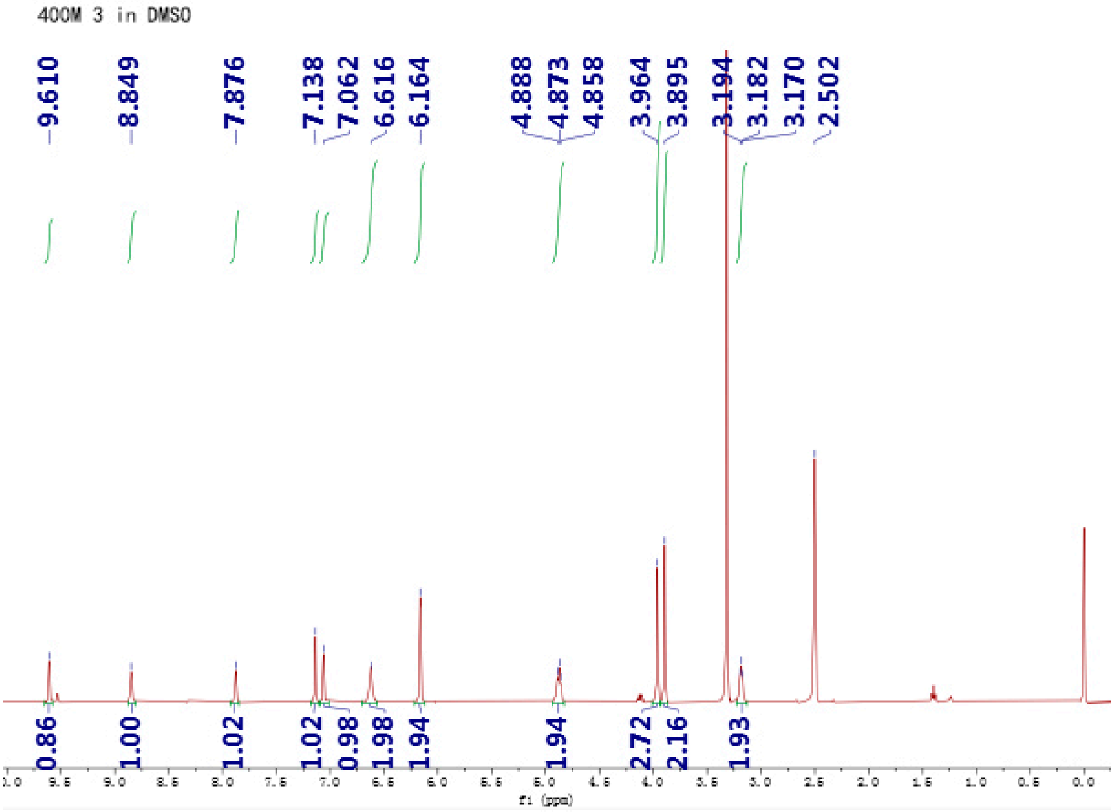

Figure S3. ^{1}H-NMR spectrum of compound **3**

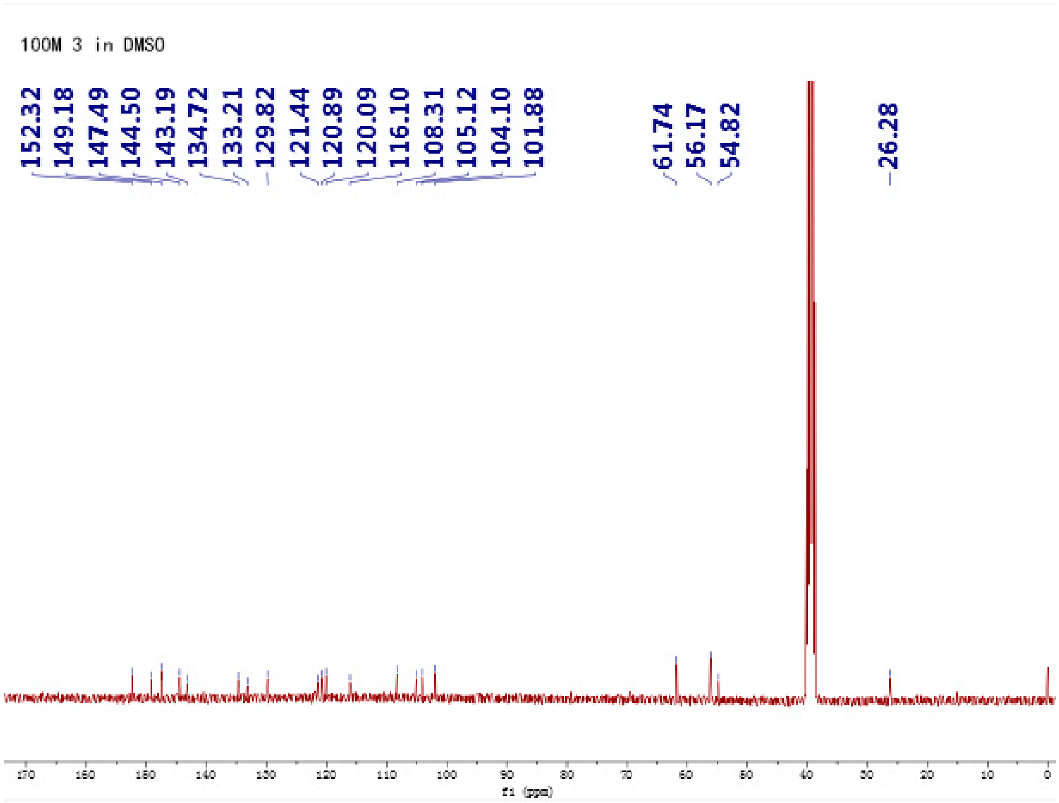

Figure S4. ^{13}C-NMR spectrum of compound **3**

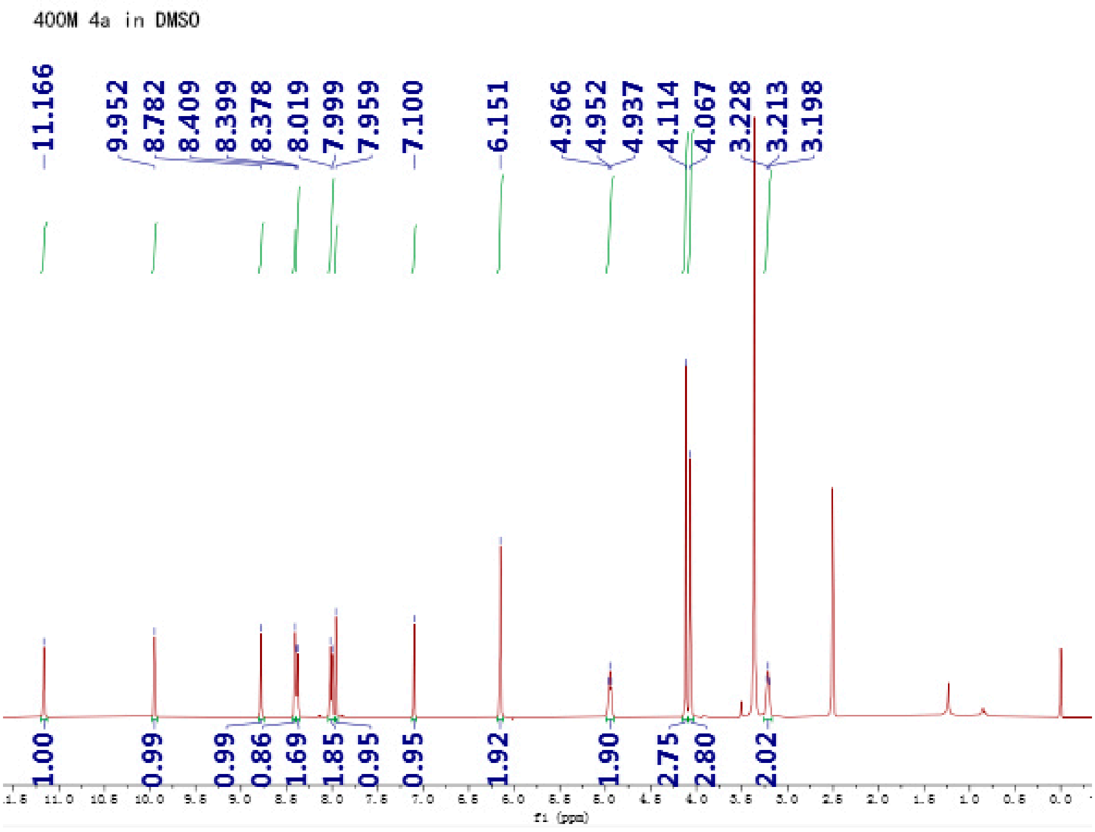

Figure S5. ^{1}H-NMR spectrum of compound **4a**

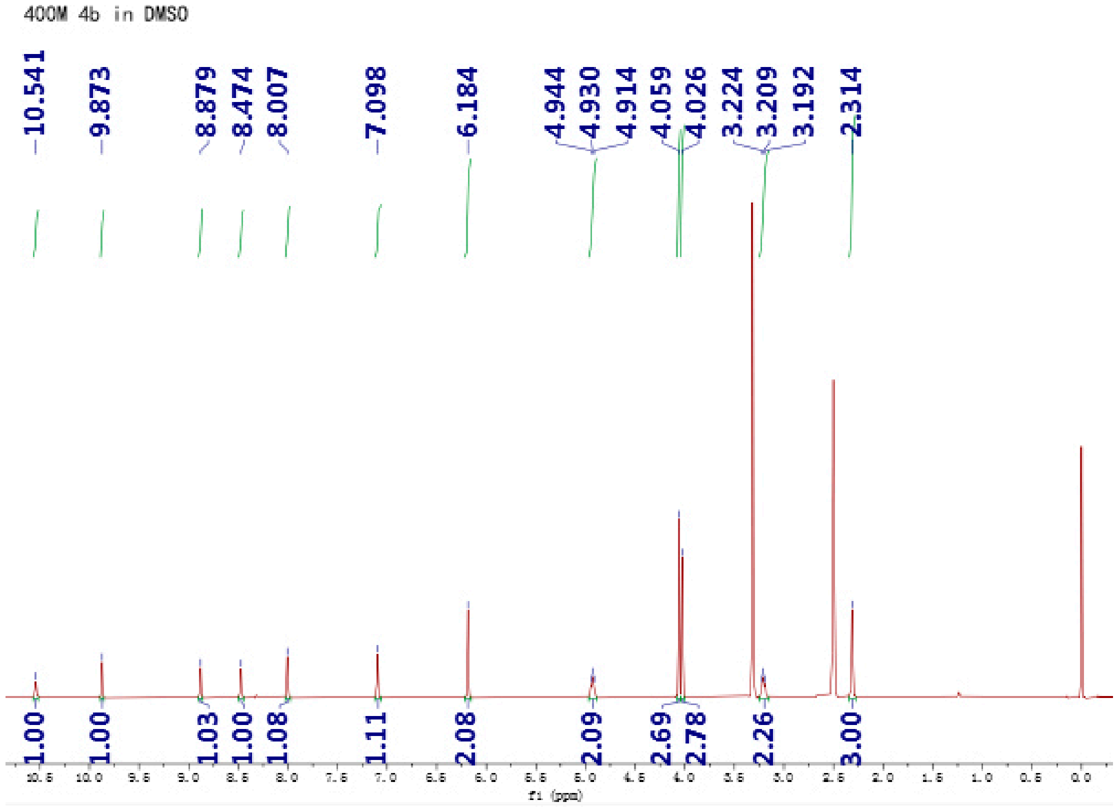

Figure S6. ^{1}H-NMR spectrum of compound **4b**

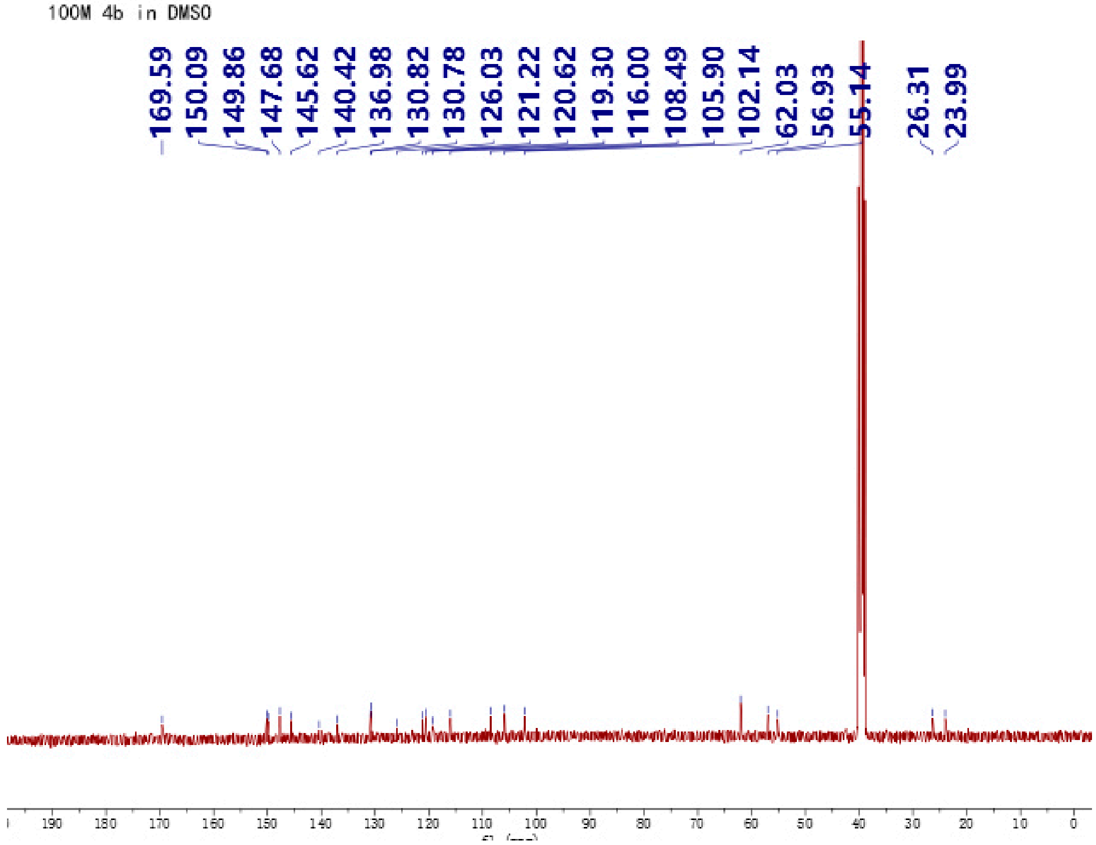

Figure S7. ^{13}C-NMR spectrum of compound **4b**

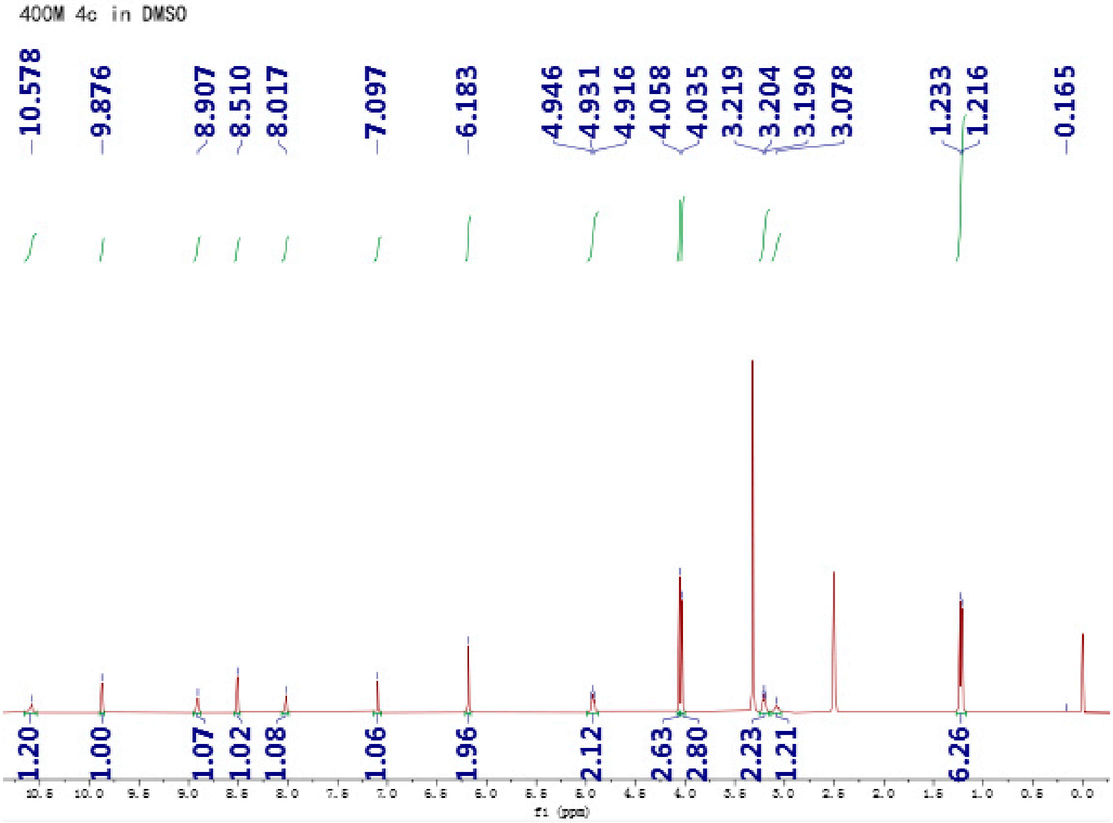

Figure S8. ^{1}H-NMR spectrum of compound **4c**

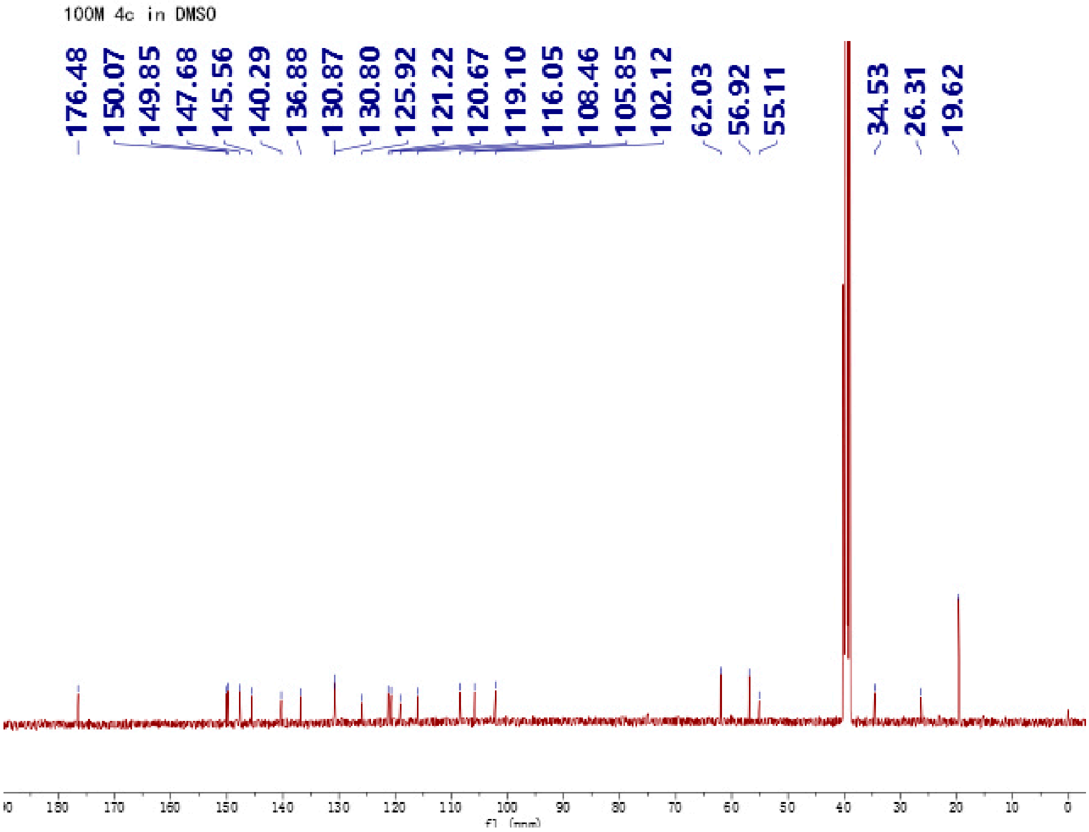

Figure S9. ^{13}C-NMR spectrum of compound **4c**

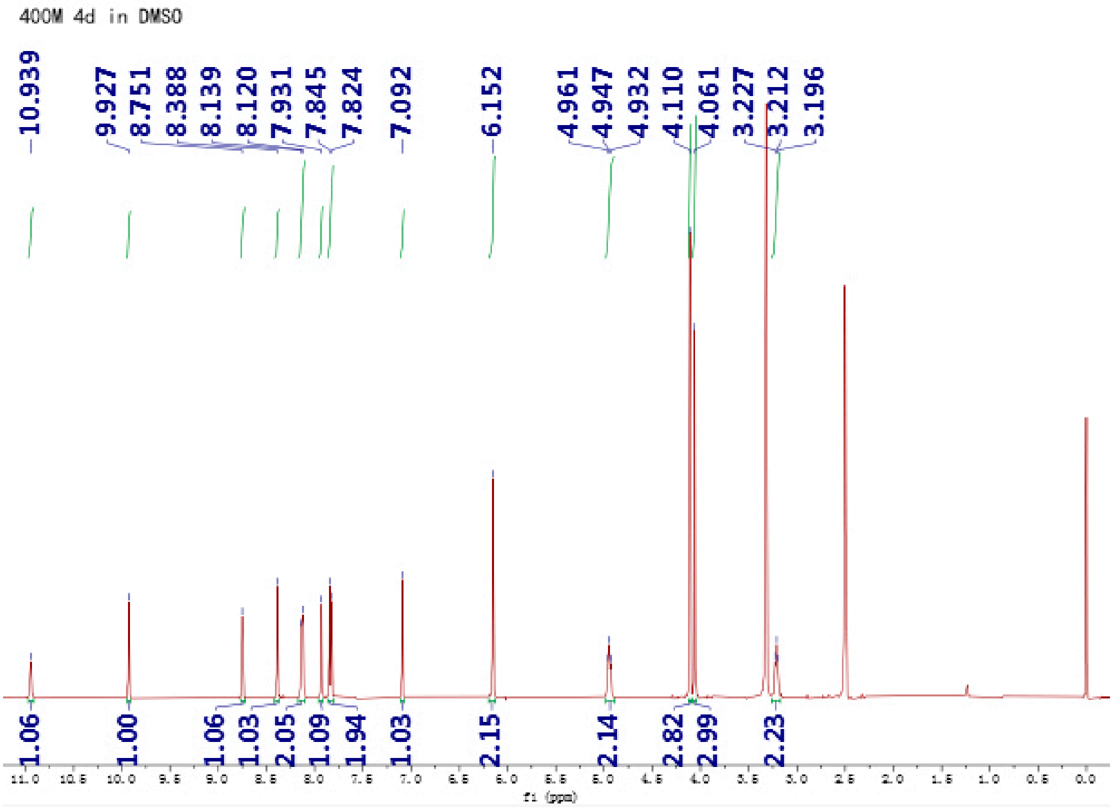

Figure S10. ^{1}H-NMR spectrum of compound **4d**

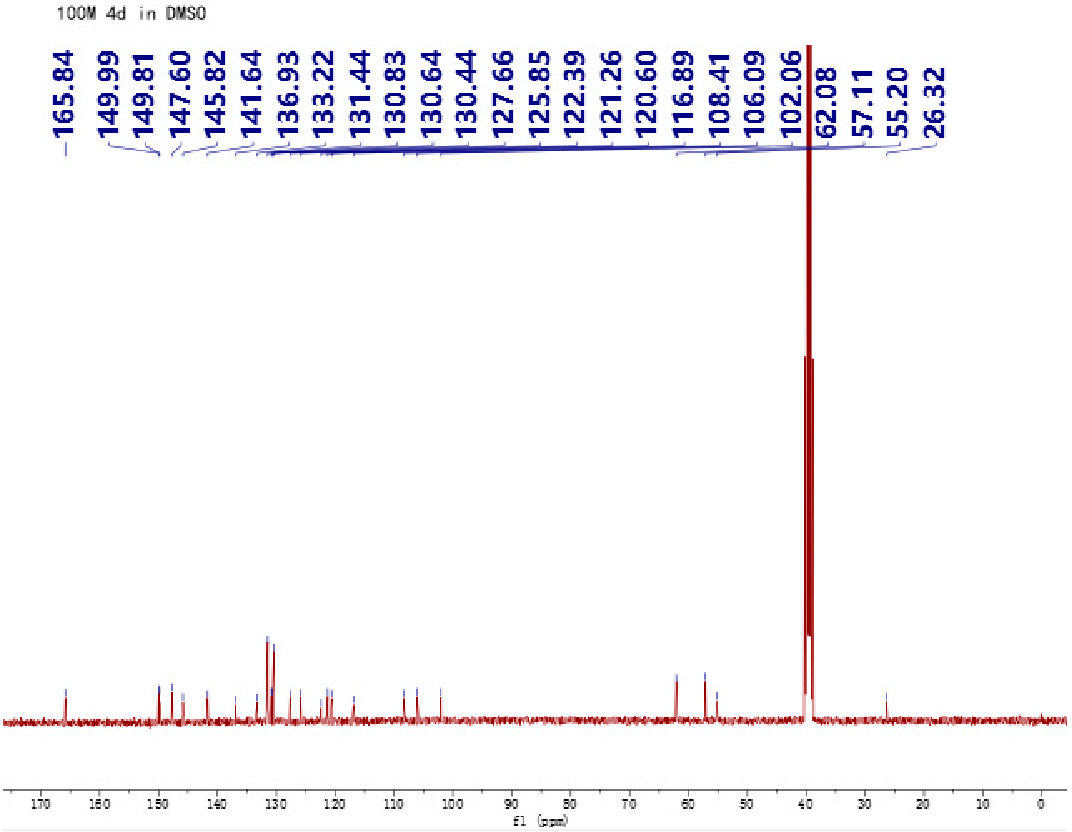

Figure S11. ^{13}C-NMR spectrum of compound **4d**

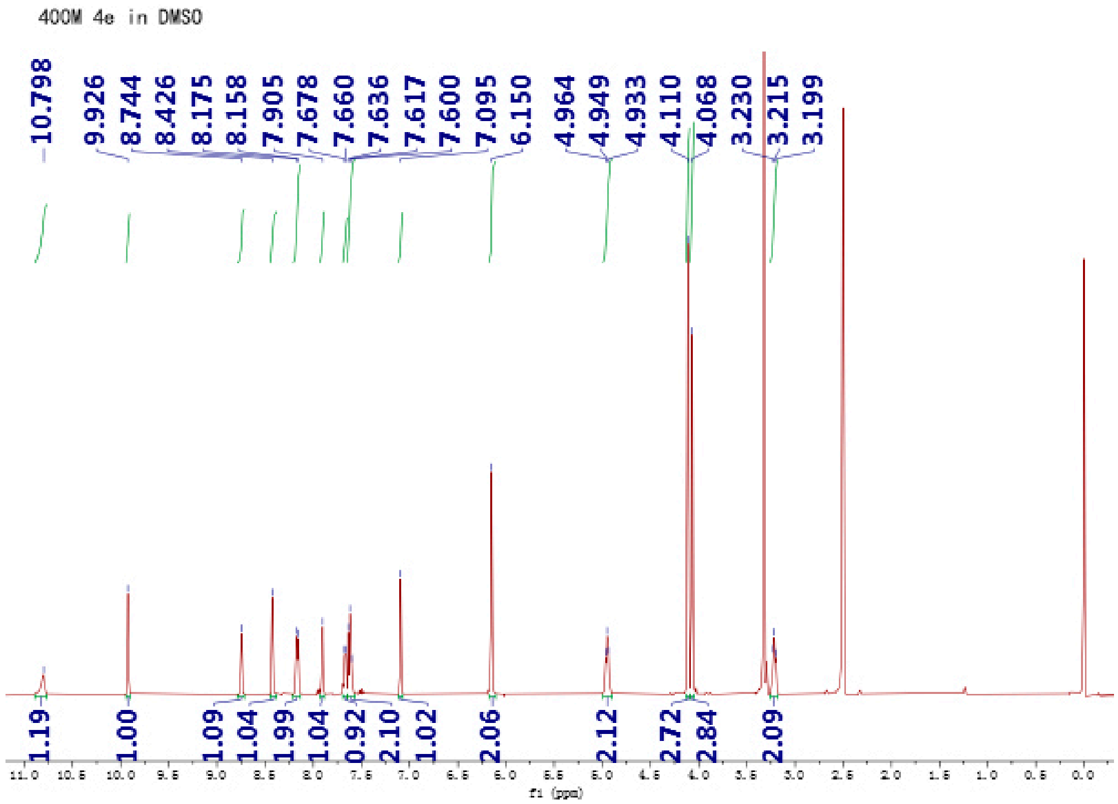

Figure S12. ^{1}H-NMR spectrum of compound **4e**

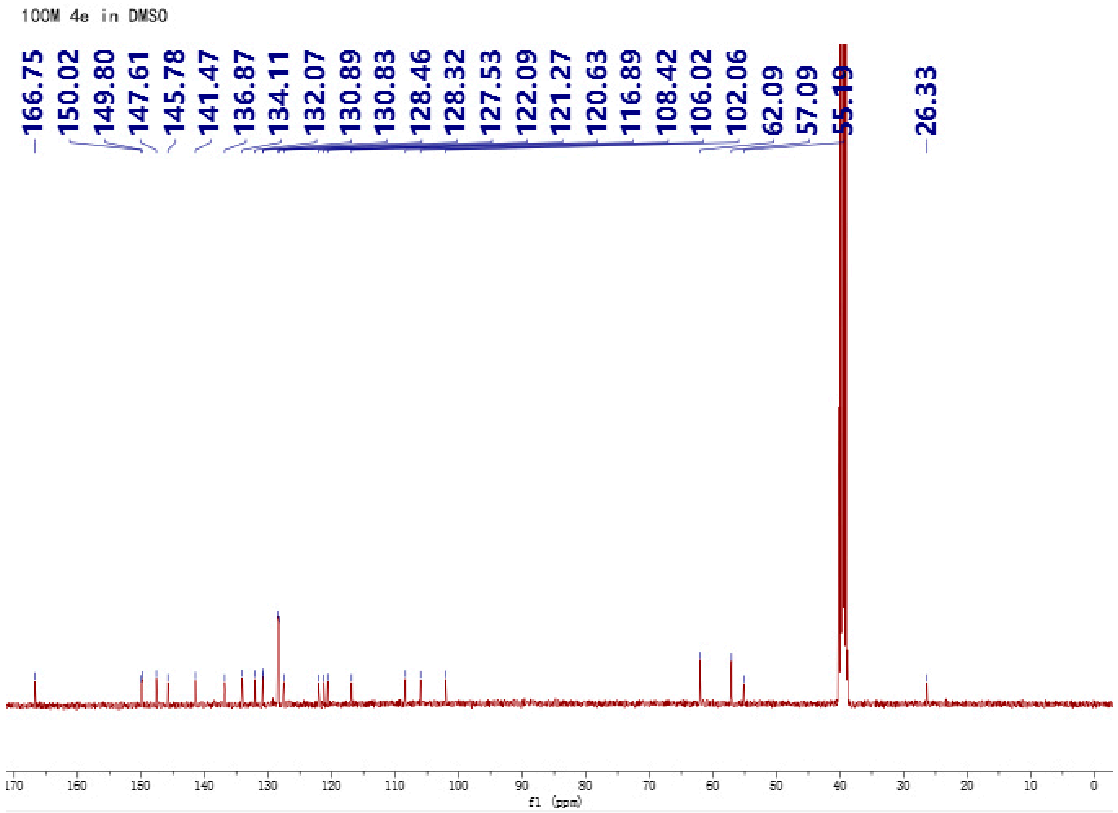

Figure S13. ^{13}C-NMR spectrum of compound **4e**

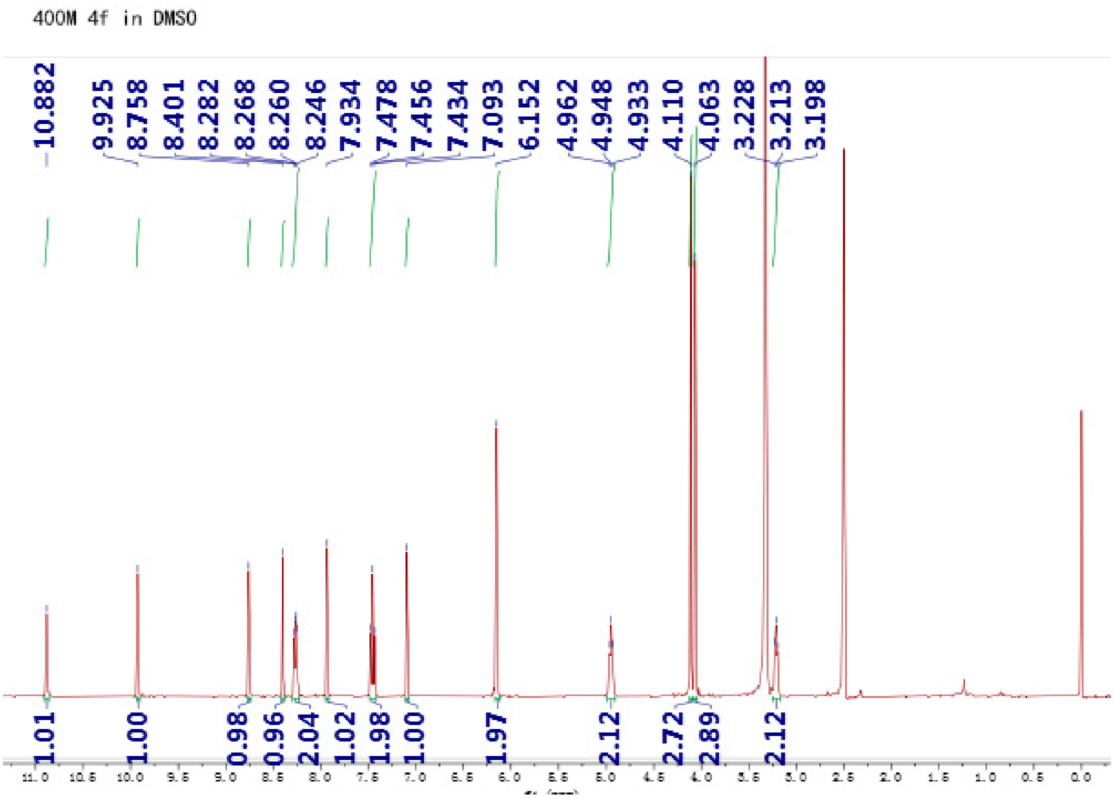

Figure S14. ^{1}H-NMR spectrum of compound **4f**

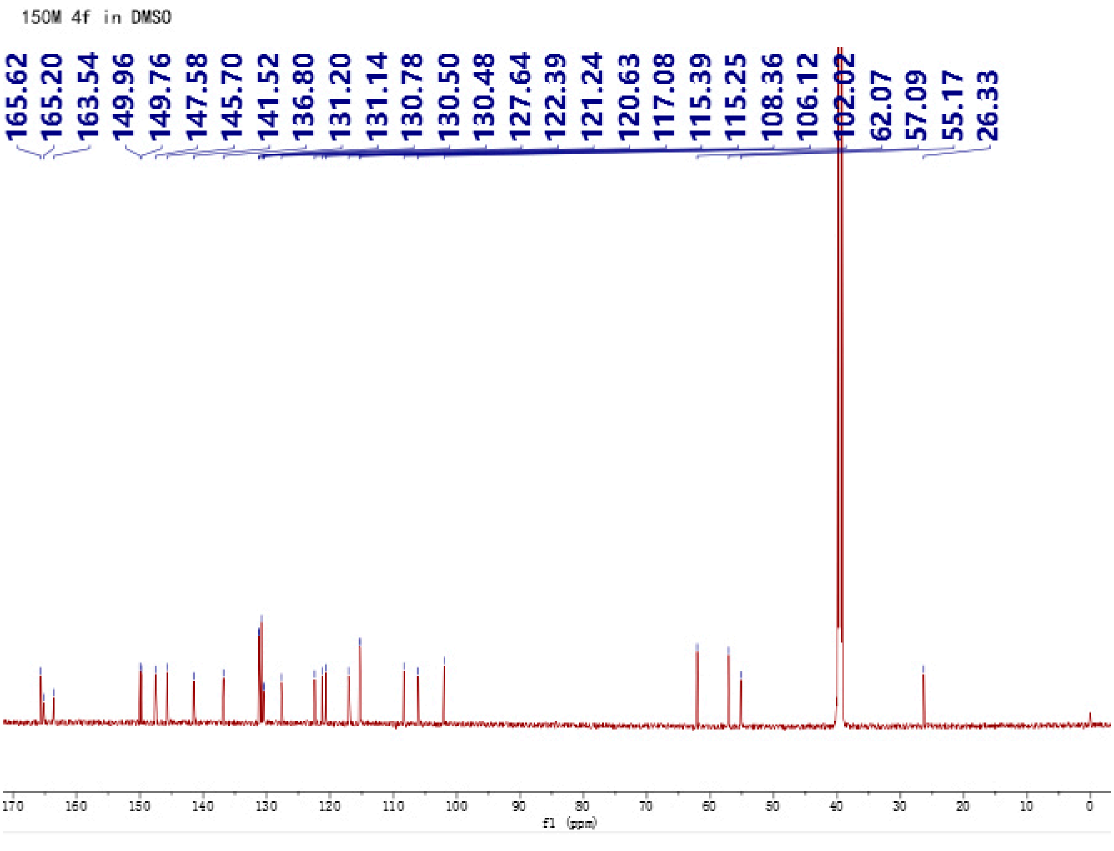

Figure S15. ^{13}C-NMR spectrum of compound **4f**

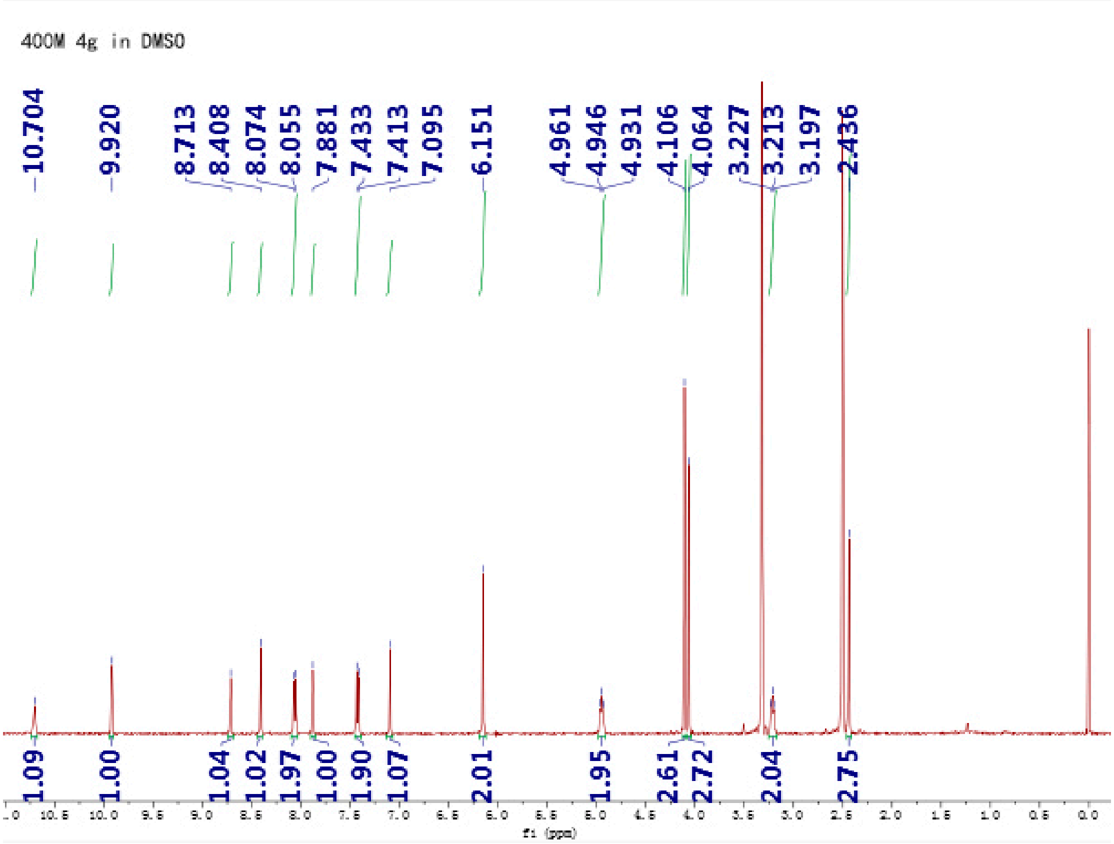

Figure S16. ^{1}H-NMR spectrum of compound **4g**

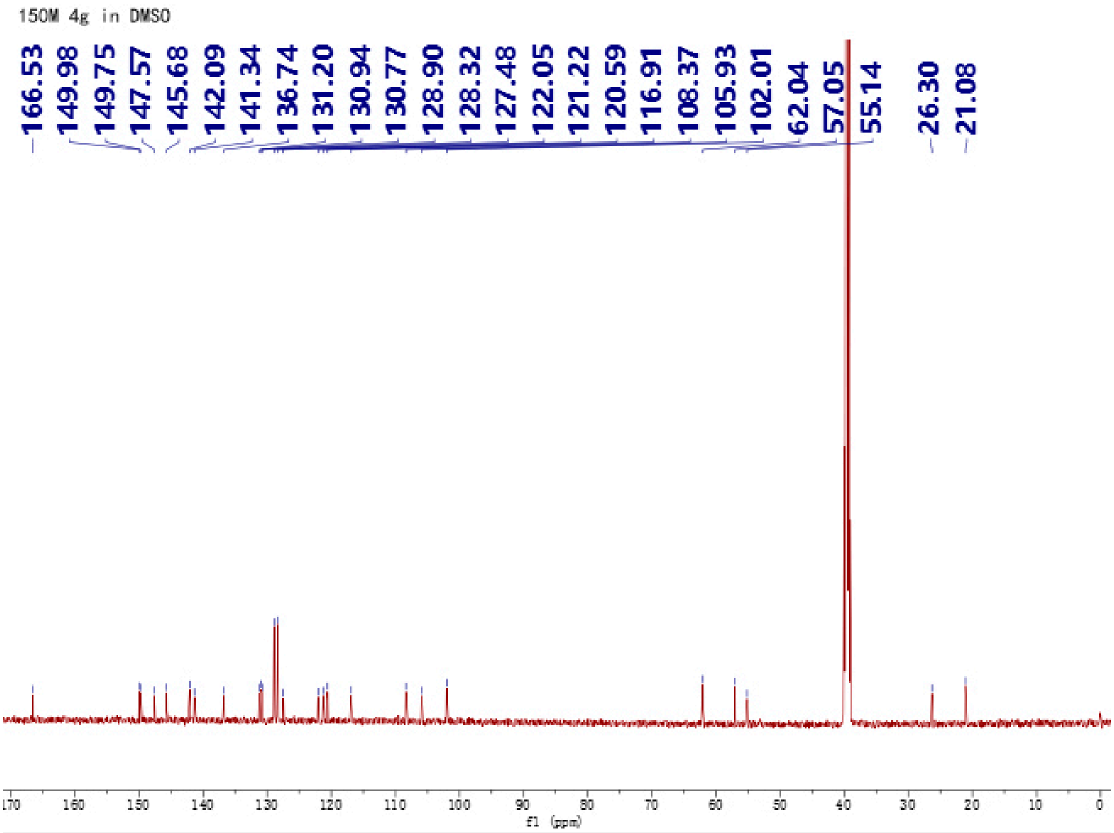

Figure S17. ^{13}C-NMR spectrum of compound **4g**

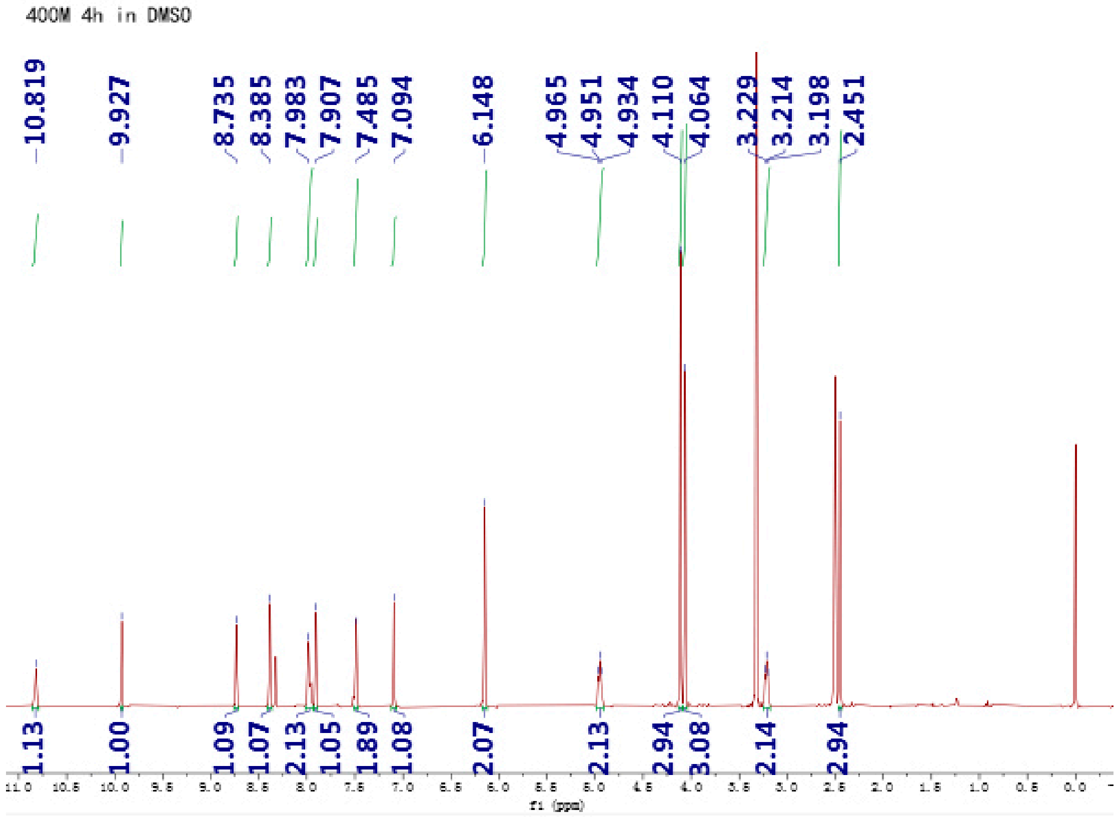

Figure S18. ^{1}H-NMR spectrum of compound **4h**

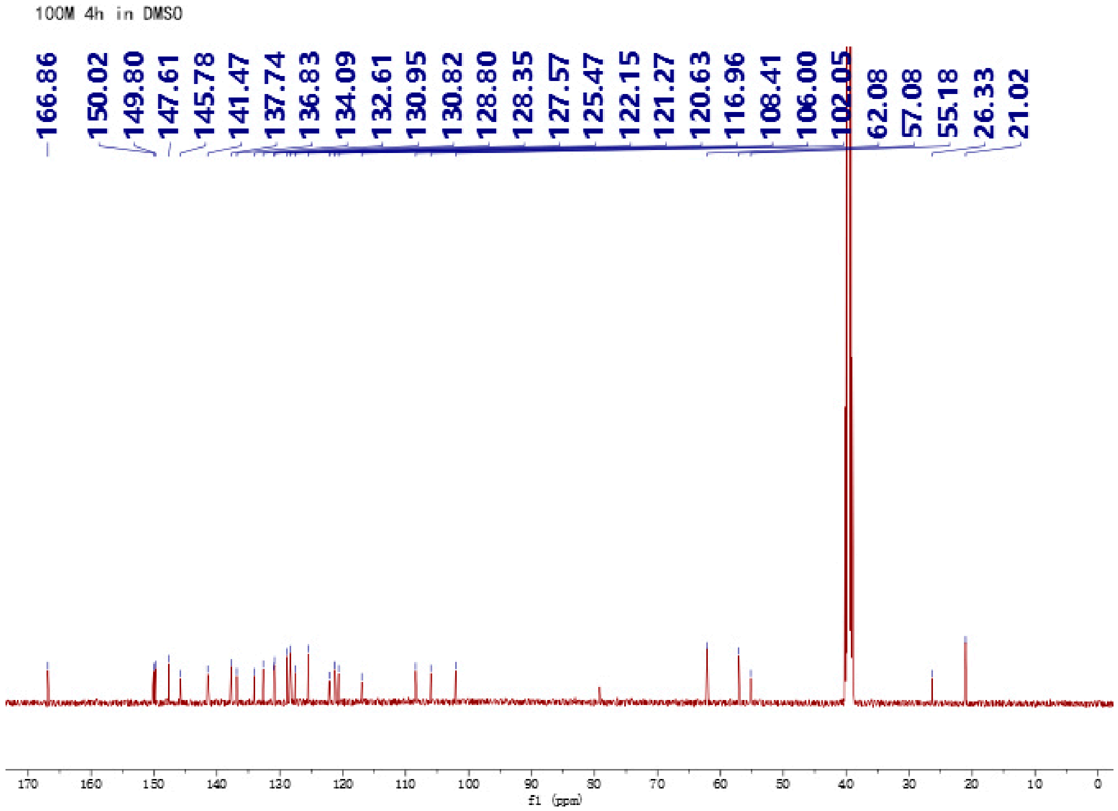

Figure S19. ^{13}C-NMR spectrum of compound **4h**

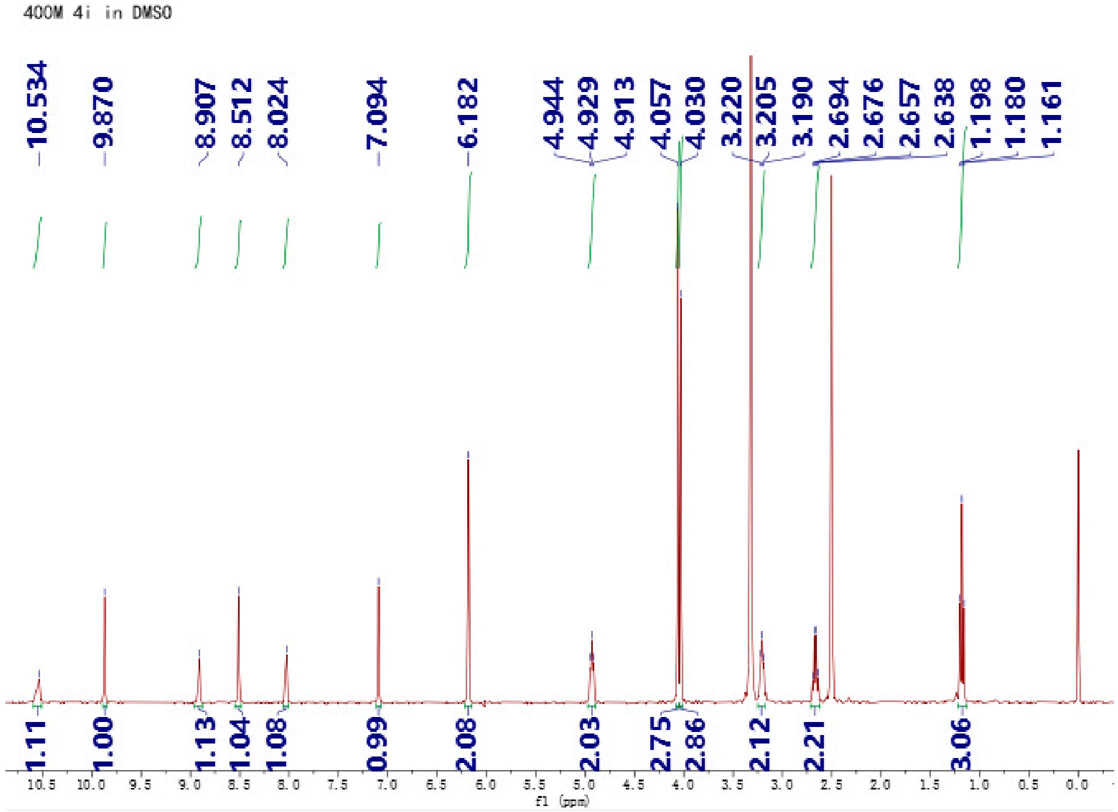

Figure S20. ^{1}H-NMR spectrum of compound **4i**

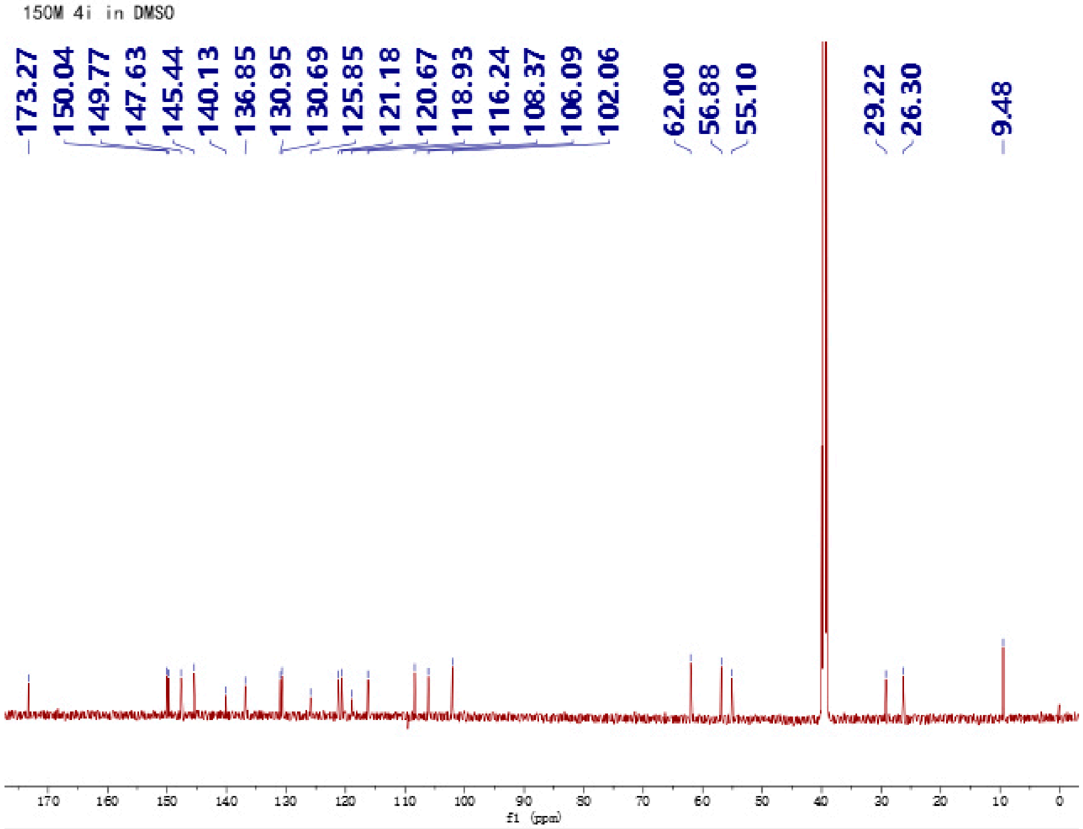

Figure S21. ^{13}C-NMR spectrum of compound **4i**

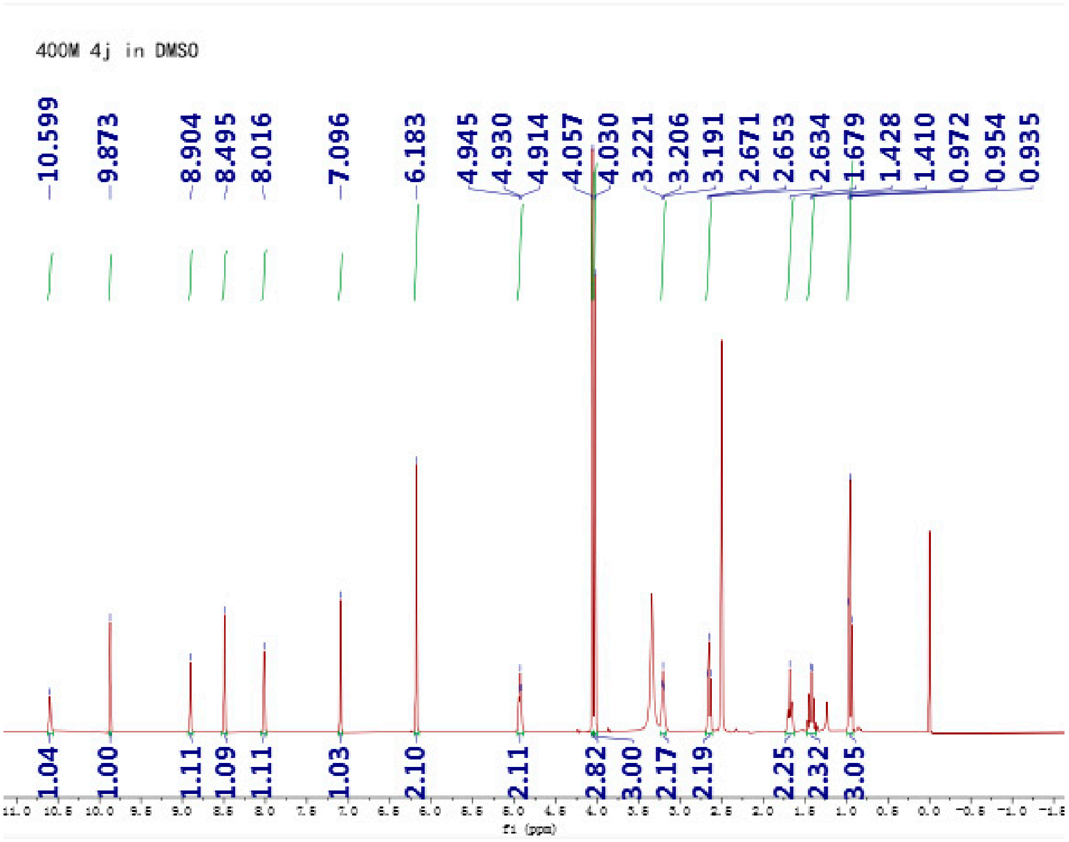

Figure S22. ^{1}H-NMR spectrum of compound **4j**

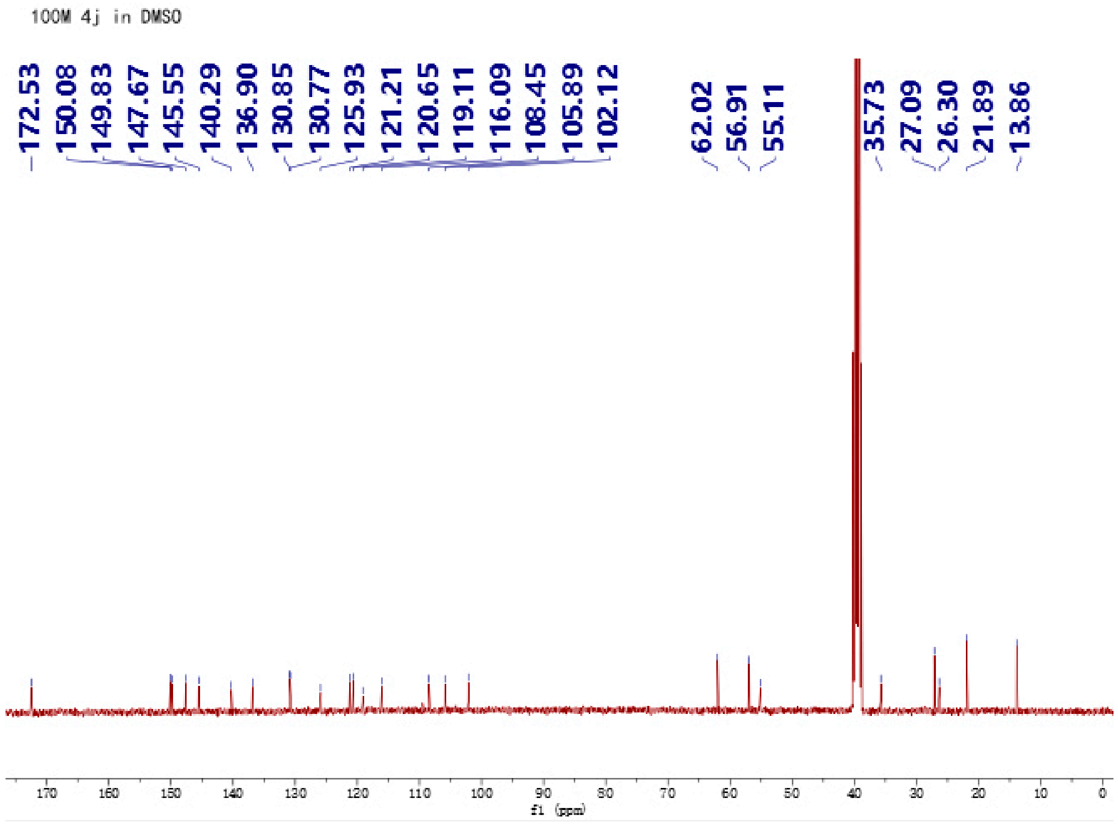

Figure S23. ^{13}C-NMR spectrum of compound **4j**

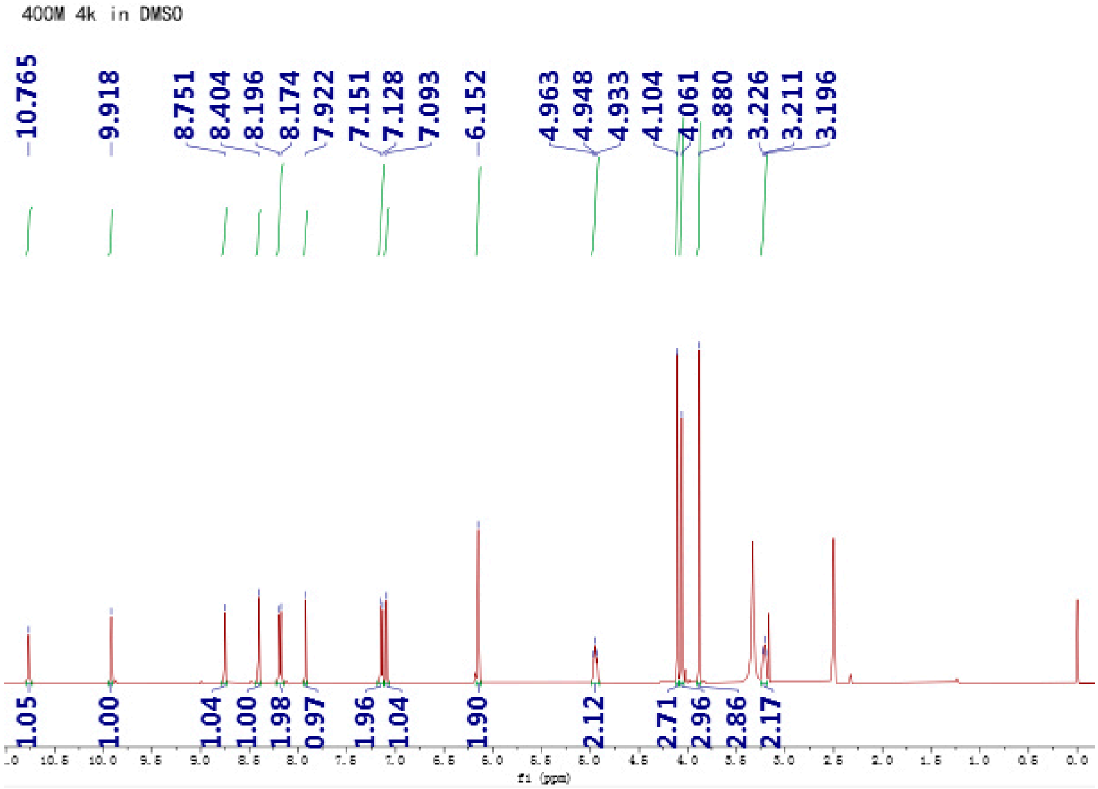

Figure S24. ^{1}H-NMR spectrum of compound **4k**

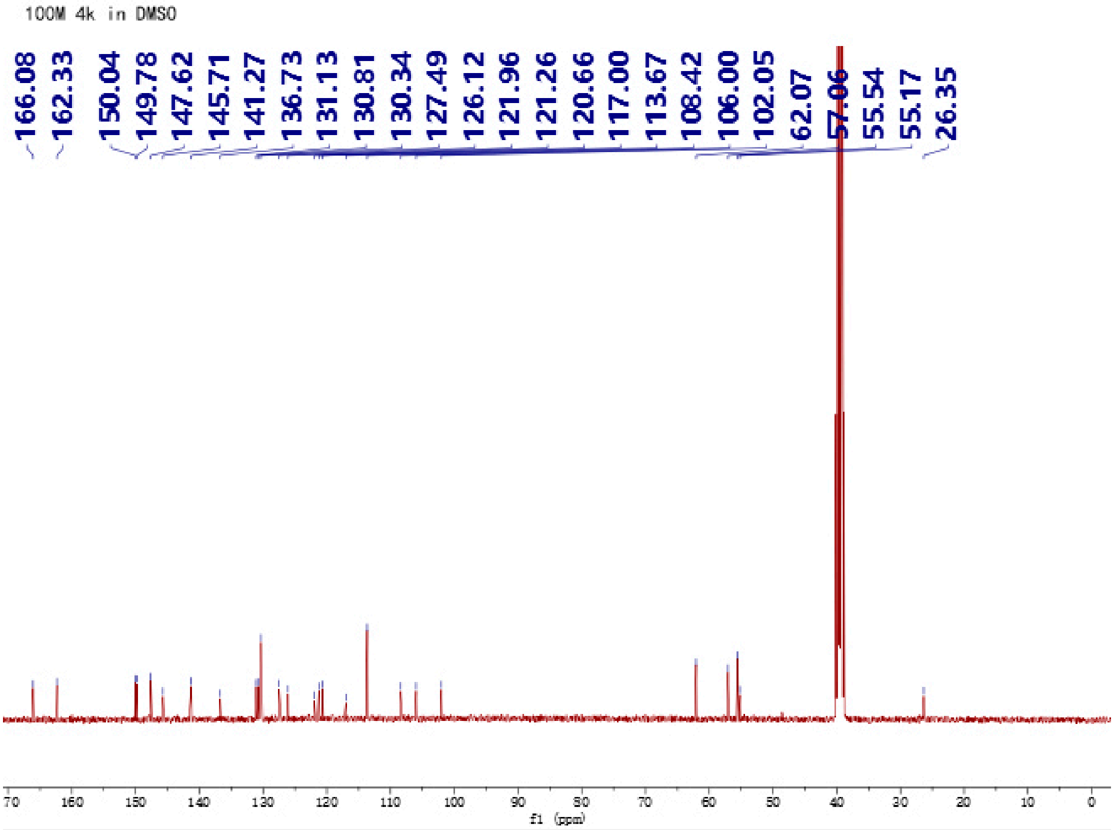

Figure S25. ^{13}C-NMR spectrum of compound **4k**

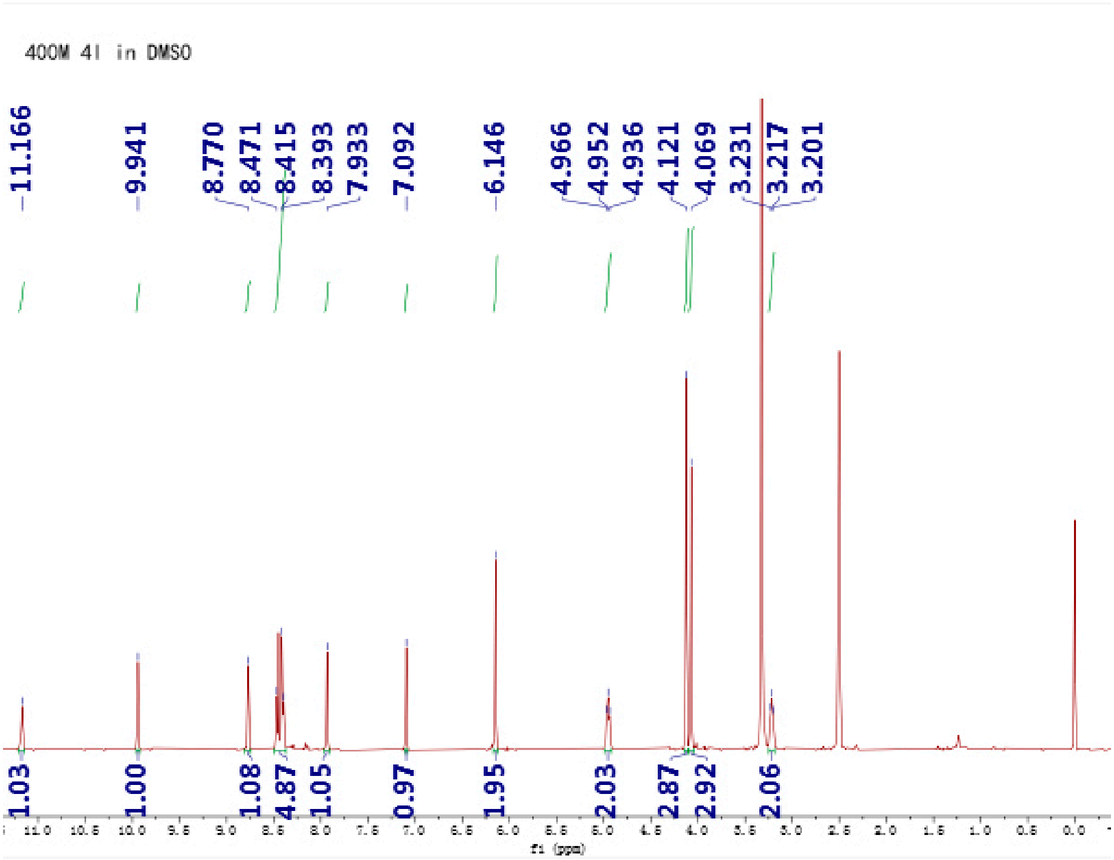

Figure S26. ^{1}H-NMR spectrum of compound **4l**

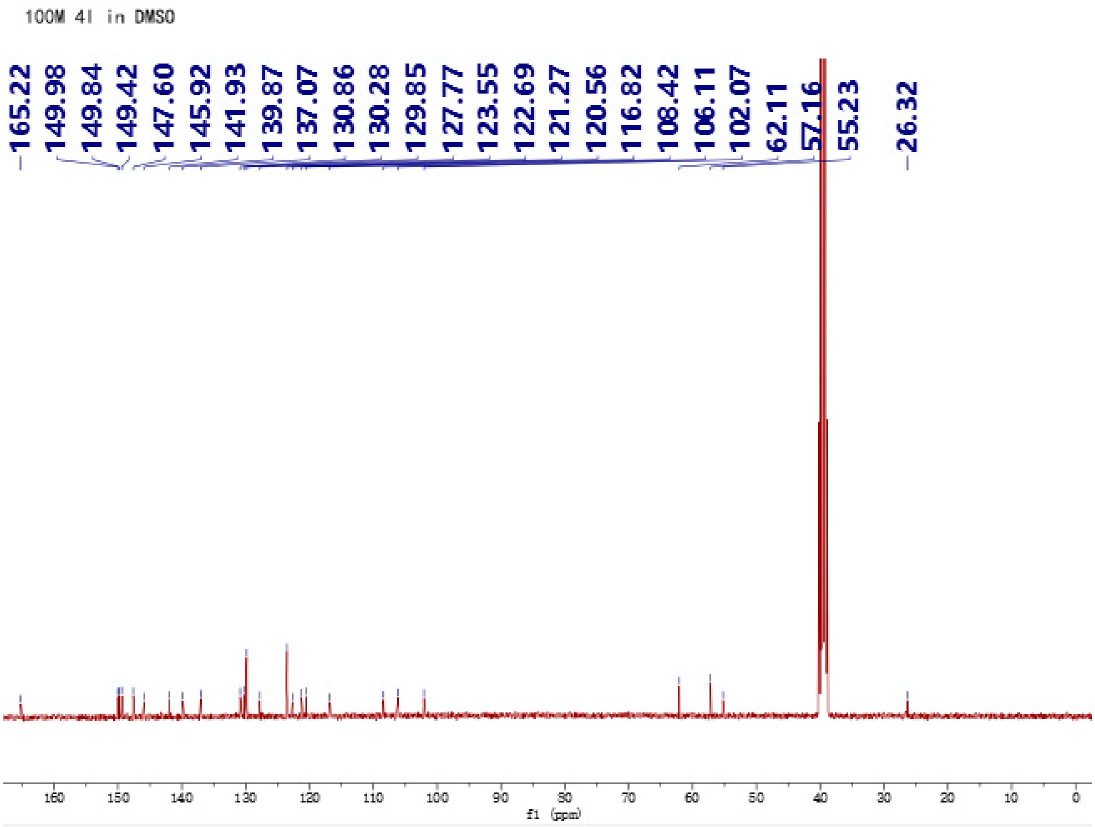

Figure S27. ^{13}C-NMR spectrum of compound **4l**

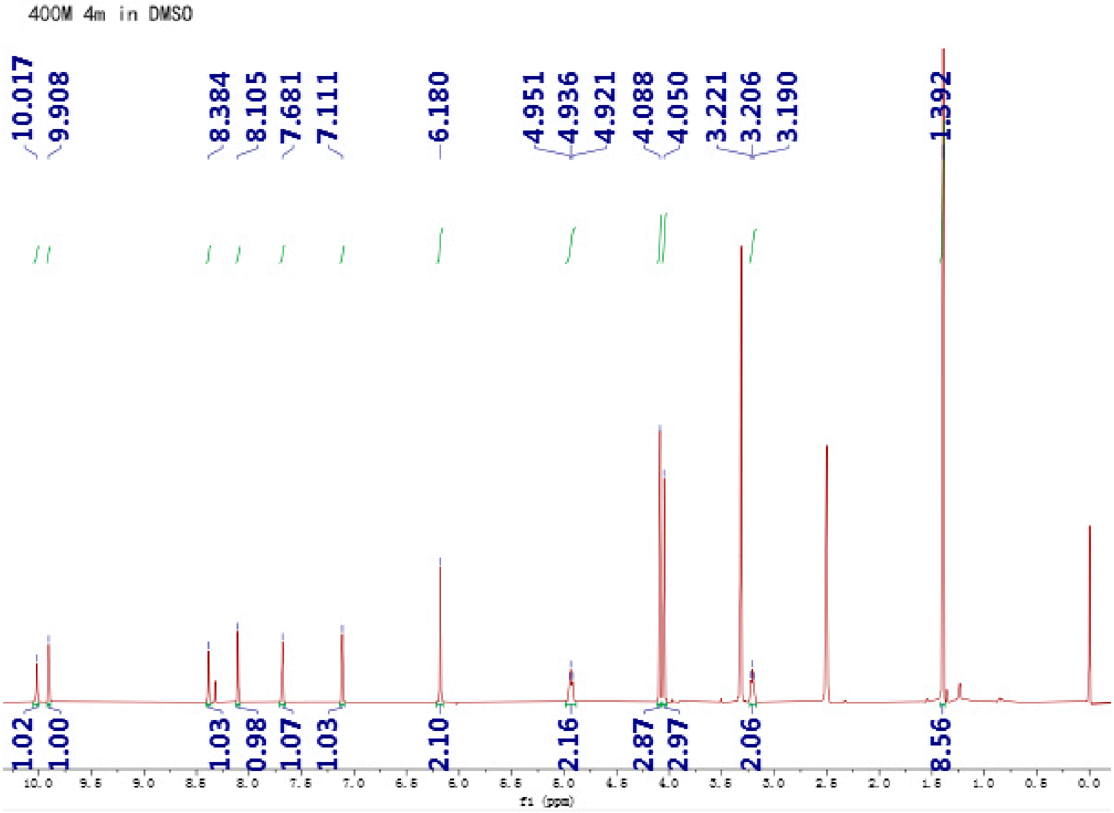

Figure S28. ^{1}H-NMR spectrum of compound **4m**

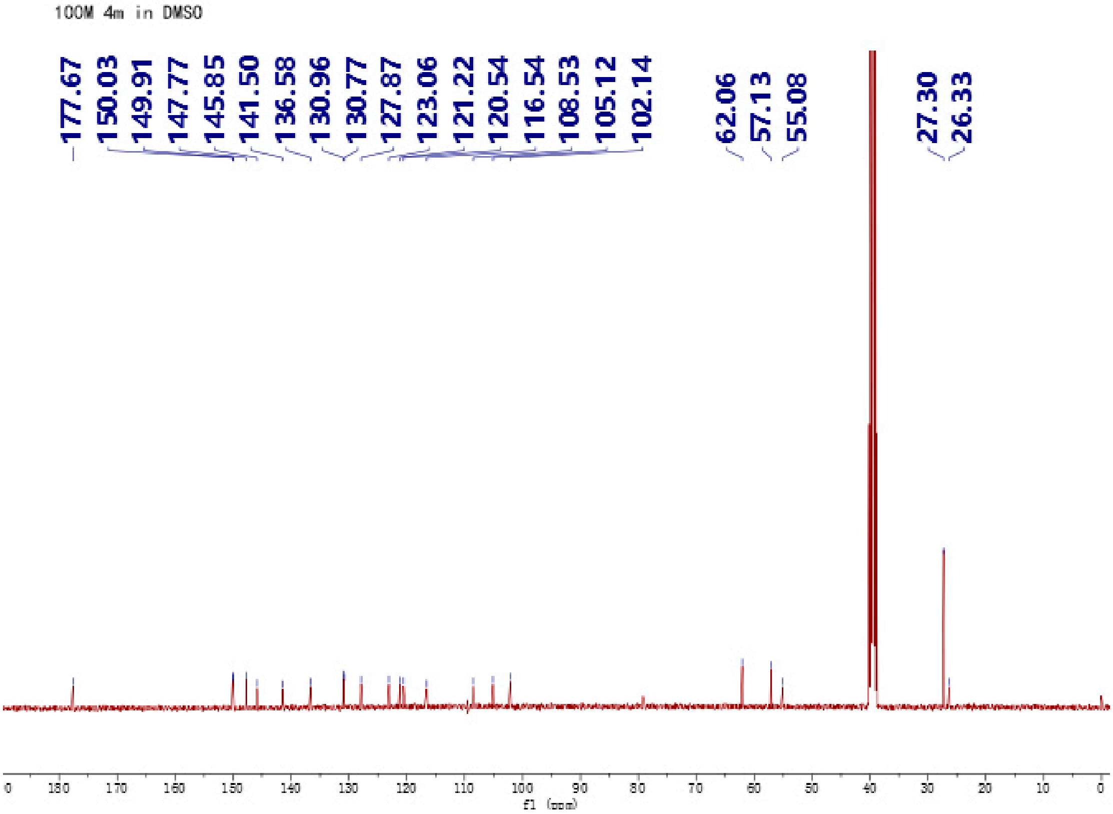

Figure S29. ^{13}C-NMR spectrum of compound **4m**

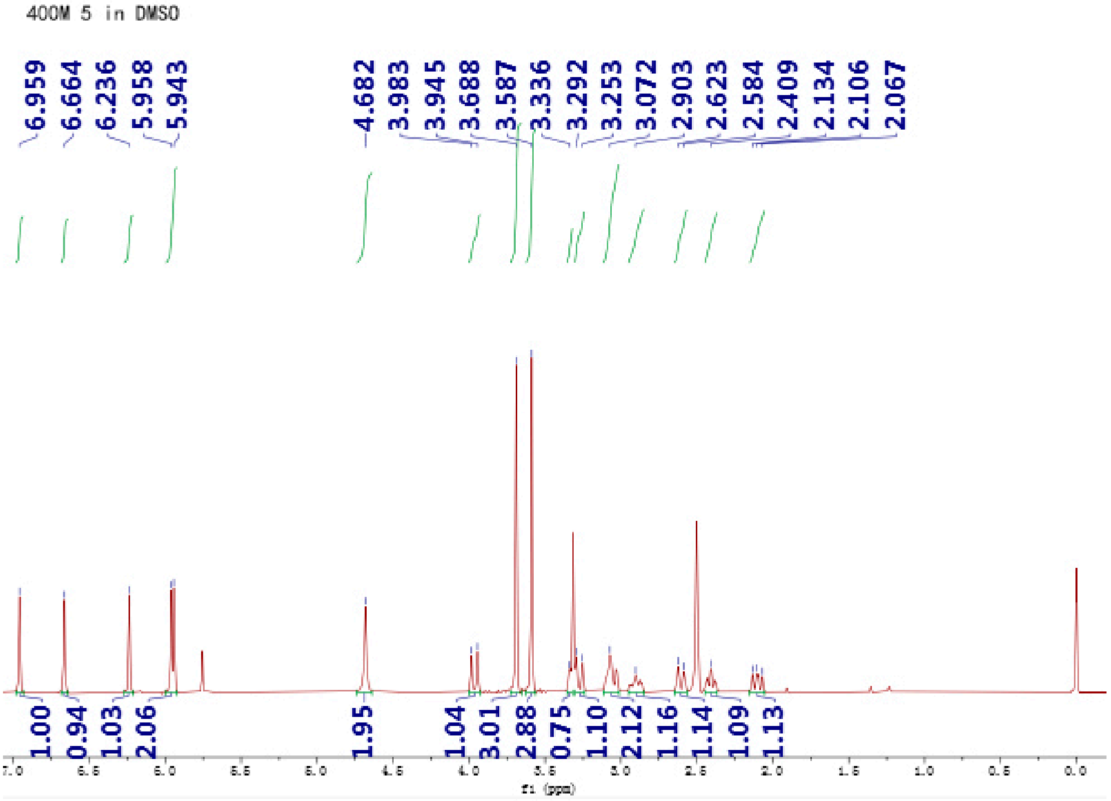

Figure S30. ^{1}H-NMR spectrum of compound **5**

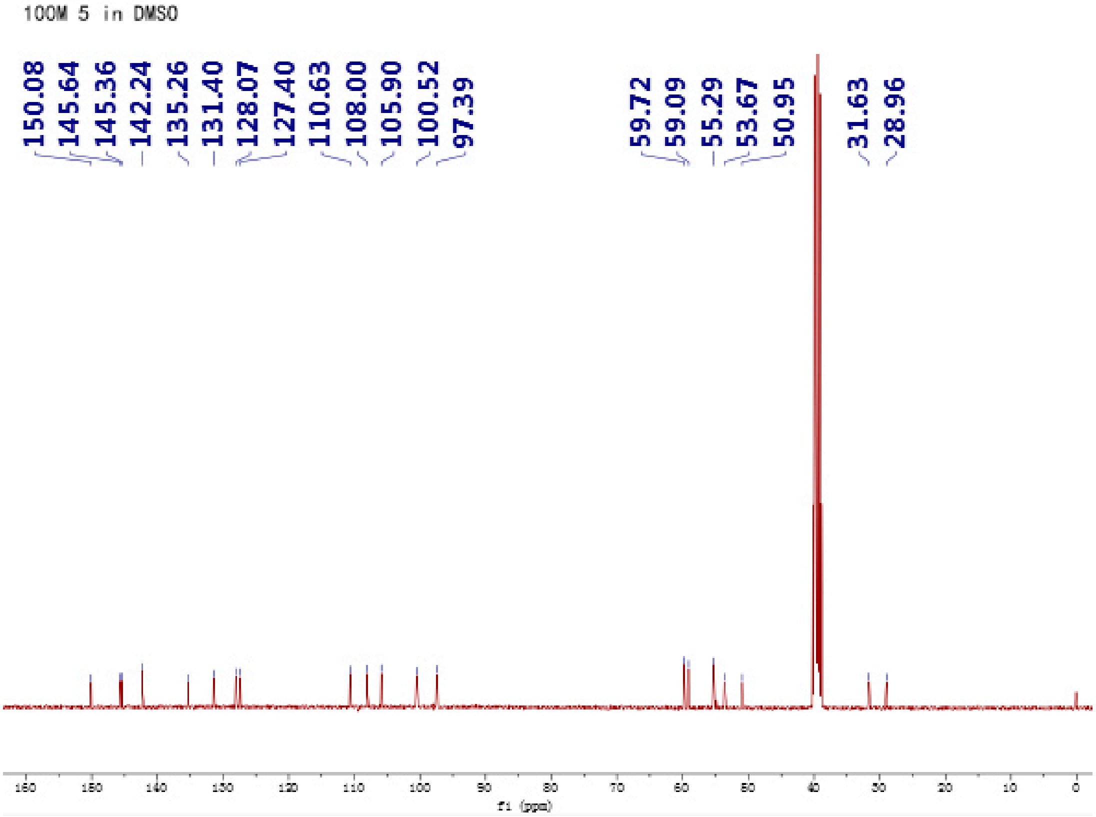

Figure S31. ^{13}C-NMR spectrum of compound **5**

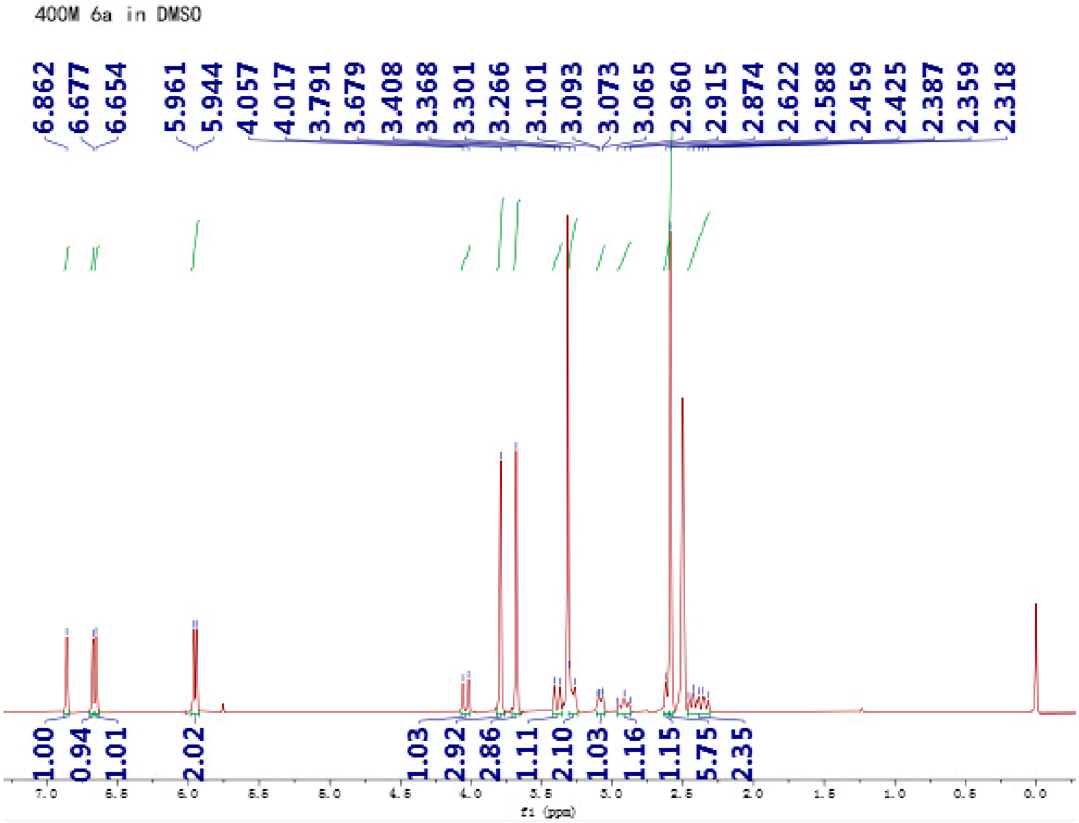

Figure S32. ^{1}H-NMR spectrum of compound **6a**

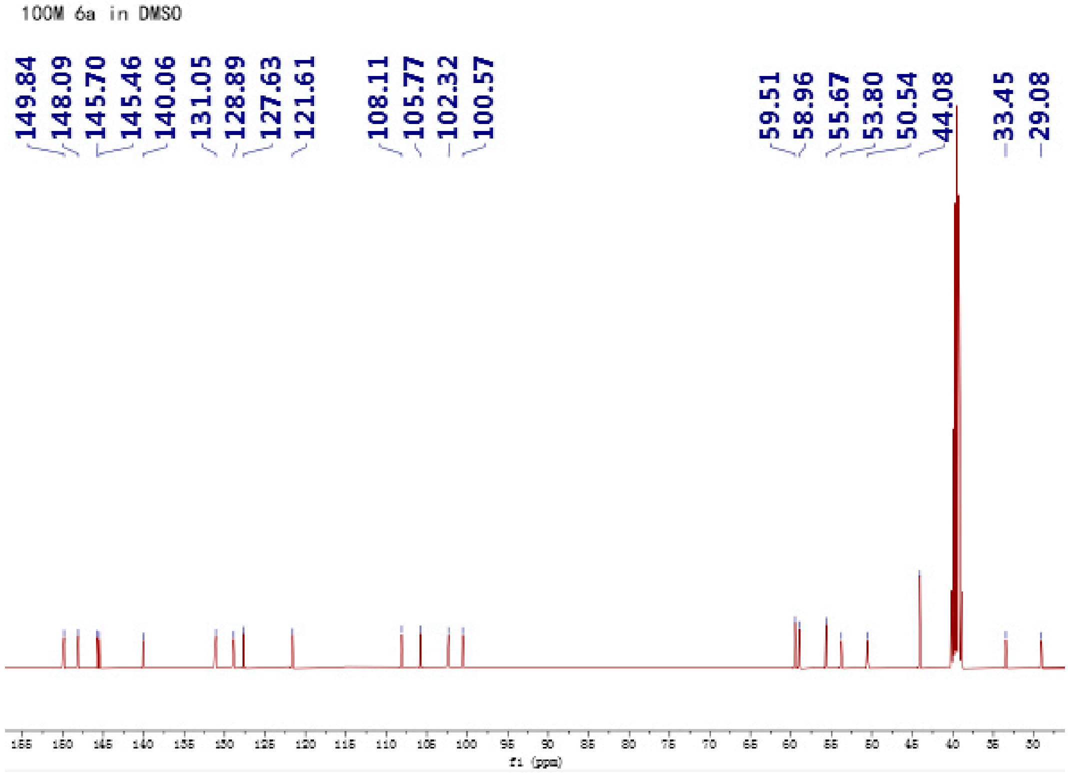

Figure S33. ^{13}C-NMR spectrum of compound **6a**

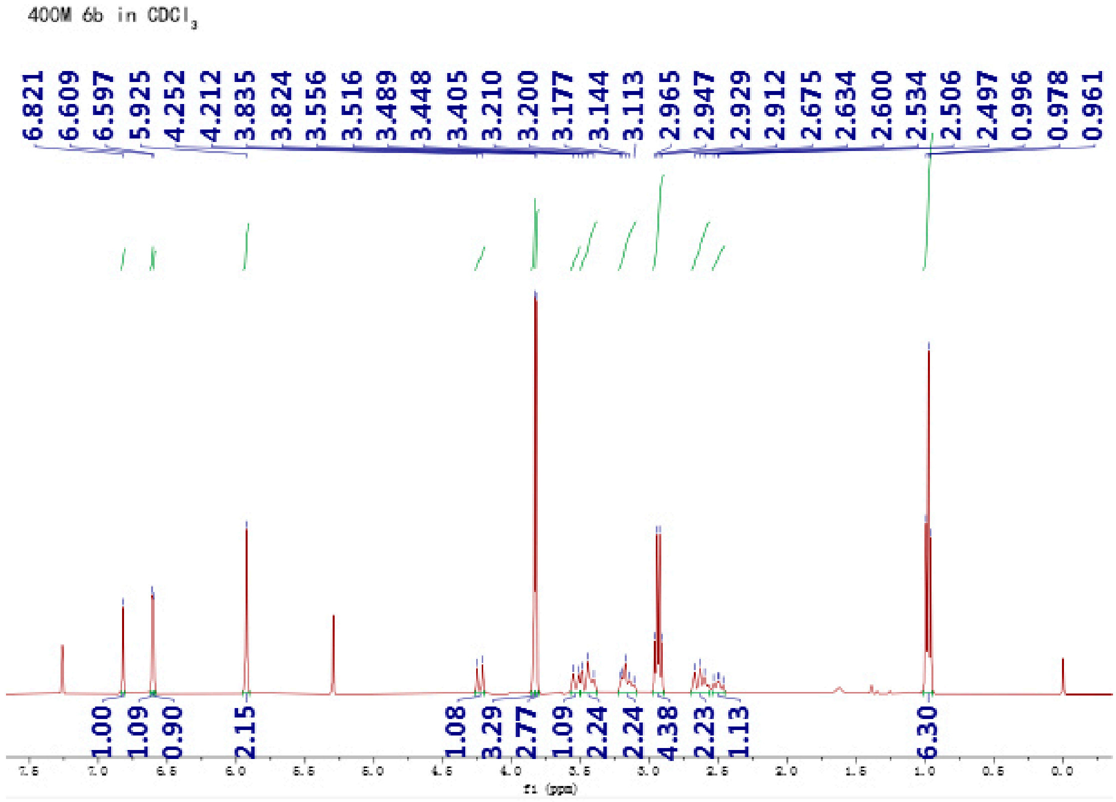

Figure S34. ^{1}H-NMR spectrum of compound **6b**

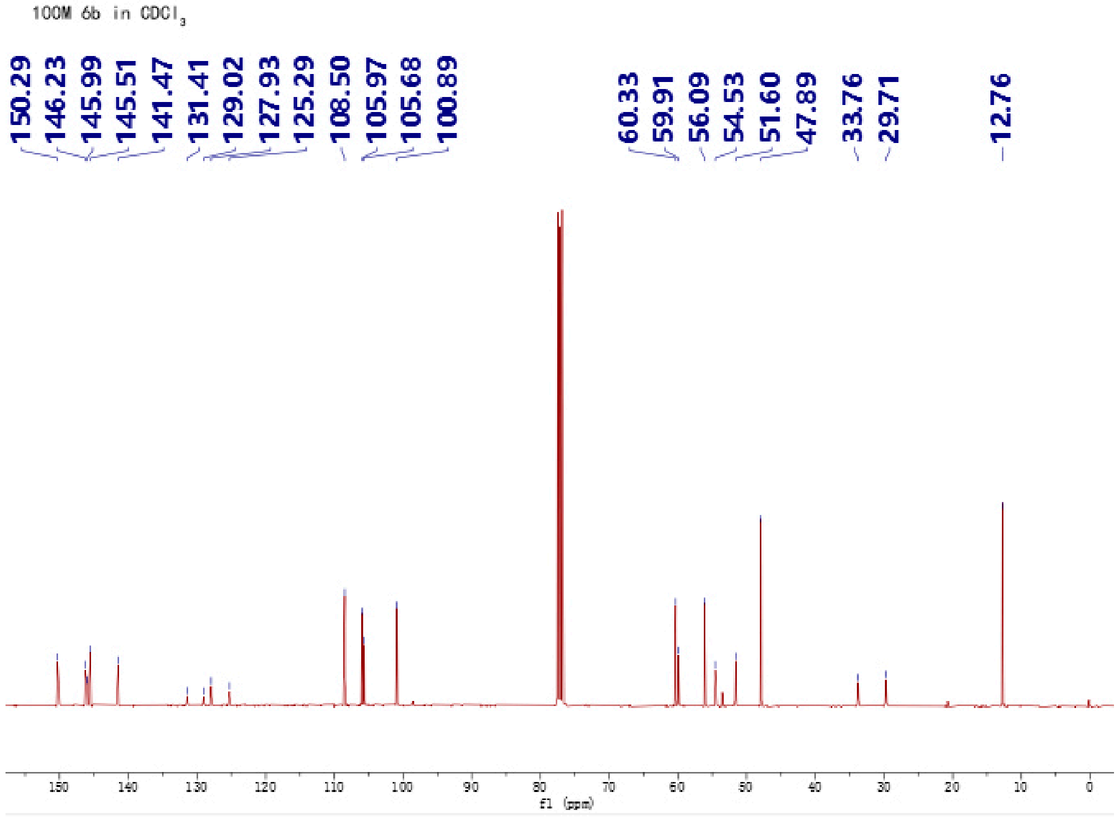

Figure S35. ^{13}C-NMR spectrum of compound **6b**

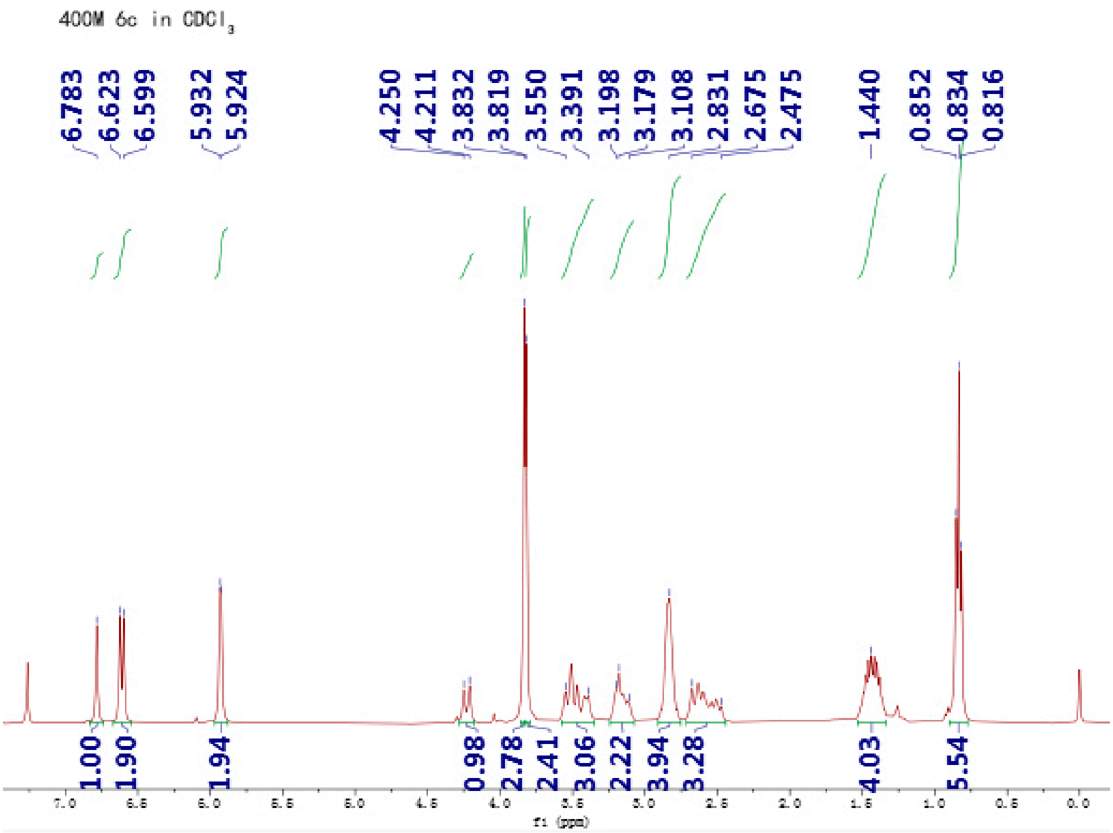

Figure S36. ^{1}H-NMR spectrum of compound **6c**

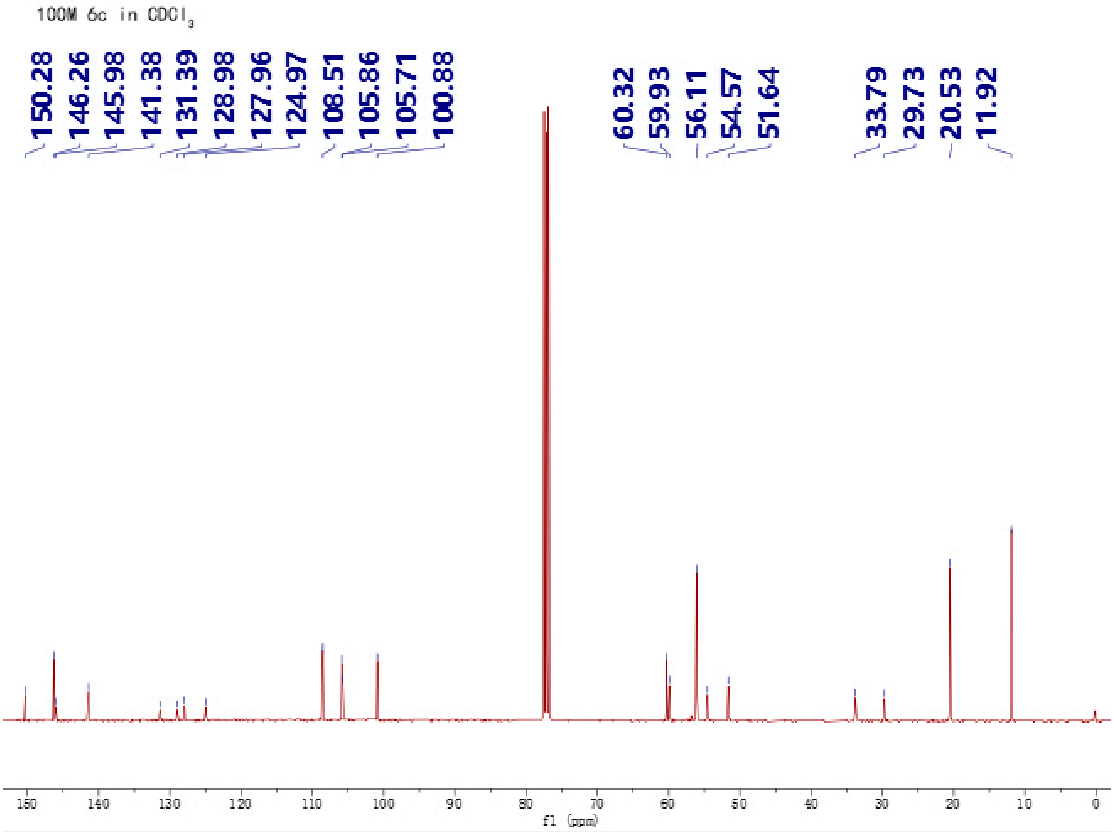

Figure S37. ^{13}C-NMR spectrum of compound **6c**

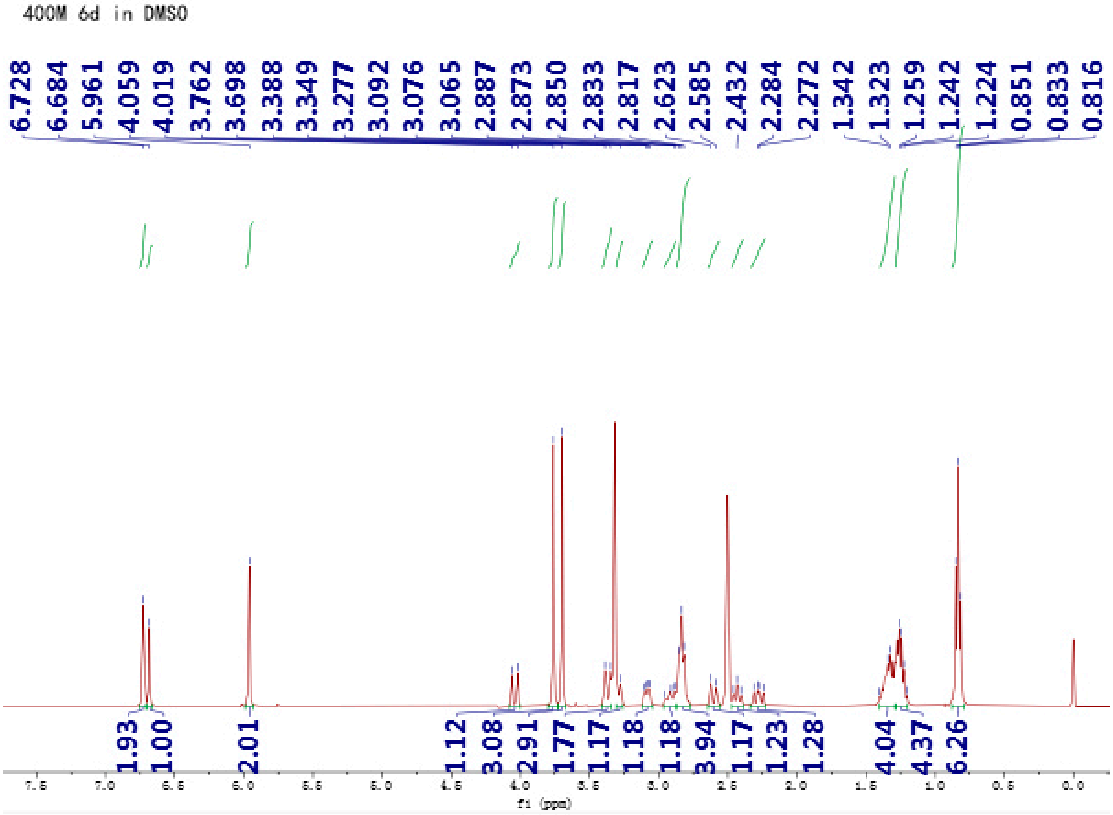

Figure S38. ^{1}H-NMR spectrum of compound **6d**

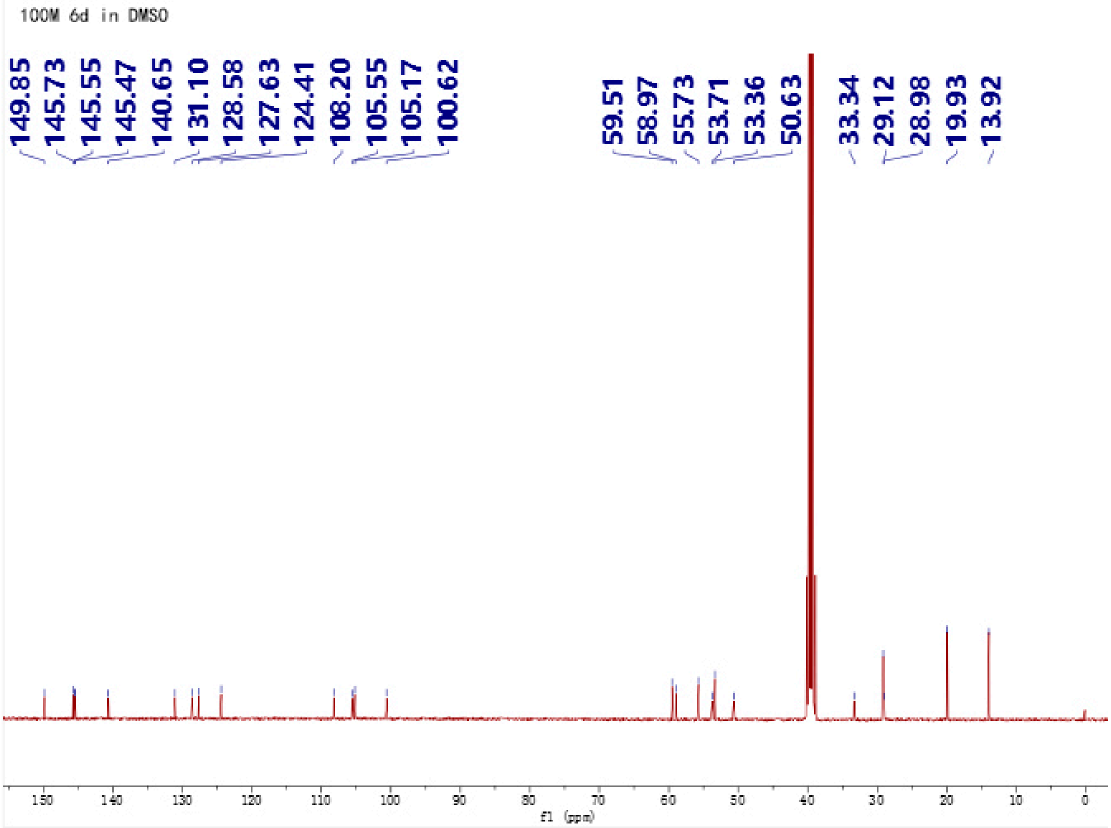

Figure S39. ^{13}C-NMR spectrum of compound **6d**

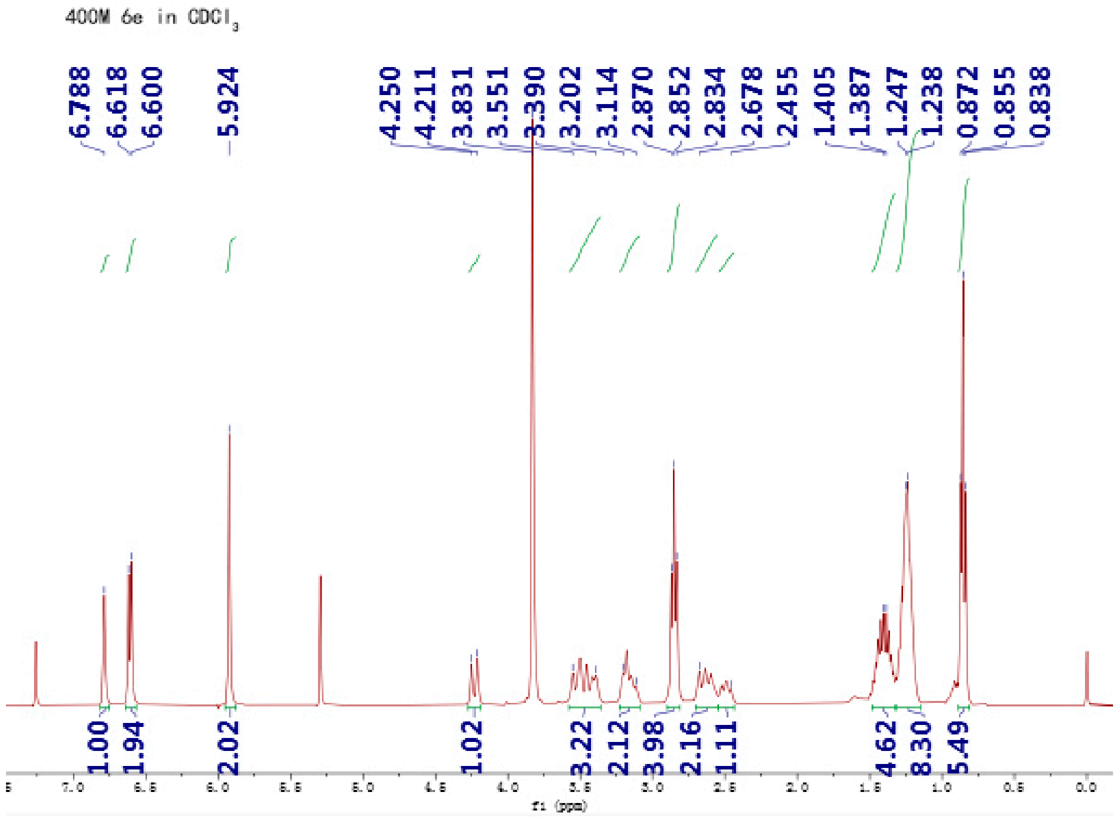

Figure S40. ^{1}H-NMR spectrum of compound **6e**

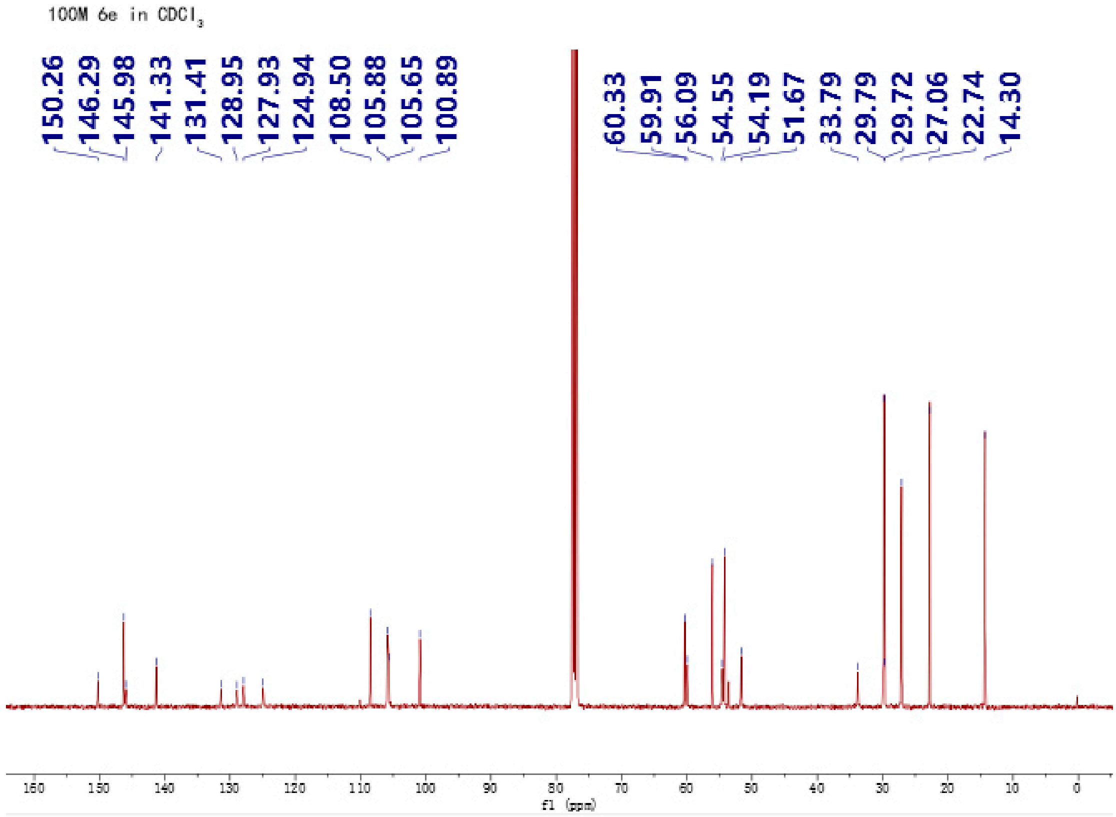

Figure S41. ^{13}C-NMR spectrum of compound **6e**

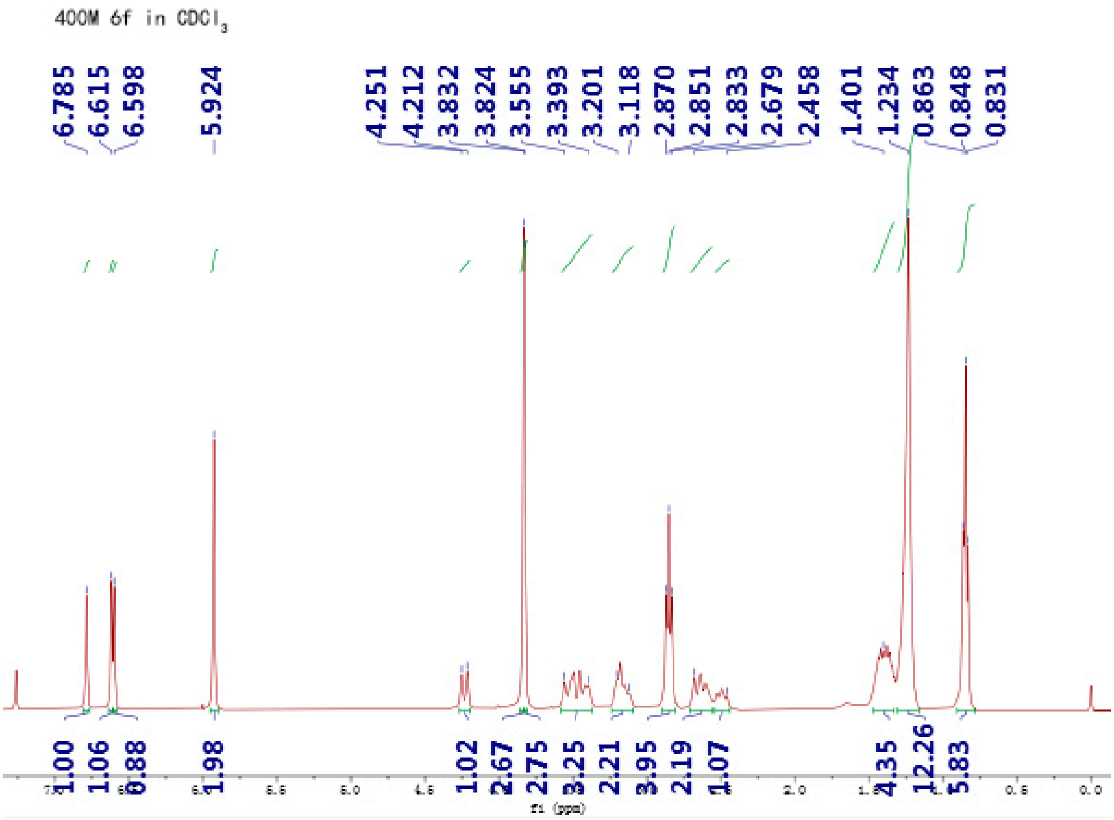

Figure S42. ^{1}H-NMR spectrum of compound **6f**

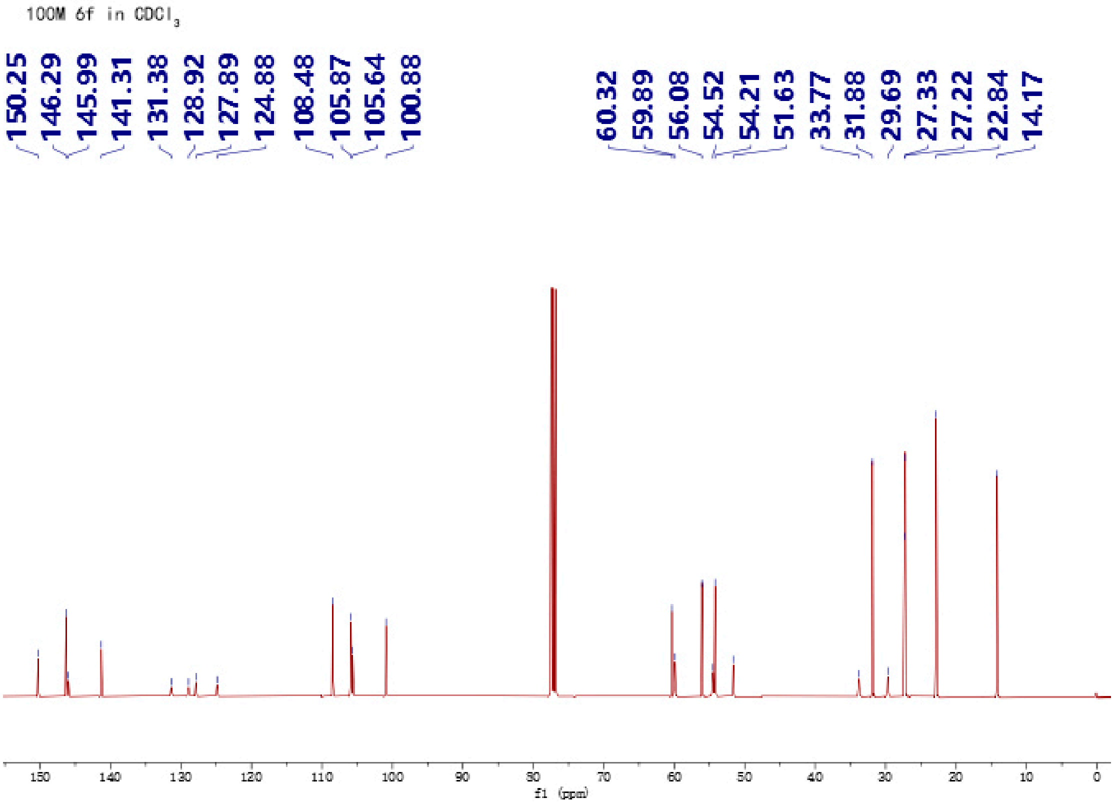

Figure S43. ^{13}C-NMR spectrum of compound **6f**

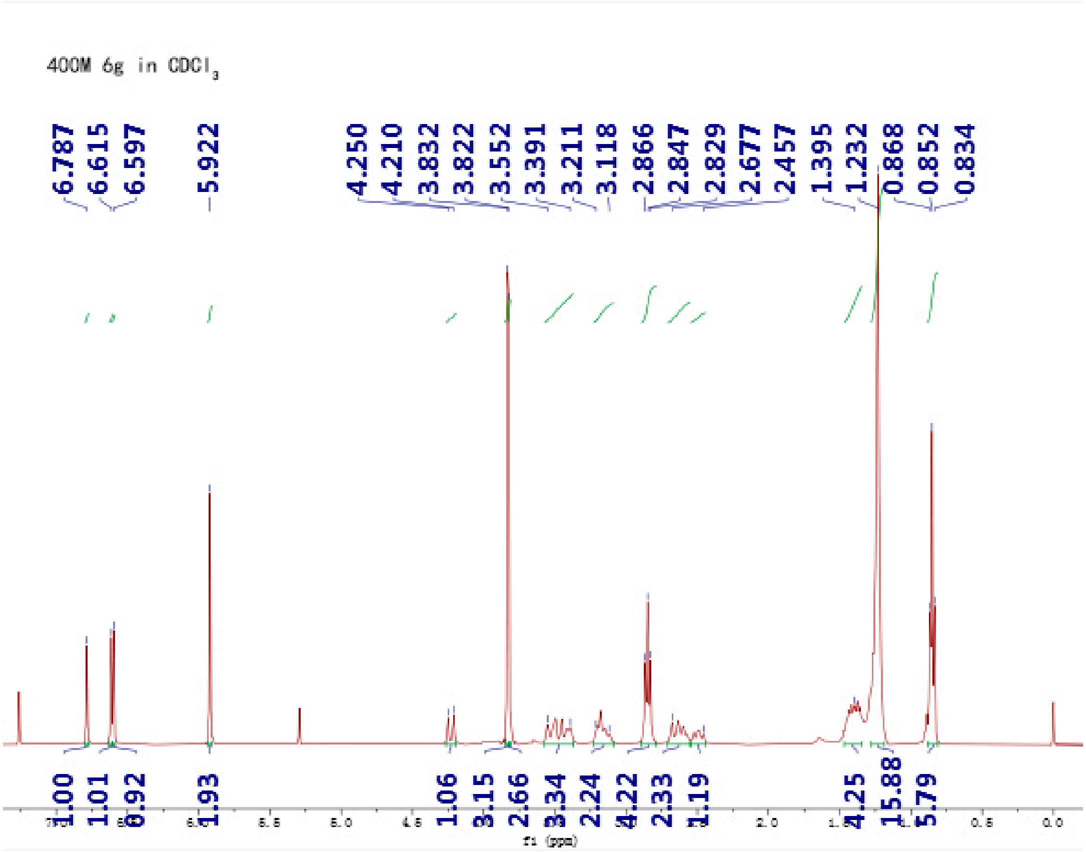

Figure S44. ^{1}H-NMR spectrum of compound **6g**

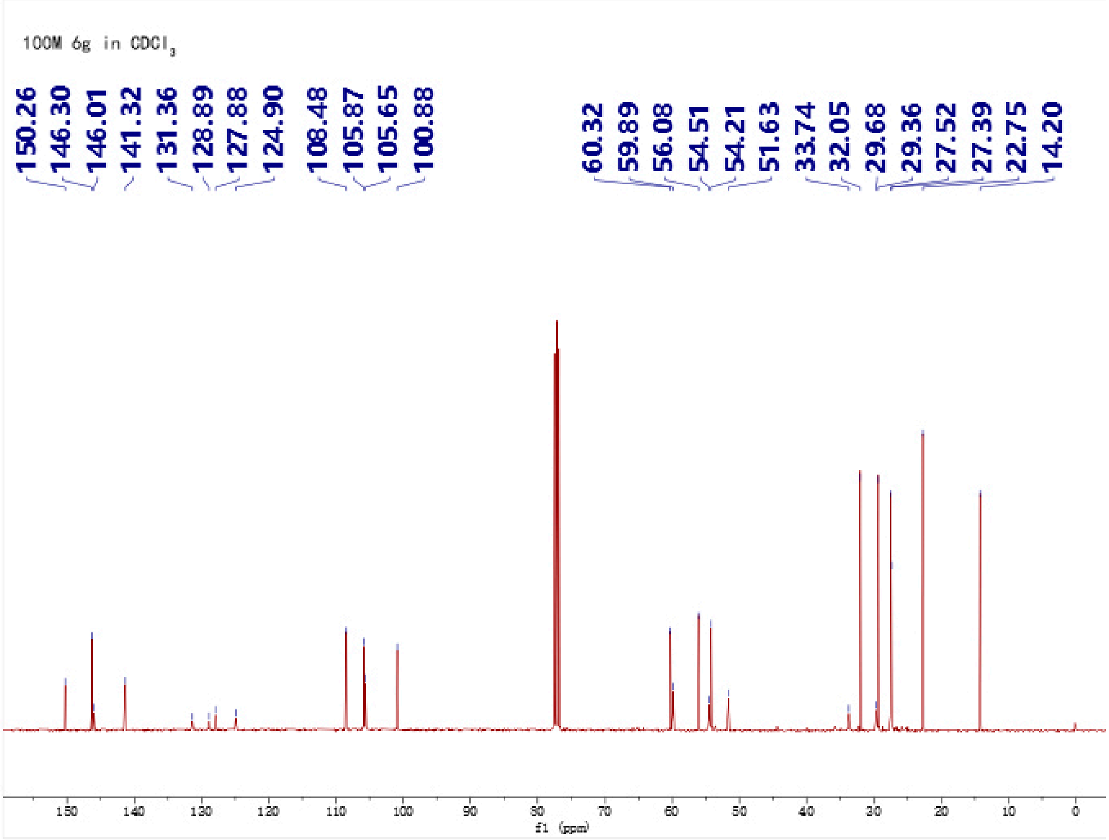

Figure S45. ^{13}C-NMR spectrum of compound **6g**

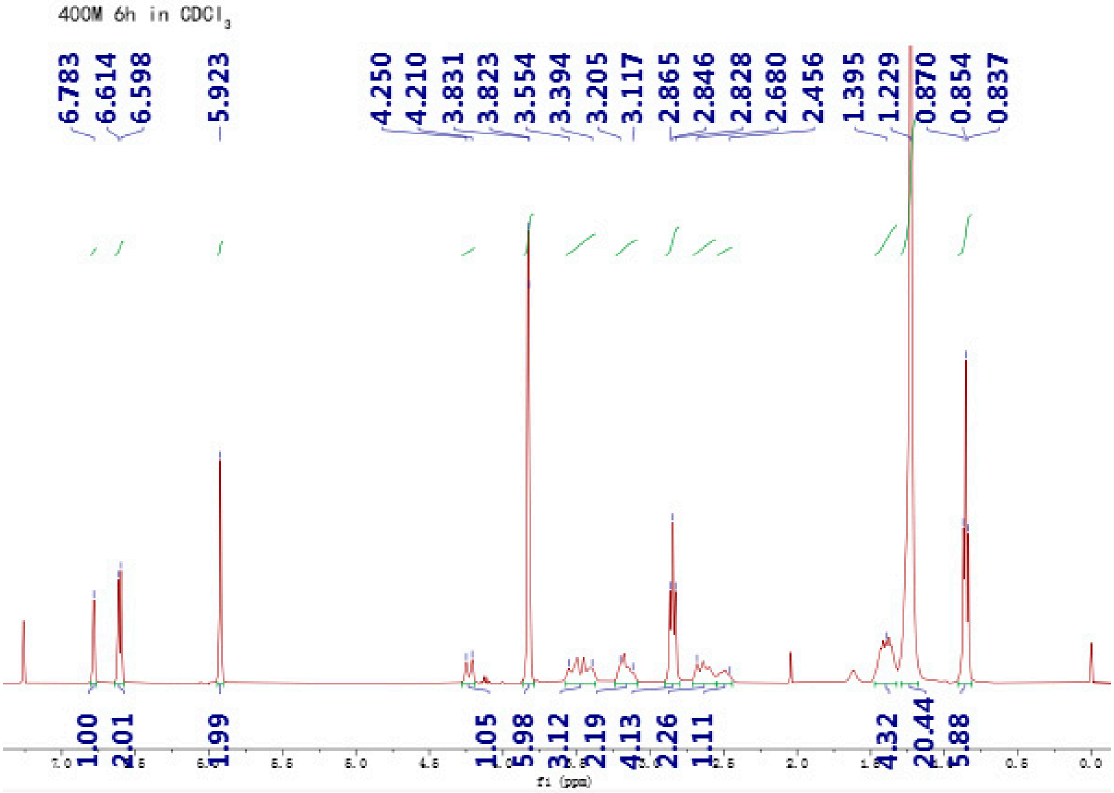

Figure S46. ^{1}H-NMR spectrum of compound **6h**

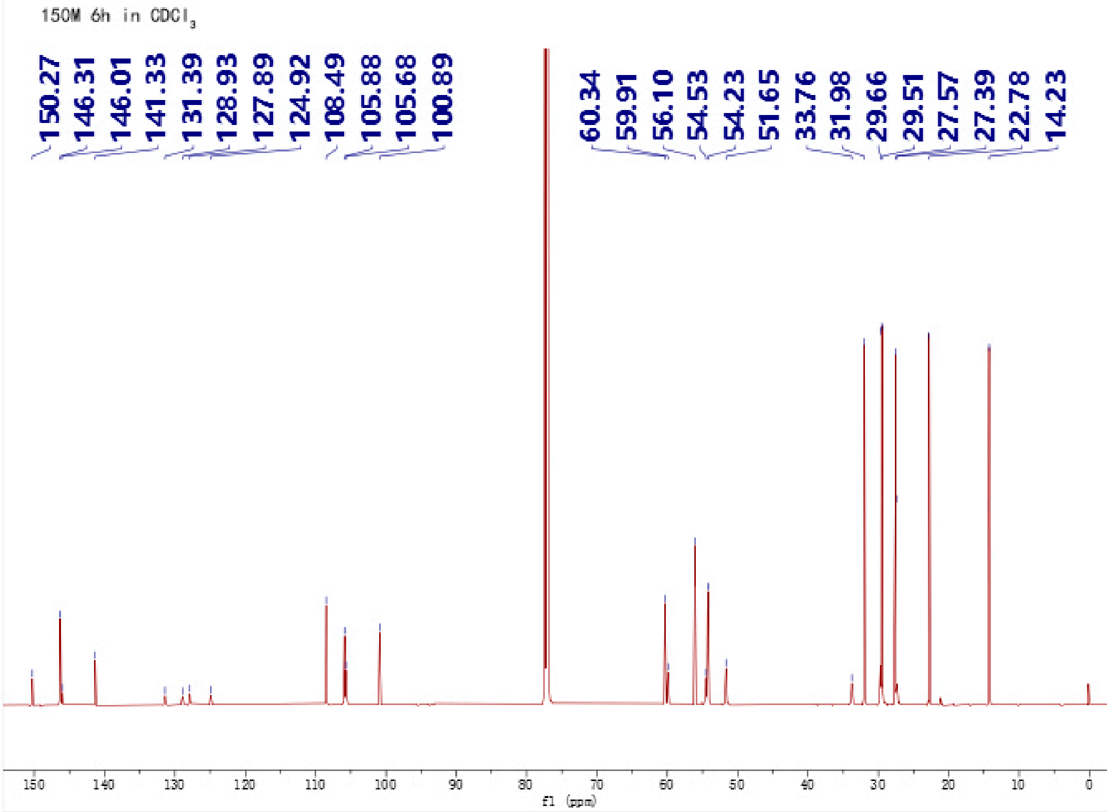

Figure S47. ^{13}C-NMR spectrum of compound **6h**

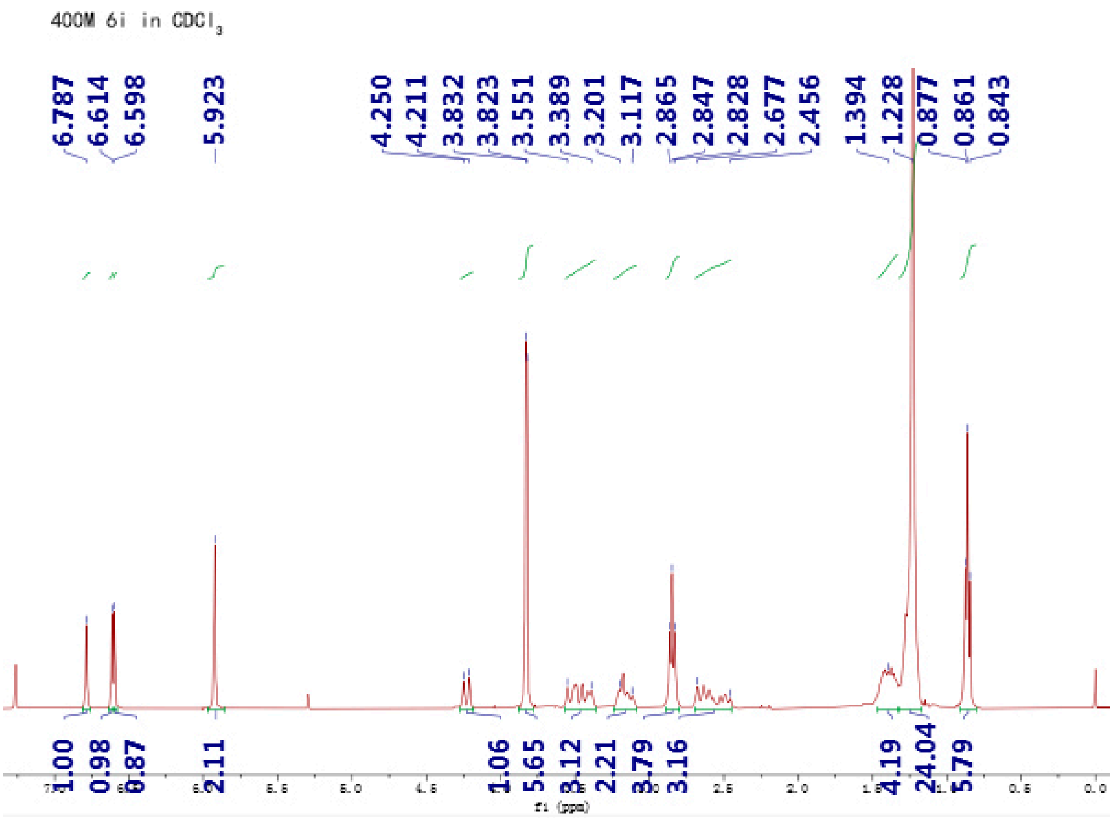

Figure S48. ^{1}H-NMR spectrum of compound 6i

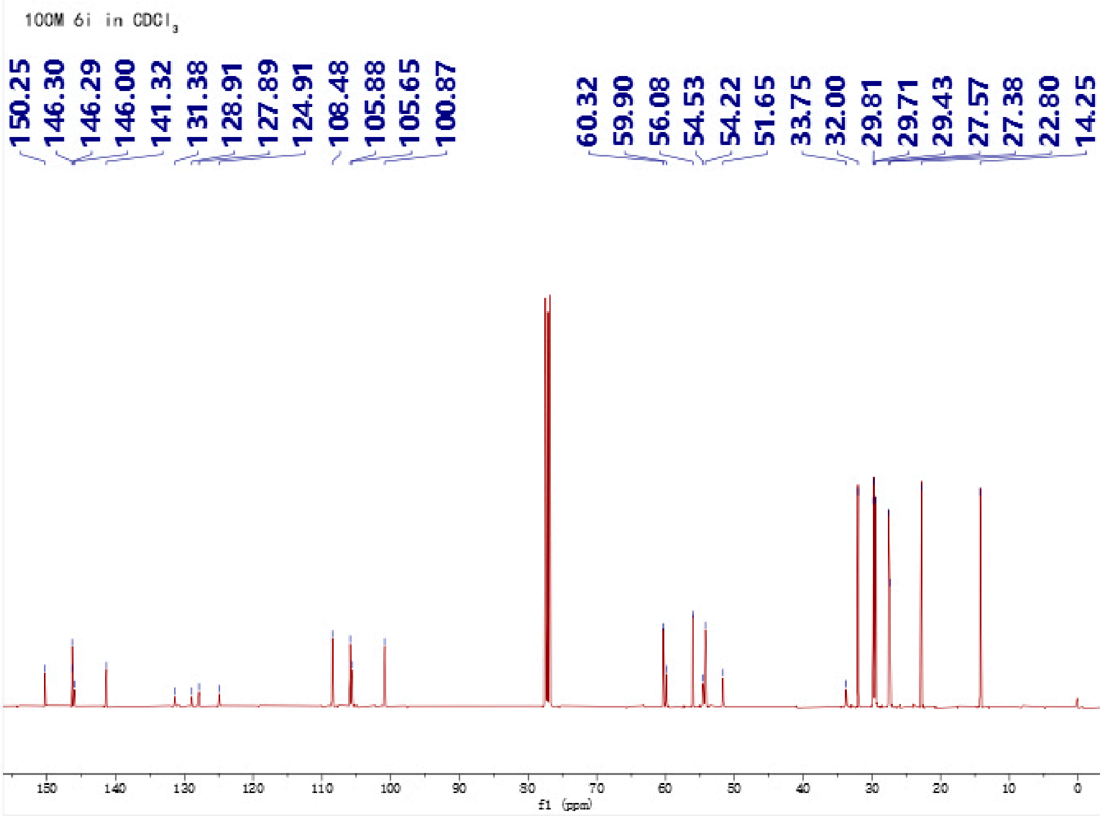

Figure S49. ^{13}C-NMR spectrum of compound **6i**

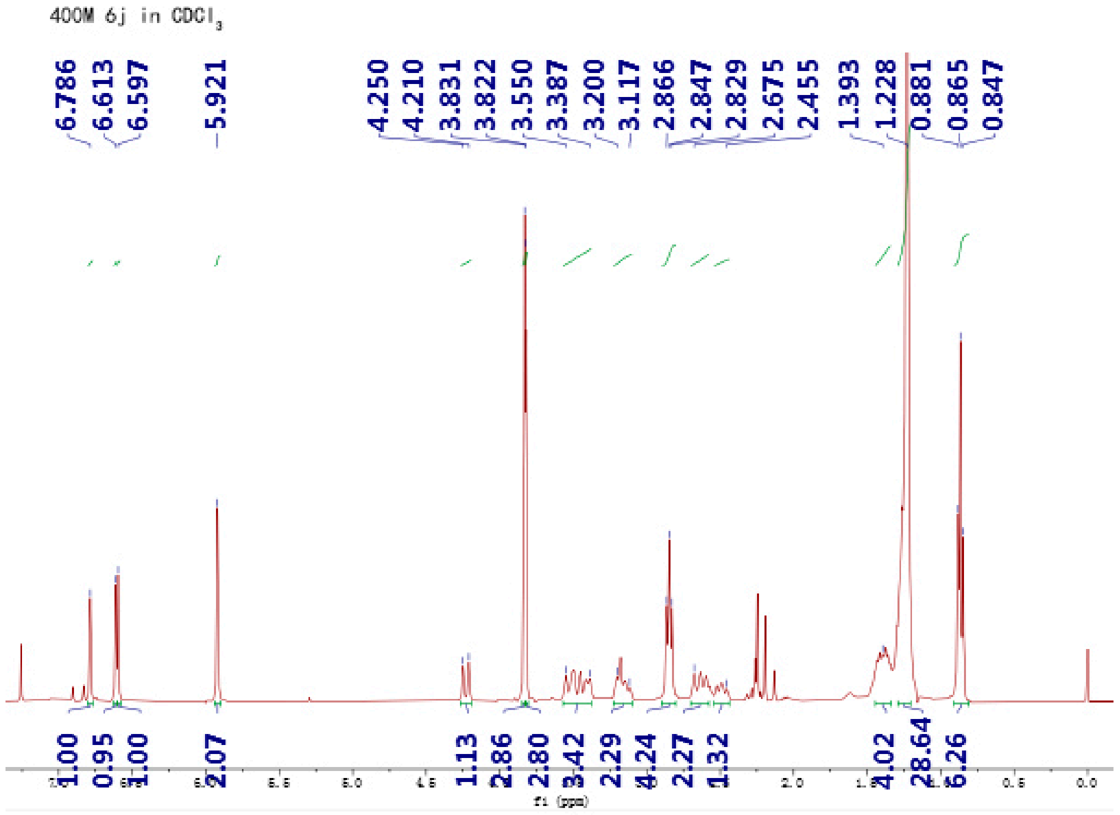

Figure S50. ^{1}H-NMR spectrum of compound **6j**

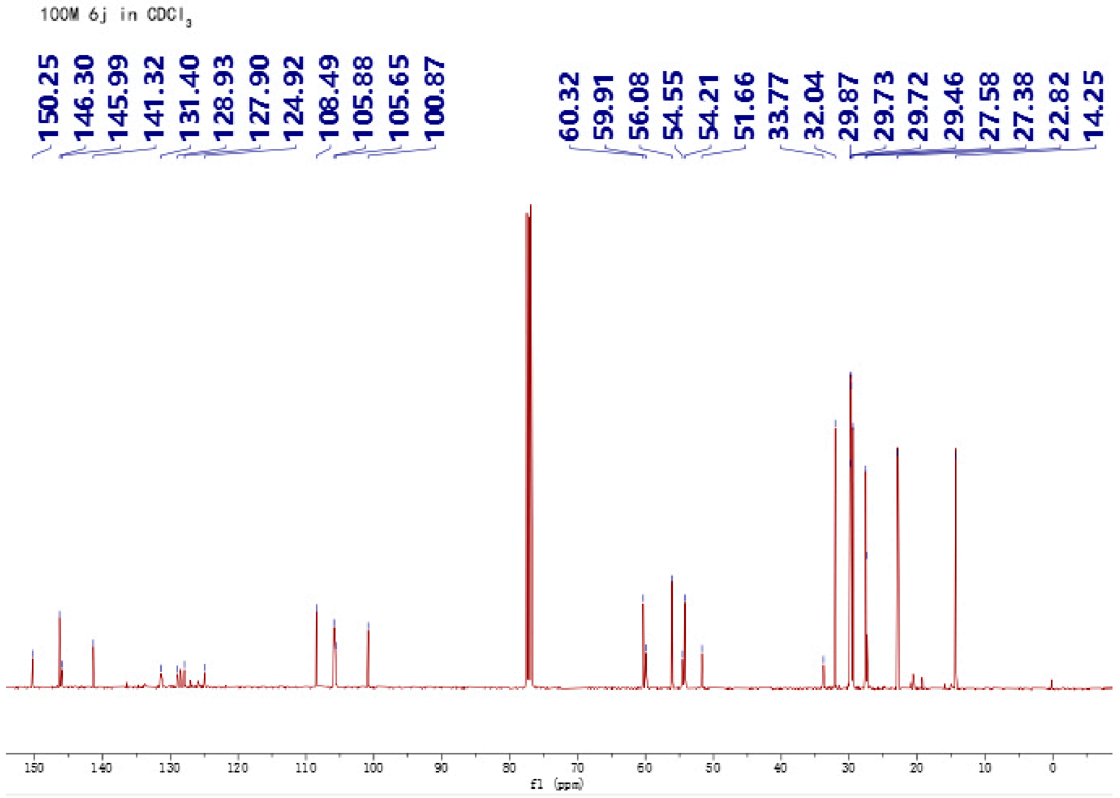

Figure S51. ^{13}C-NMR spectrum of compound **6j**

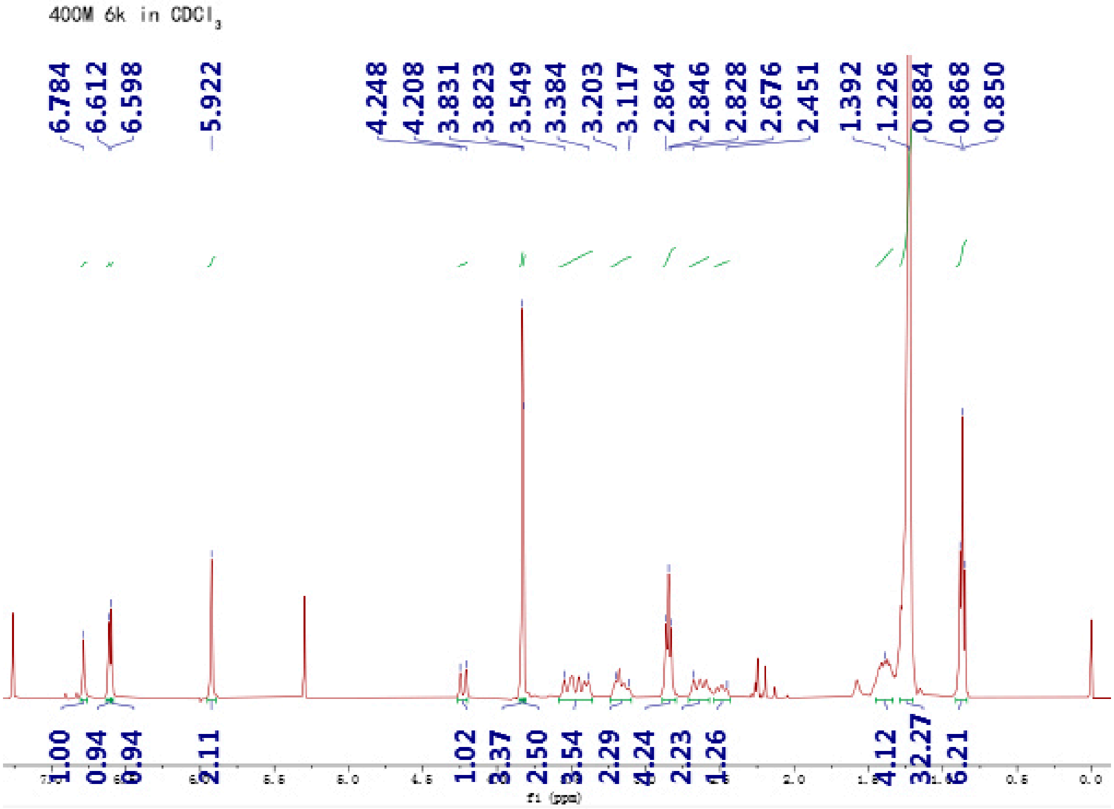

Figure S52. ^{1}H-NMR spectrum of compound **6k**

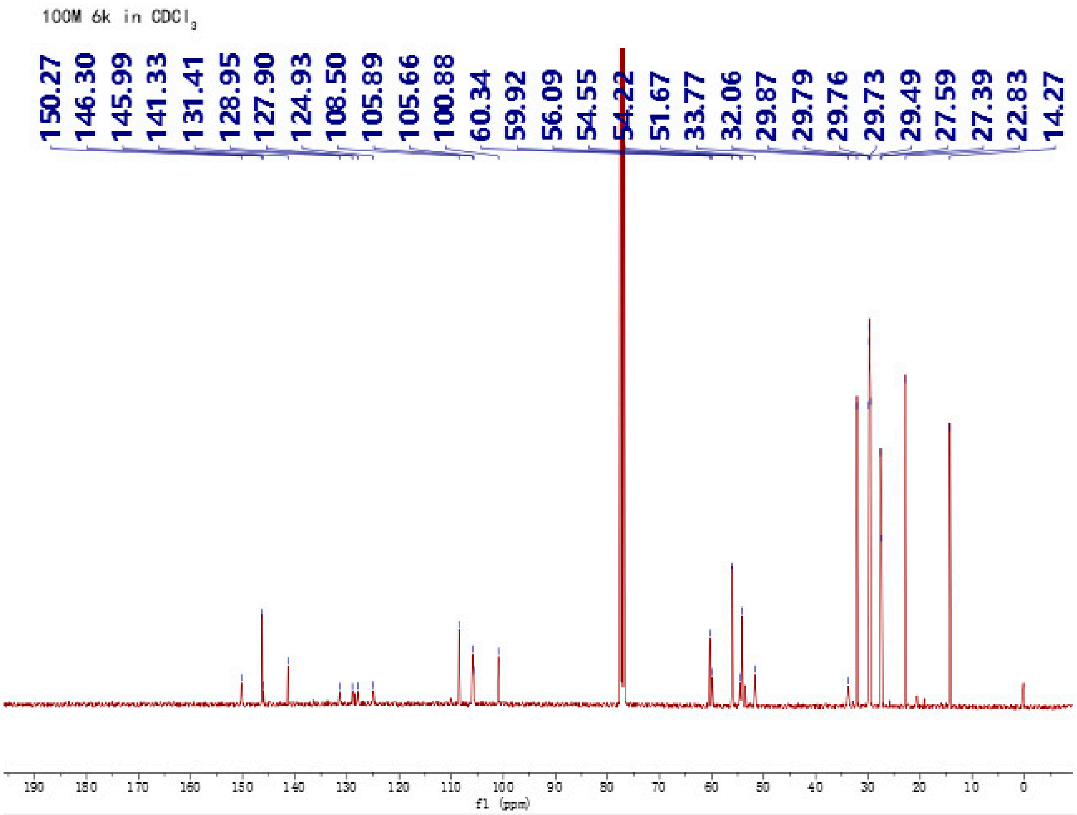

Figure S53. ^{13}C-NMR spectrum of compound **6k**

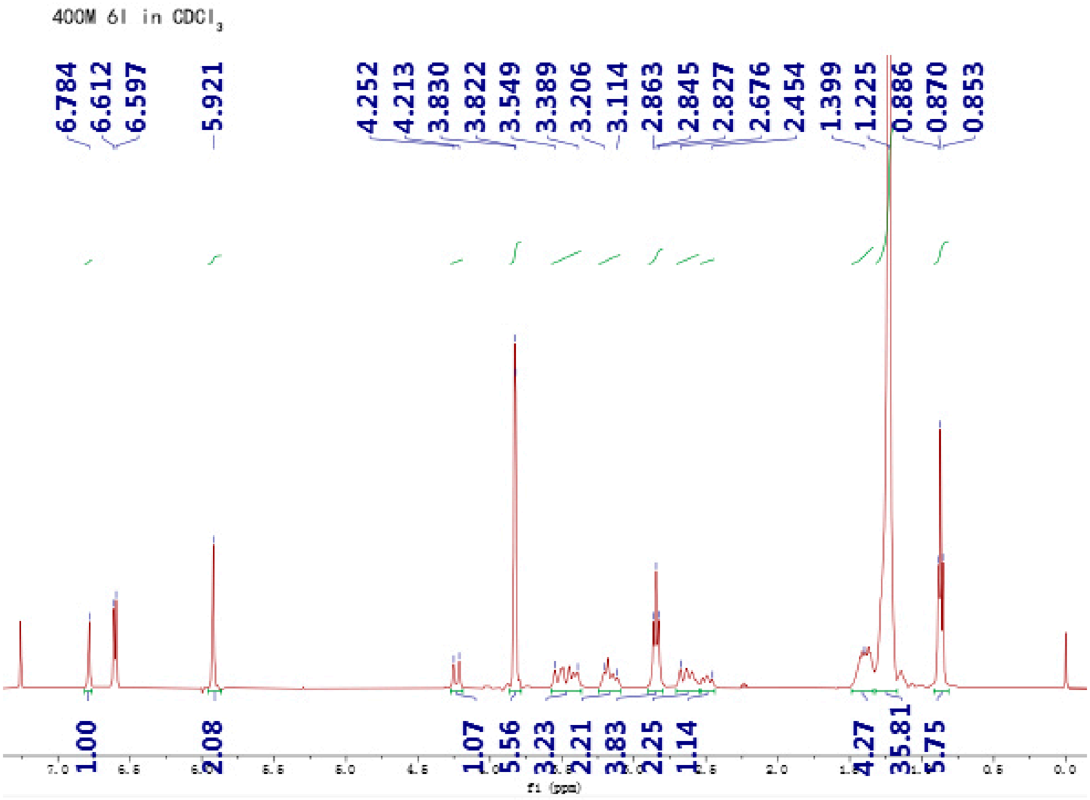

Figure S54. ^{1}H-NMR spectrum of compound **6l**

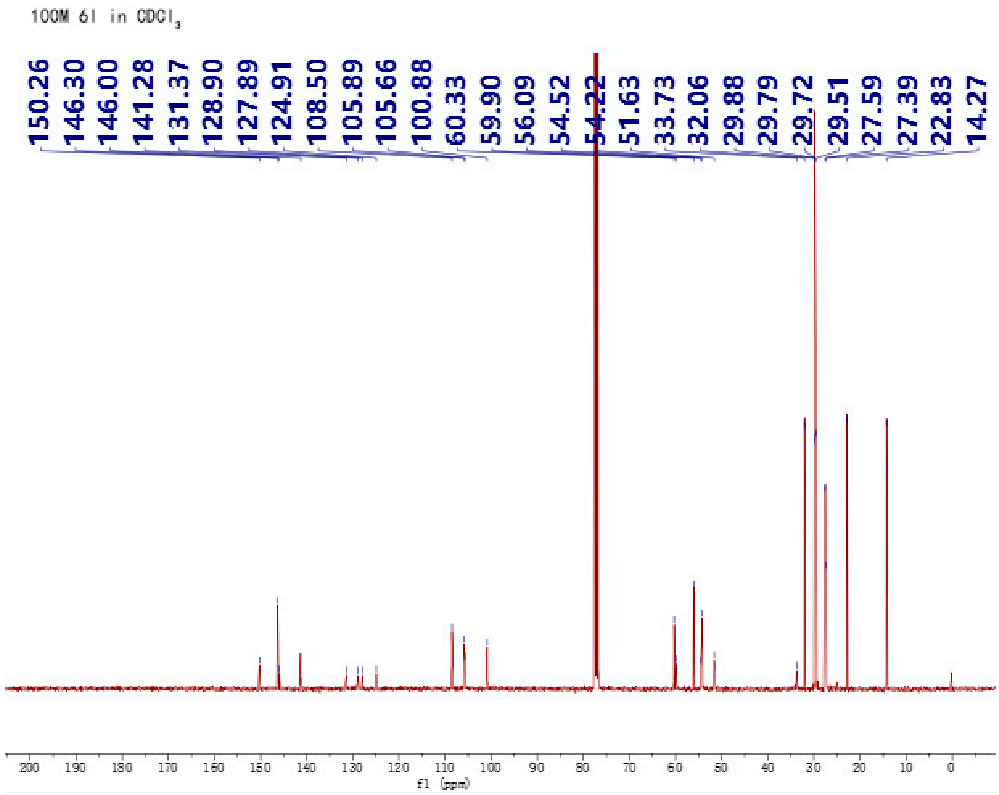

Figure S55. ^{13}C-NMR spectrum of compound **6l**

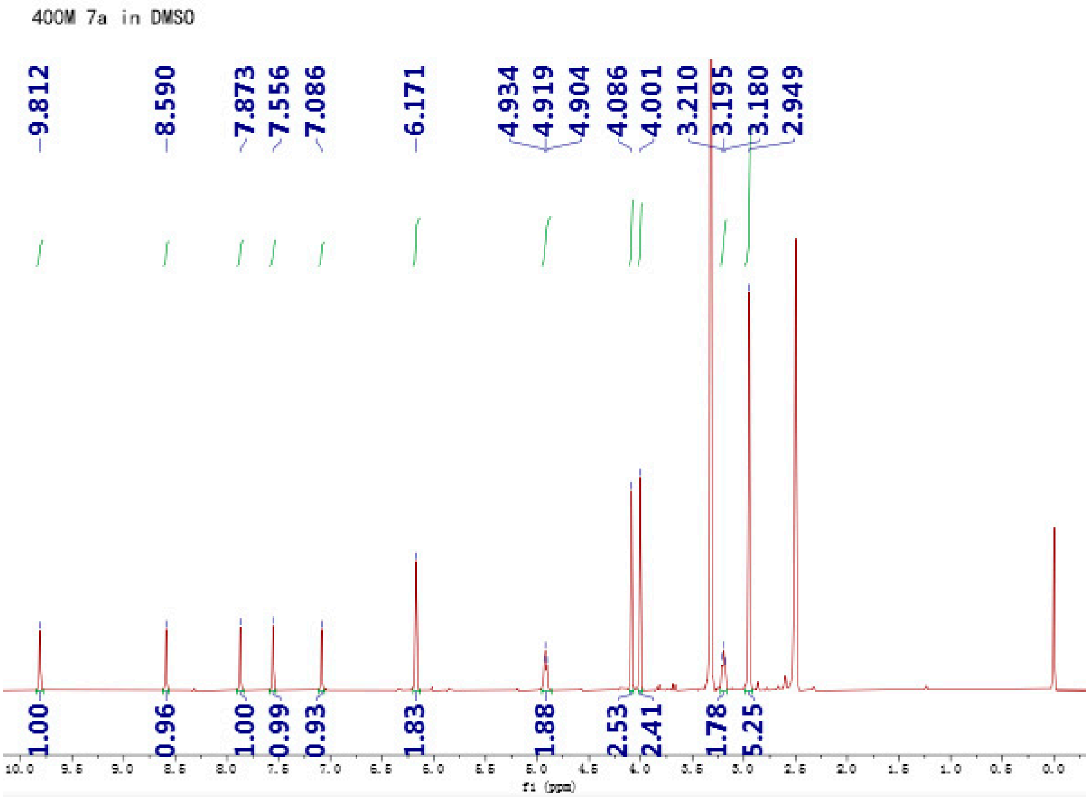

Figure S56. ^{1}H-NMR spectrum of compound **7a**

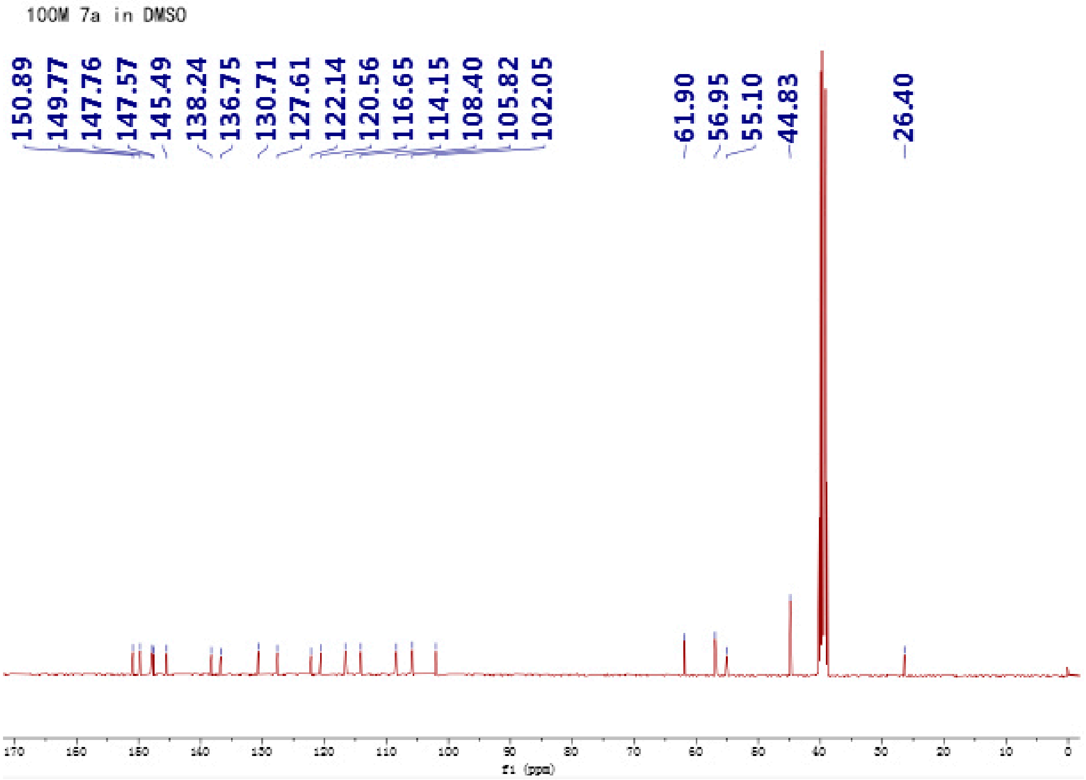

Figure S57. ^{13}C-NMR spectrum of compound **7a**

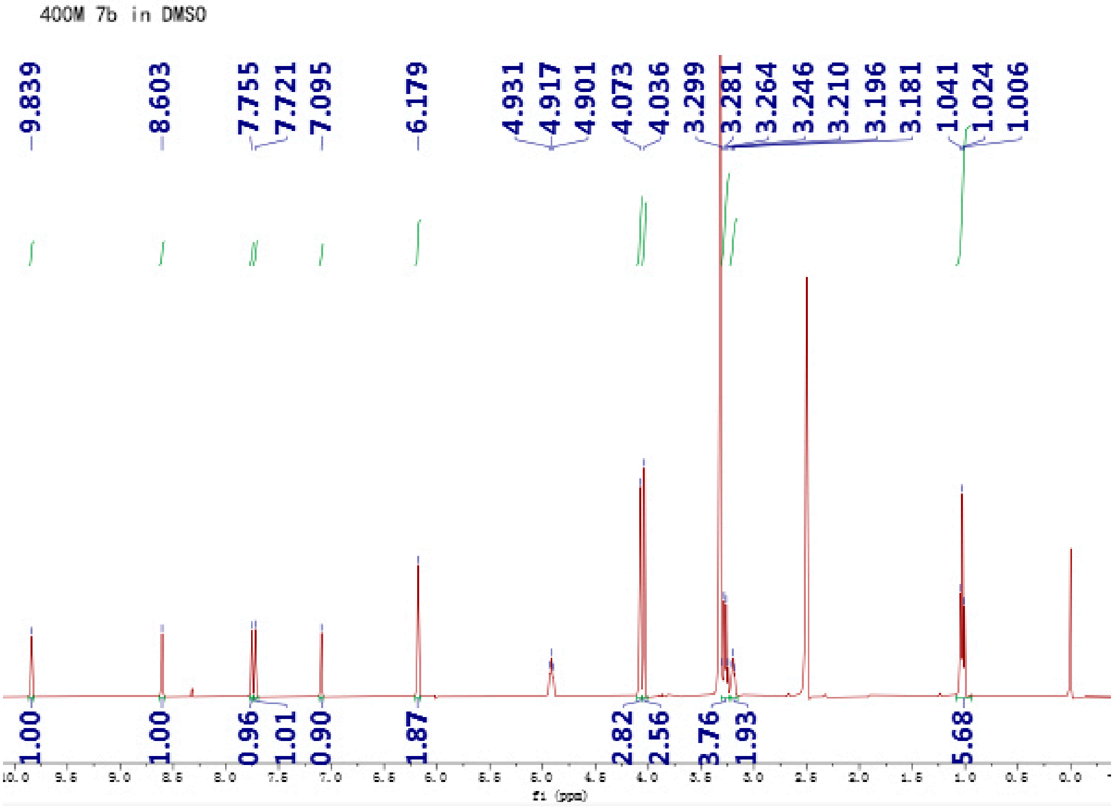

Figure S58. ^{1}H-NMR spectrum of compound **7b**

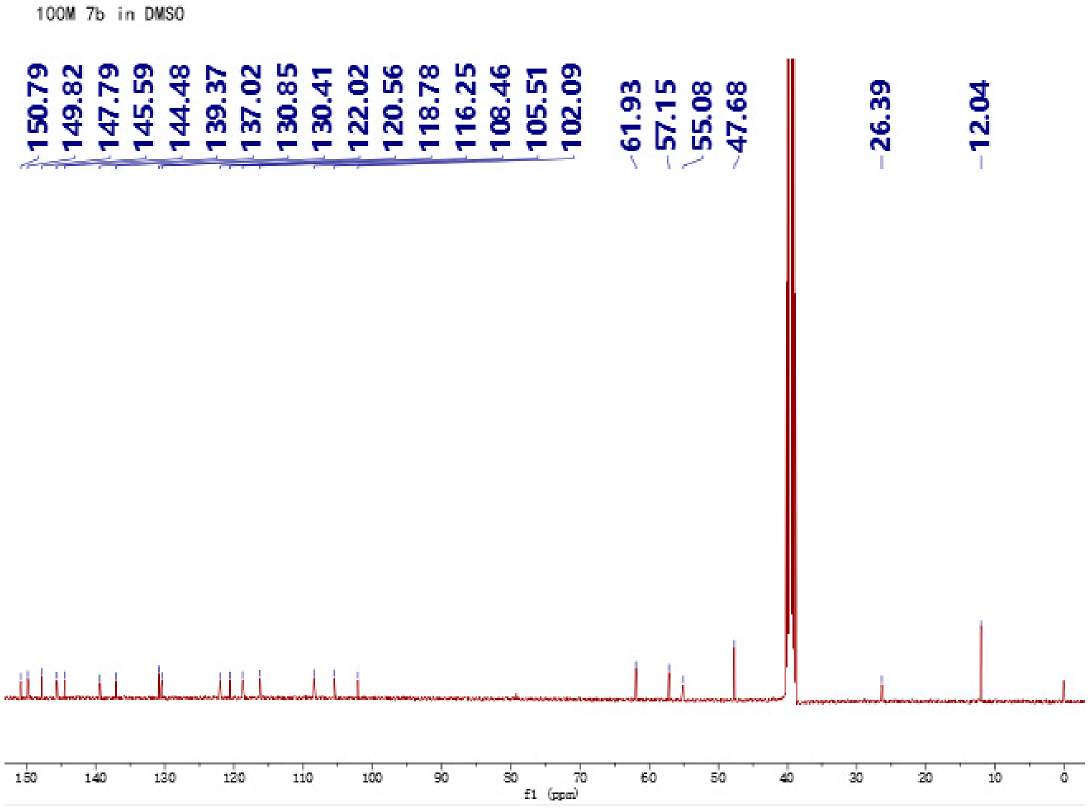

Figure S59. ^{13}C-NMR spectrum of compound **7b**

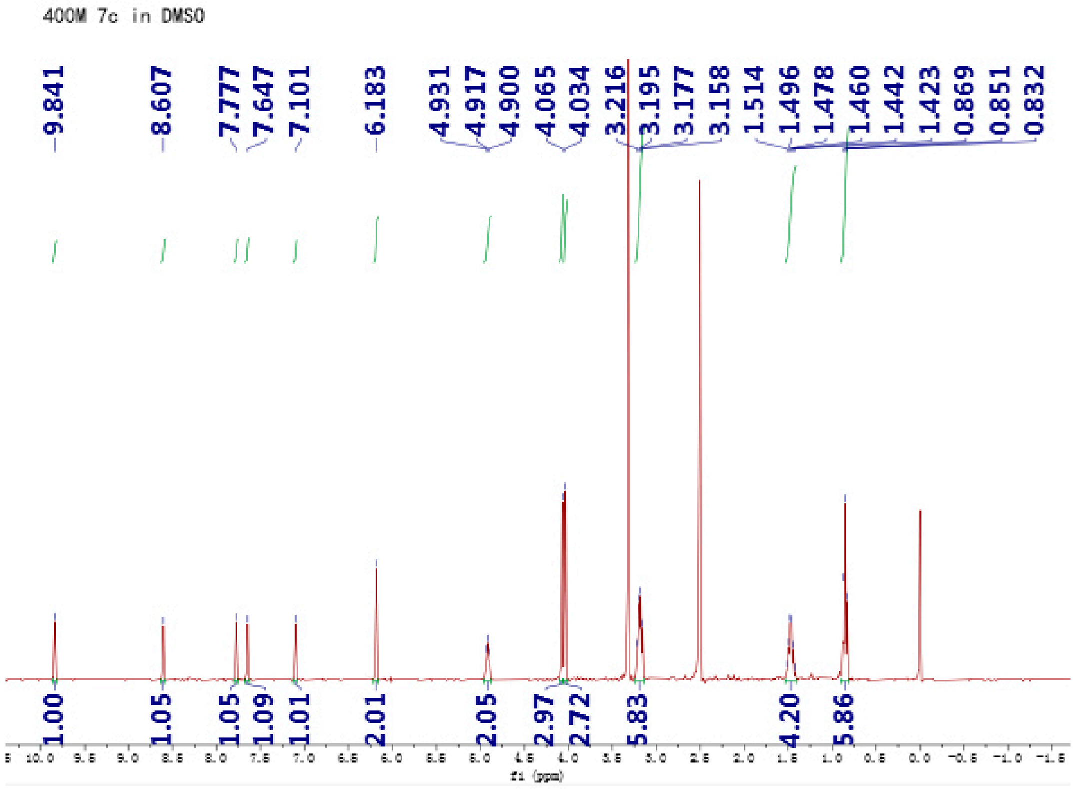

Figure S60. ^{1}H-NMR spectrum of compound **7c**

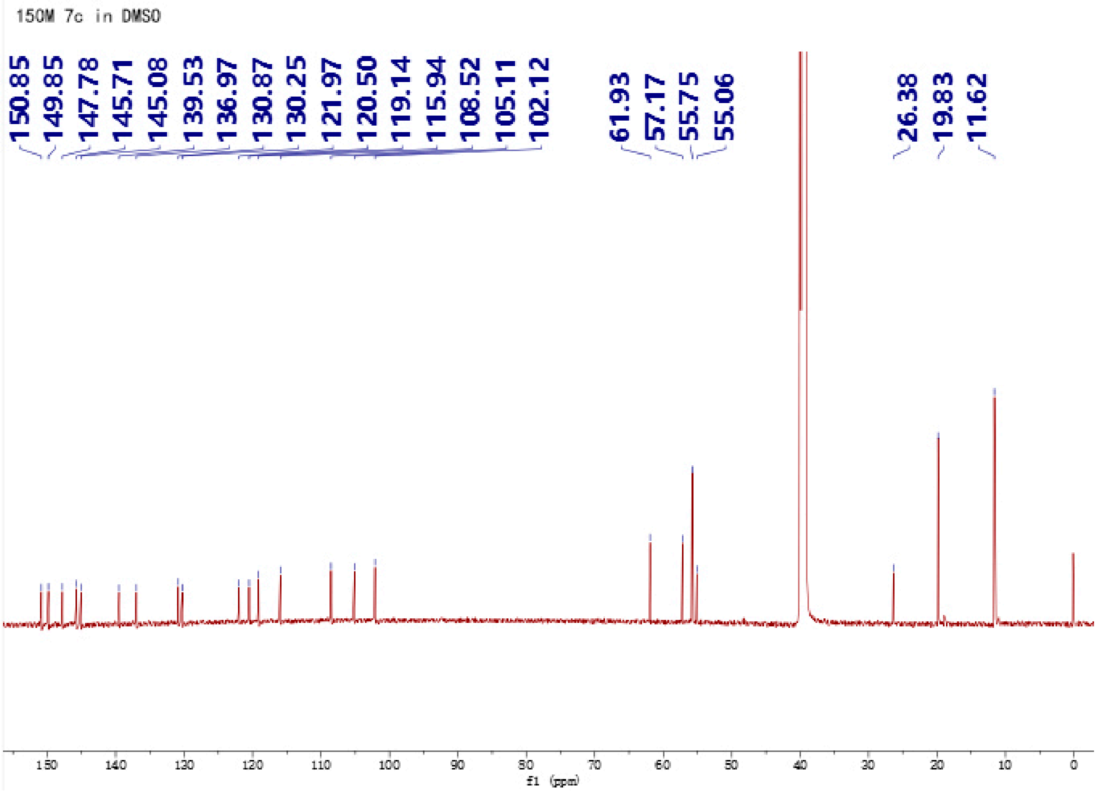

Figure S61. ^{13}C-NMR spectrum of compound **7c**

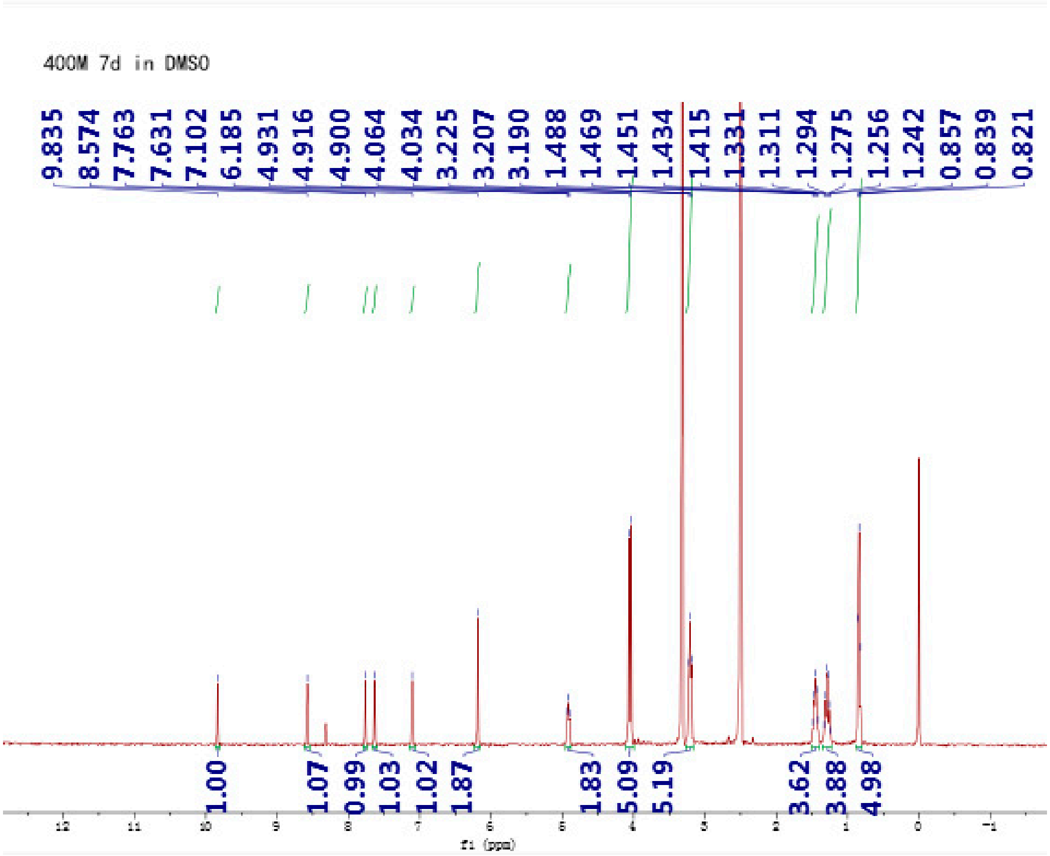

Figure S62. ^{1}H-NMR spectrum of compound **7d**

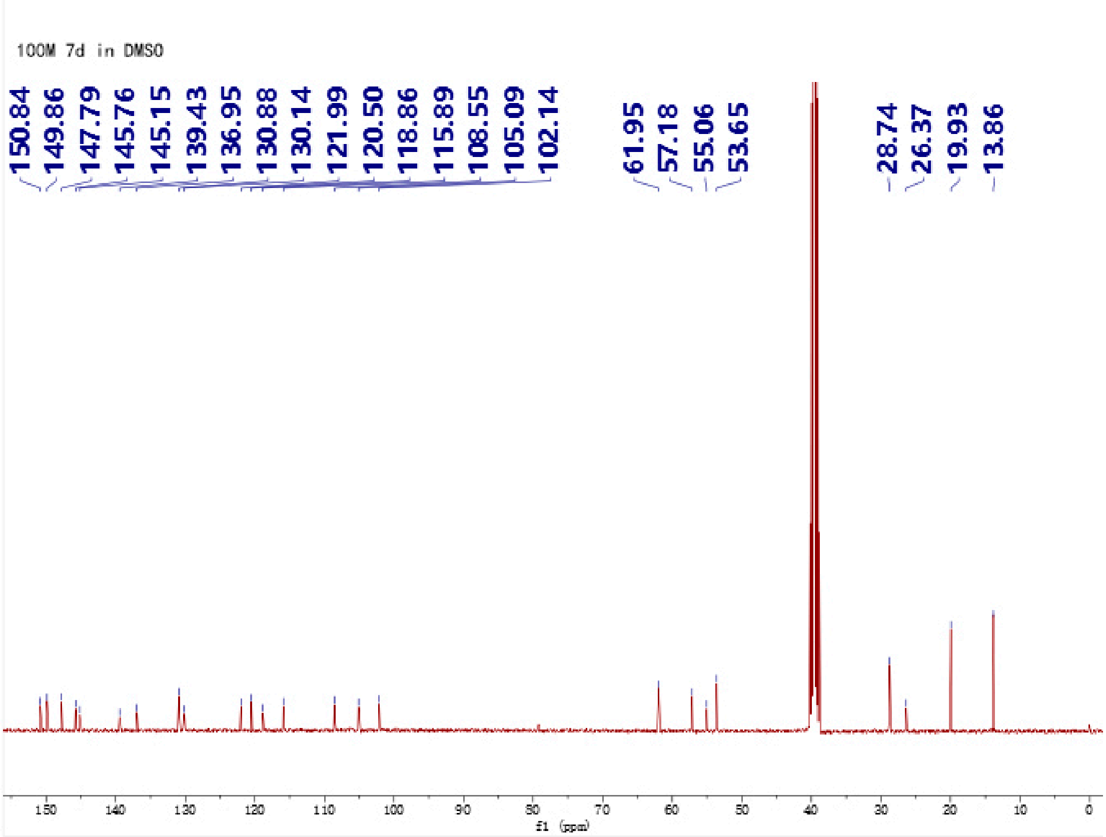

Figure S63. ^{13}C-NMR spectrum of compound **7d**

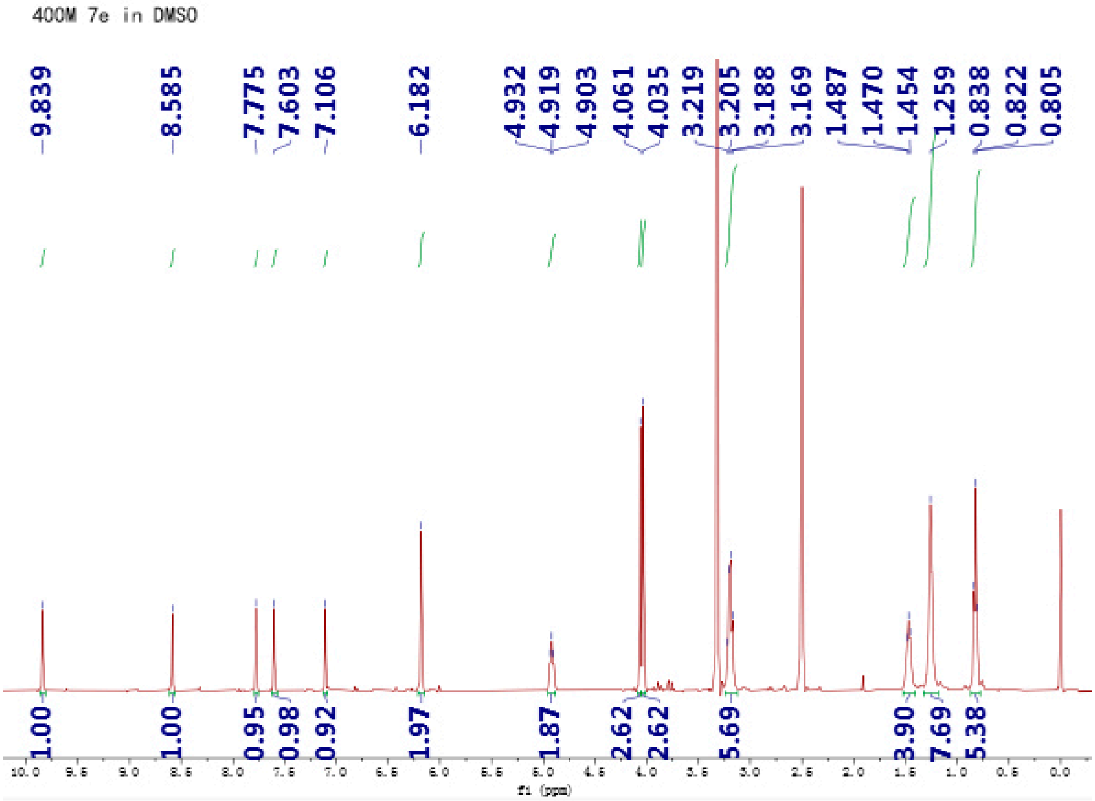

Figure S64. ^{1}H-NMR spectrum of compound **7e**

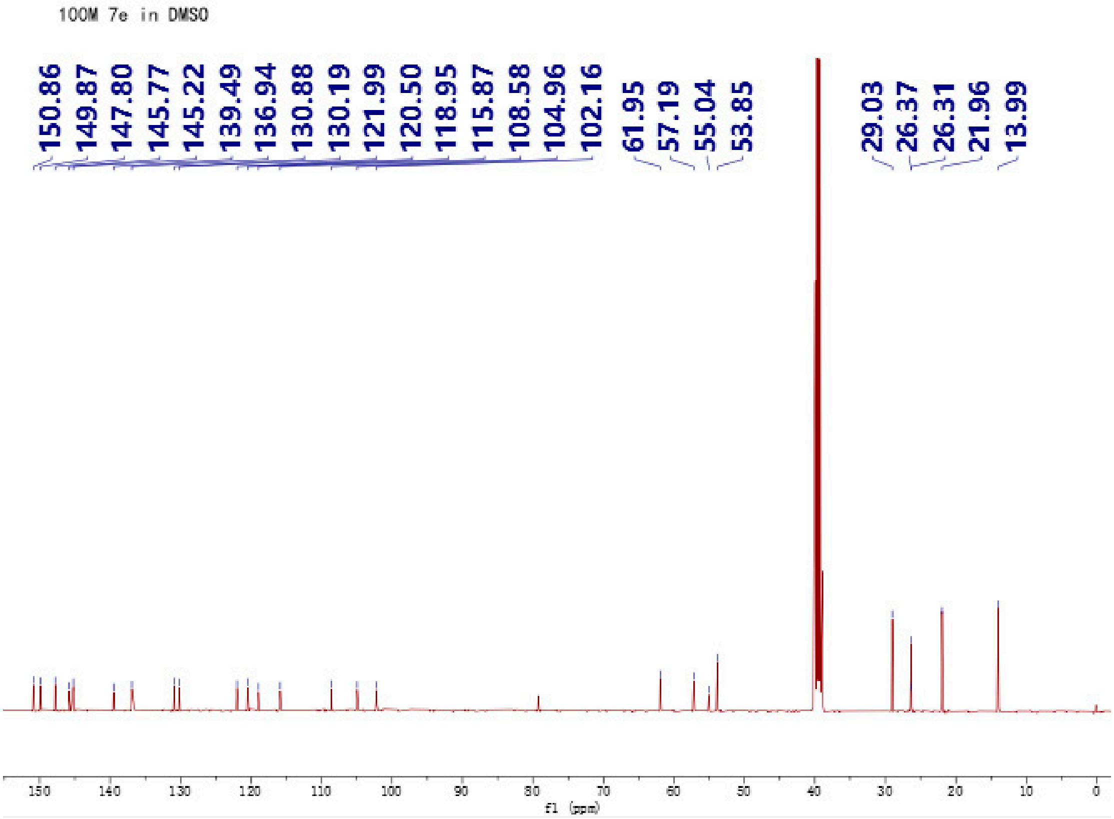

Figure S65. ^{13}C-NMR spectrum of compound **7e**

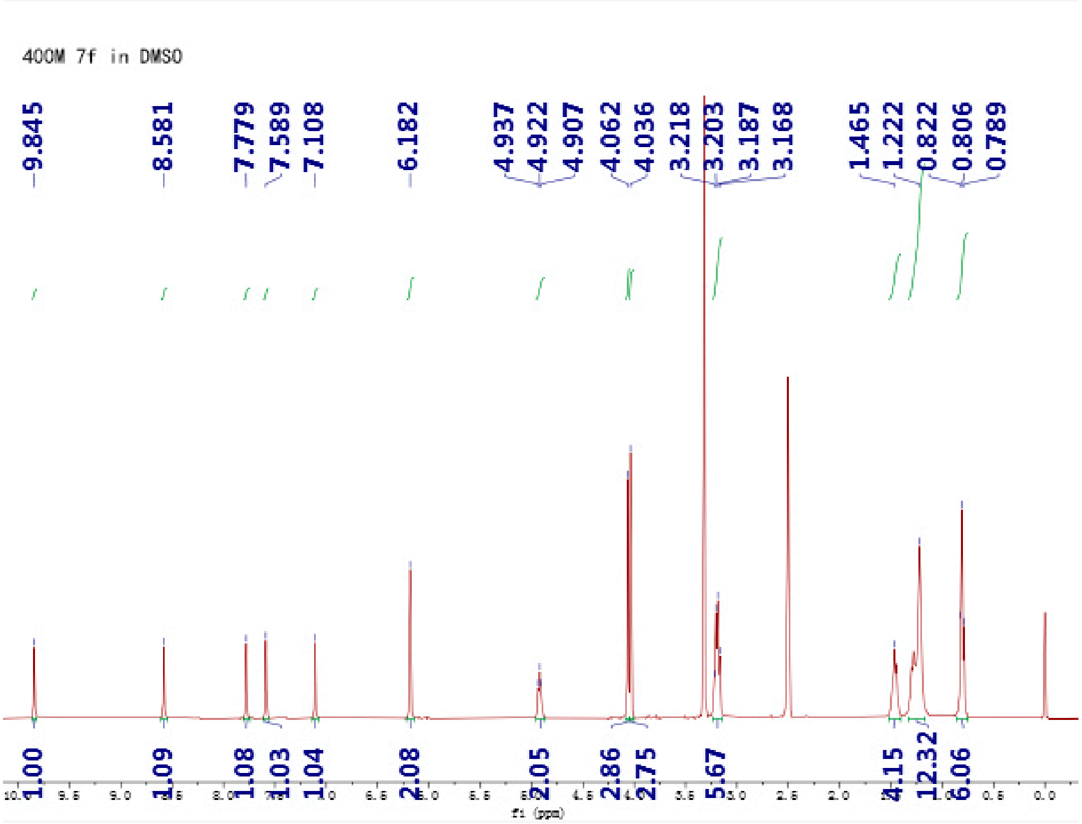

Figure S66. ^{1}H-NMR spectrum of compound **7f**

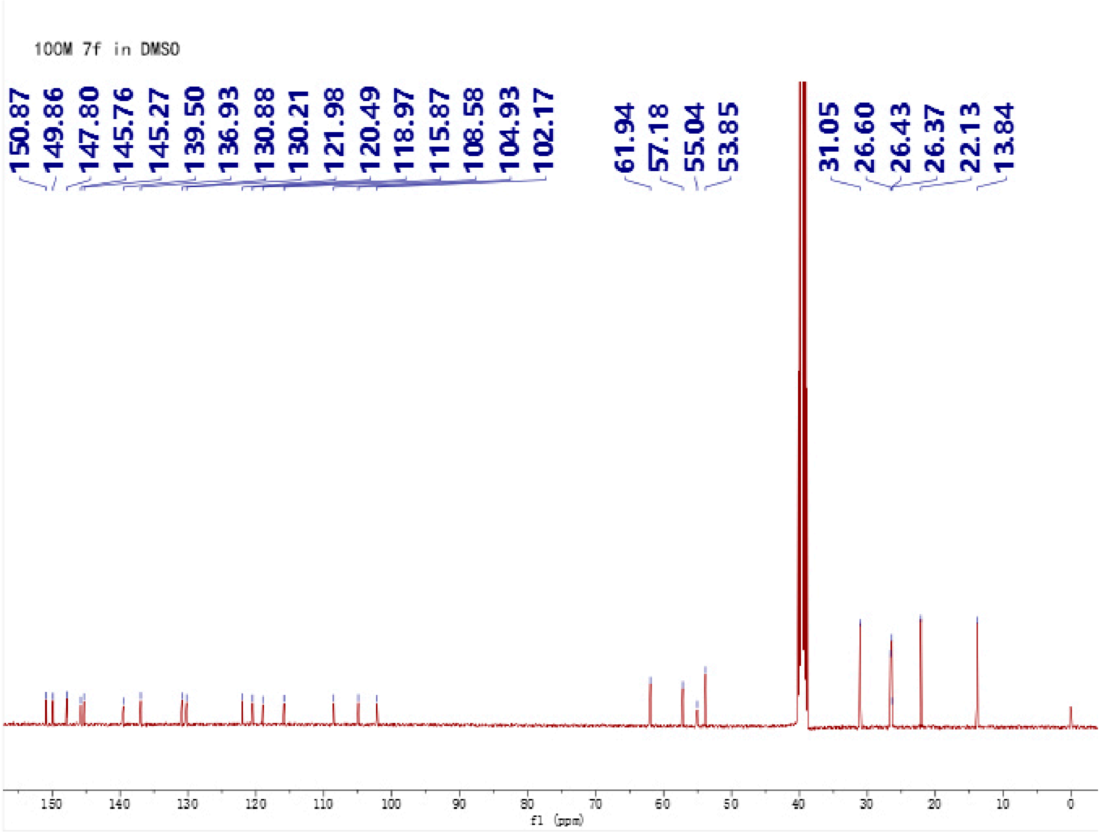

Figure S67. ^{13}C-NMR spectrum of compound **7f**

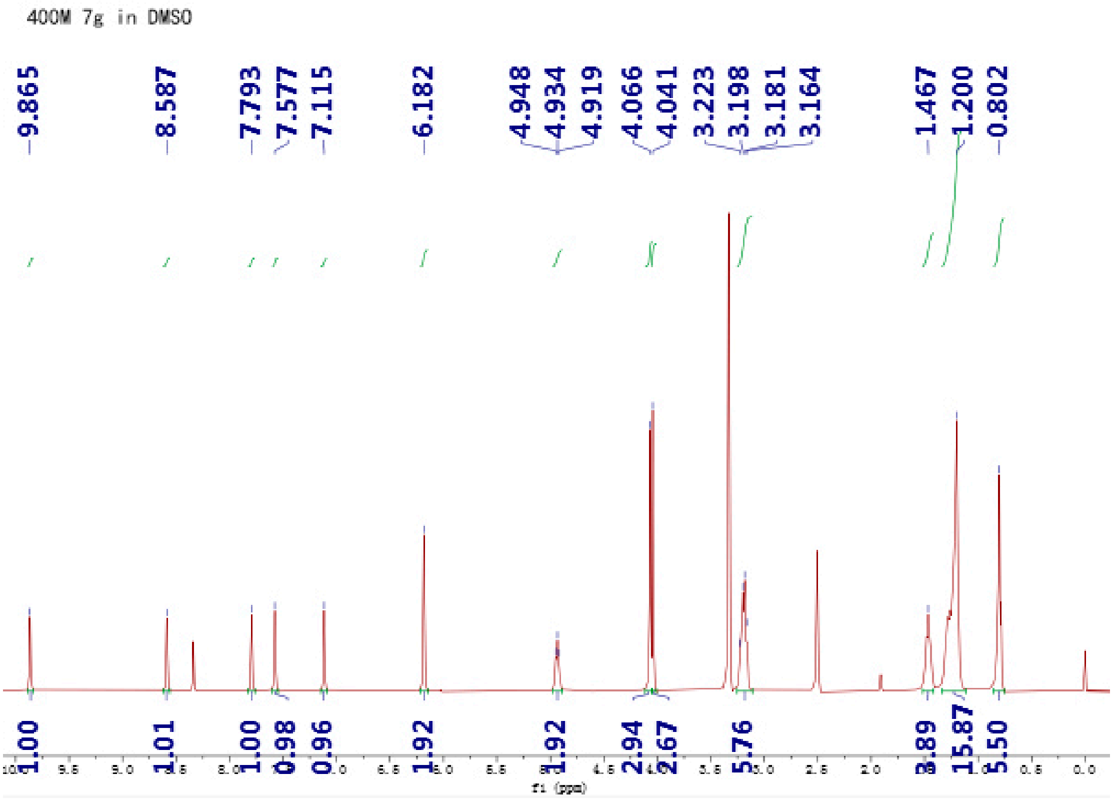

Figure S68. ^{1}H-NMR spectrum of compound **7g**

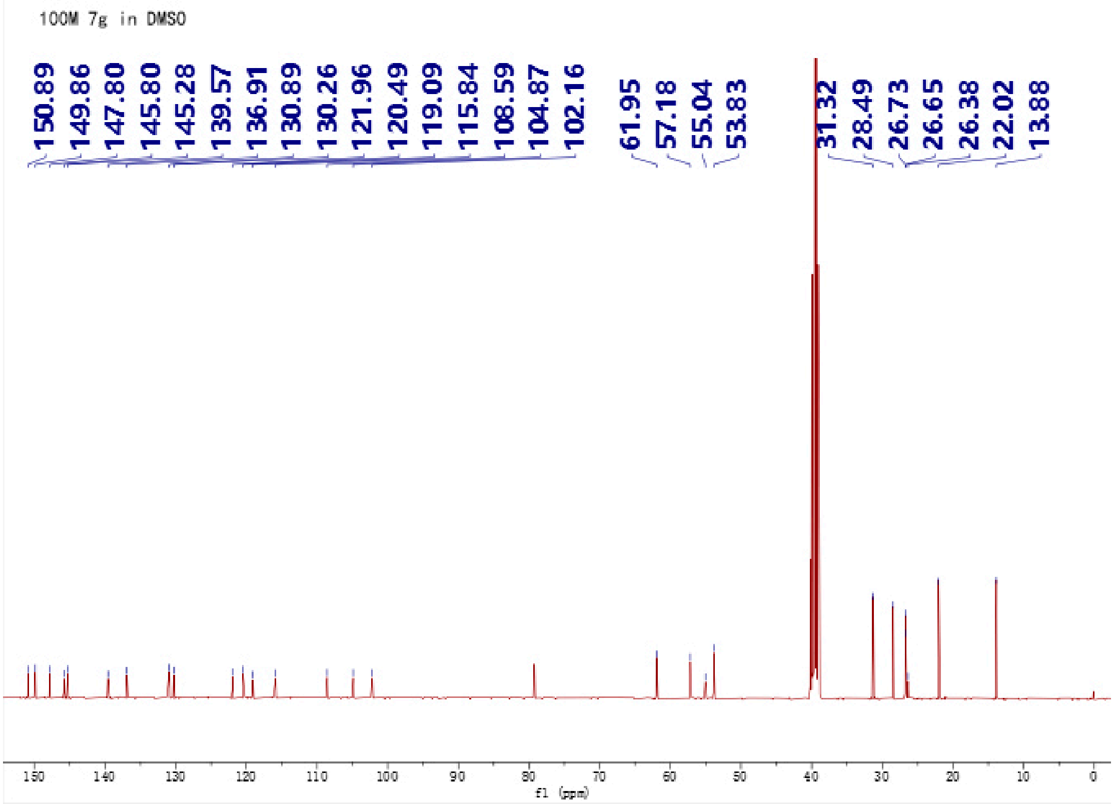

Figure S69. ^{13}C-NMR spectrum of compound **7g**

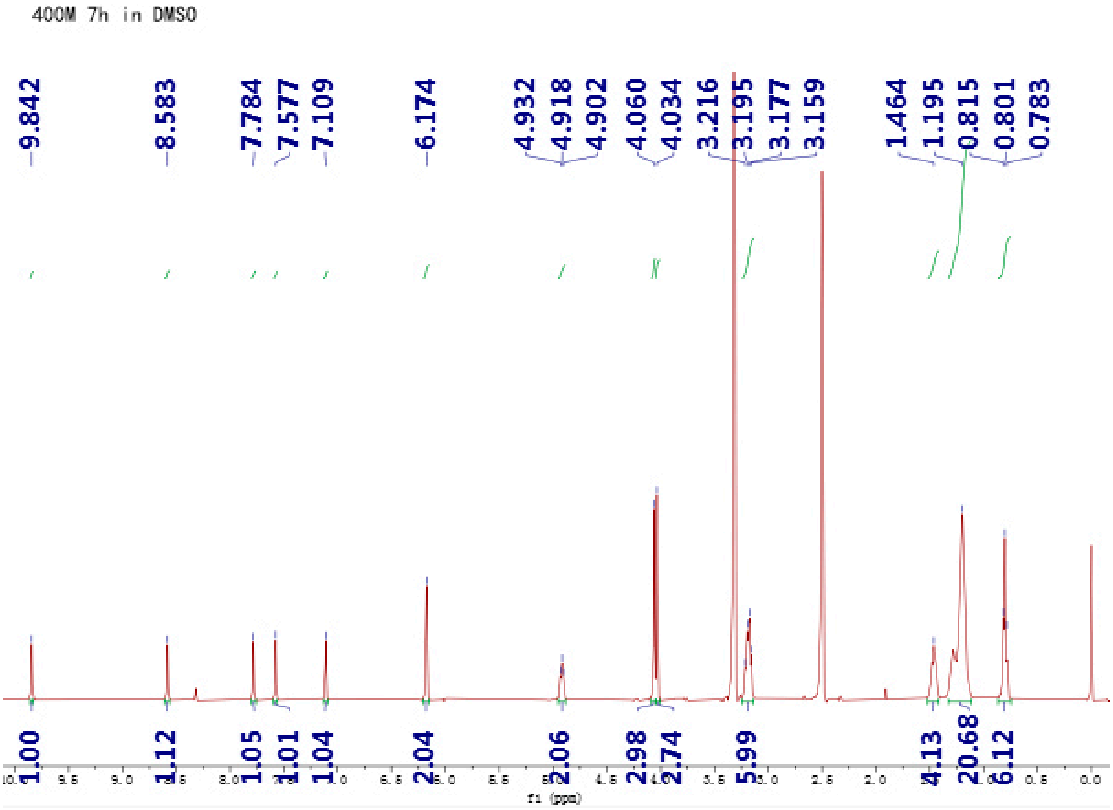

Figure S70. ^{1}H-NMR spectrum of compound **7h**

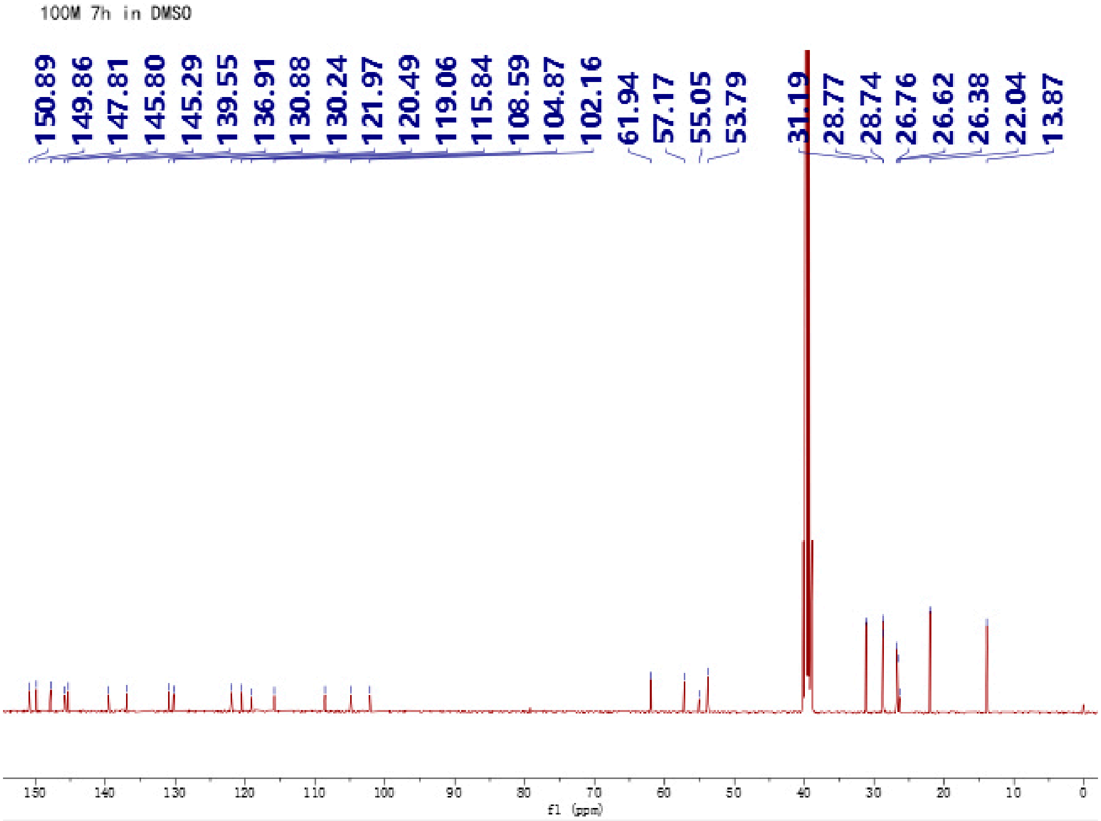

Figure S71. ^{13}C-NMR spectrum of compound **7h**

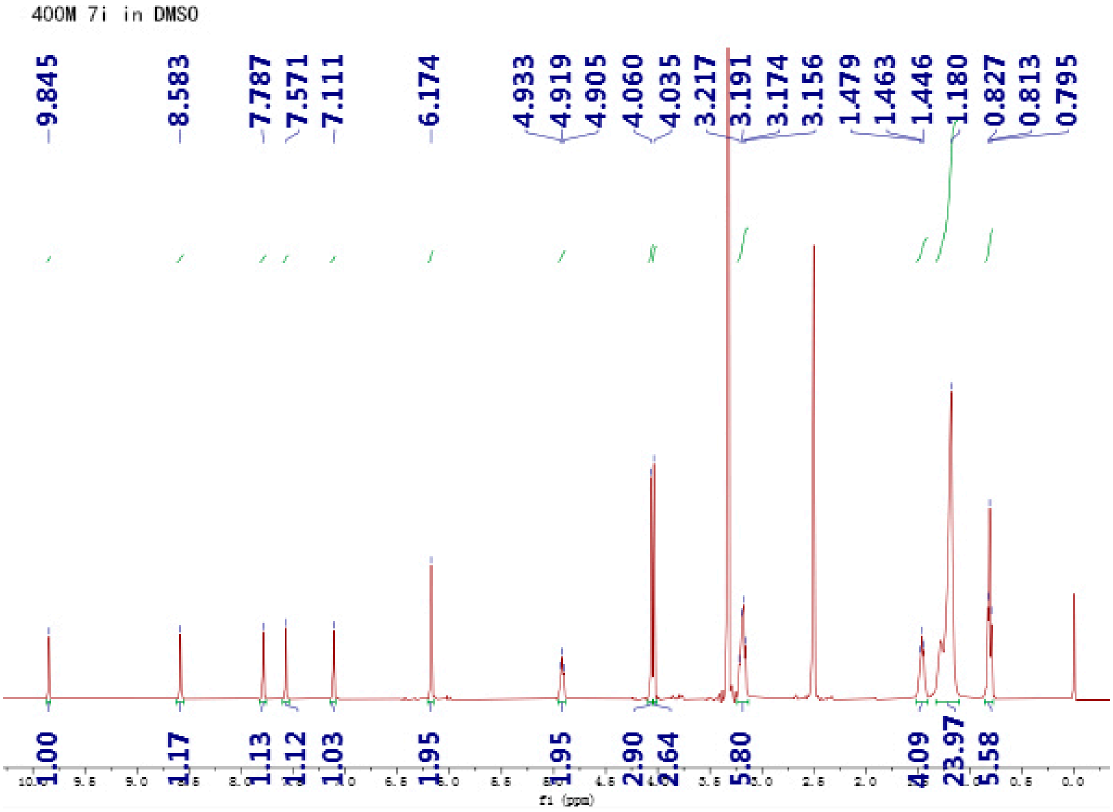

Figure S72. ^{1}H-NMR spectrum of compound **7i**

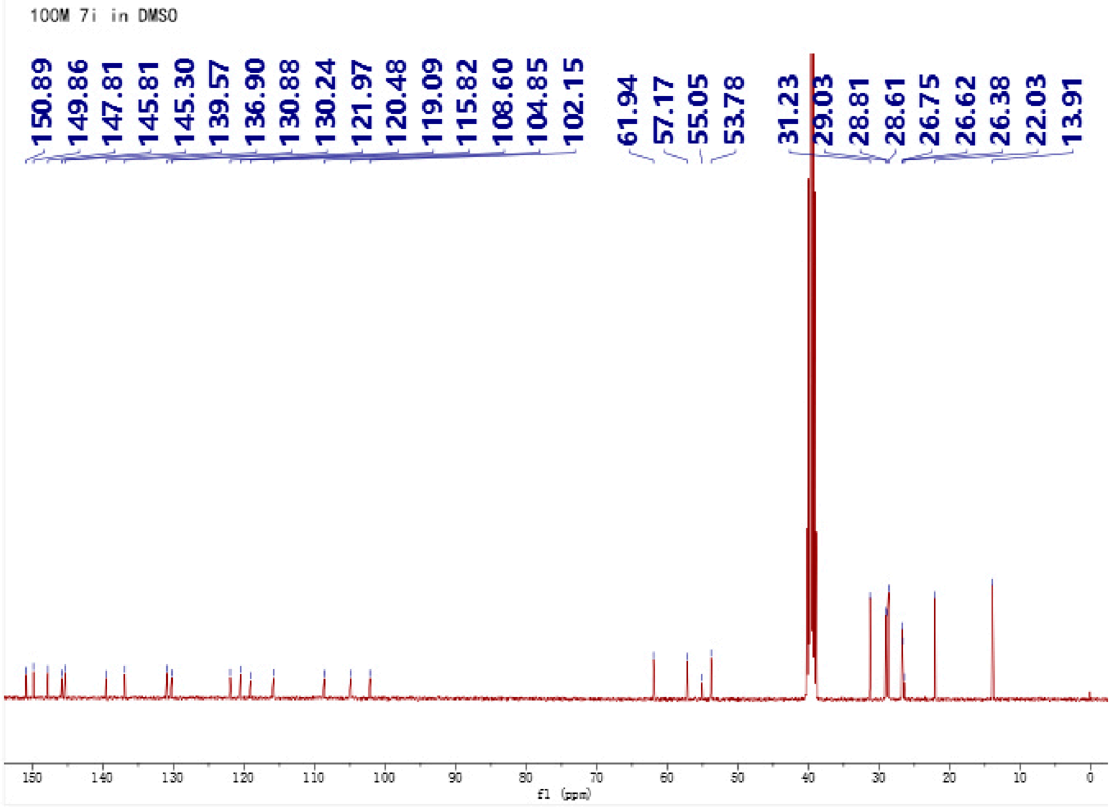

Figure S73. ^{13}C-NMR spectrum of compound **7i**

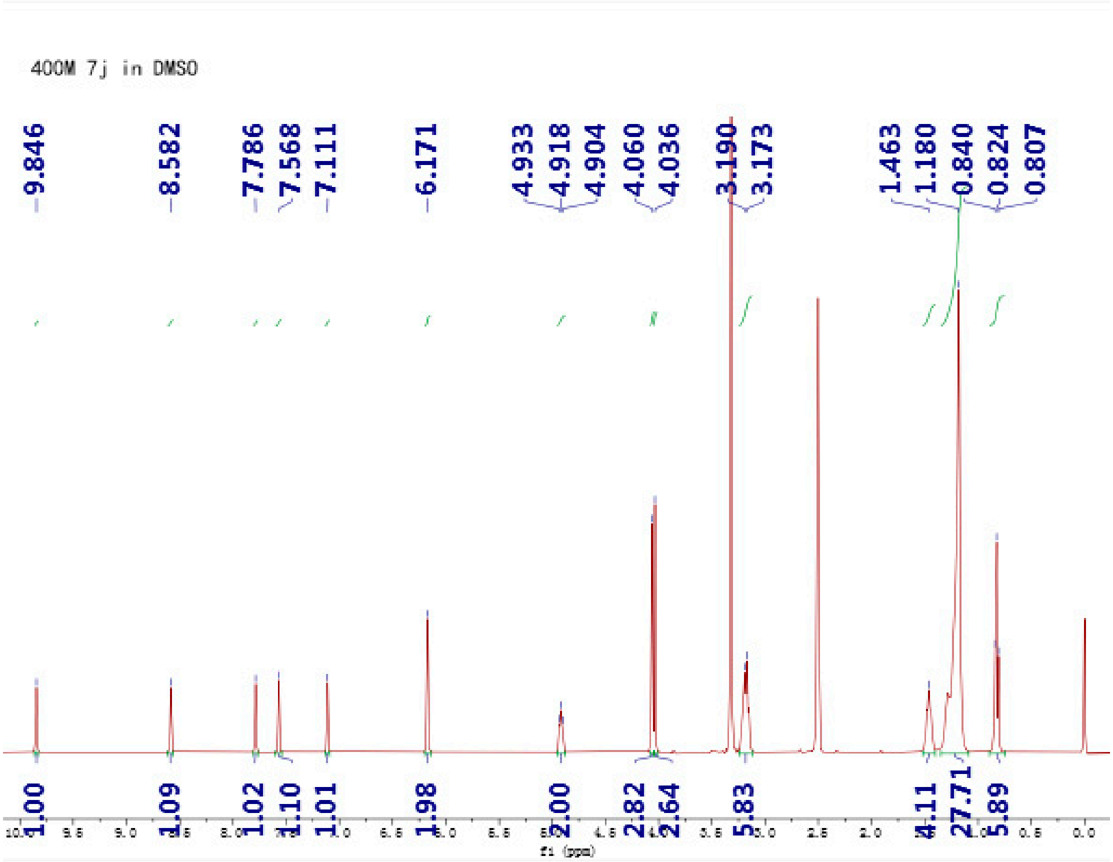

Figure S74. ^{1}H-NMR spectrum of compound **7j**

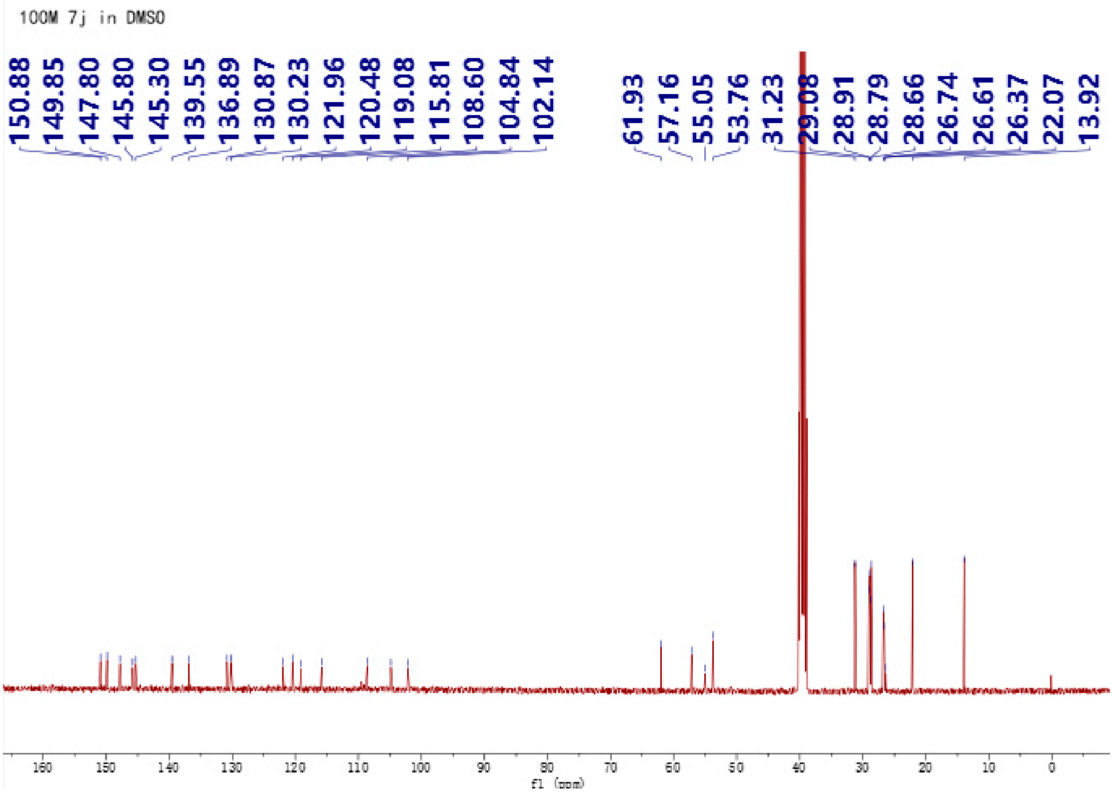

Figure S75. ^{13}C-NMR spectrum of compound **7j**

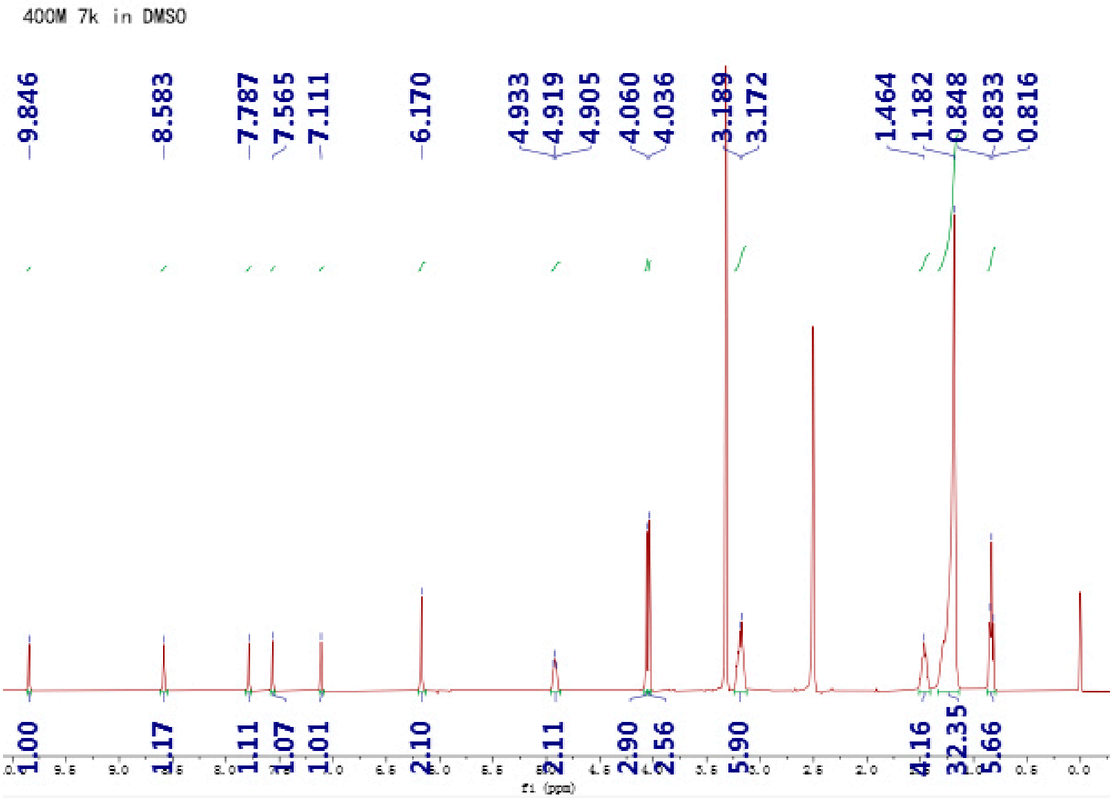

Figure S76. ^{1}H-NMR spectrum of compound **7k**

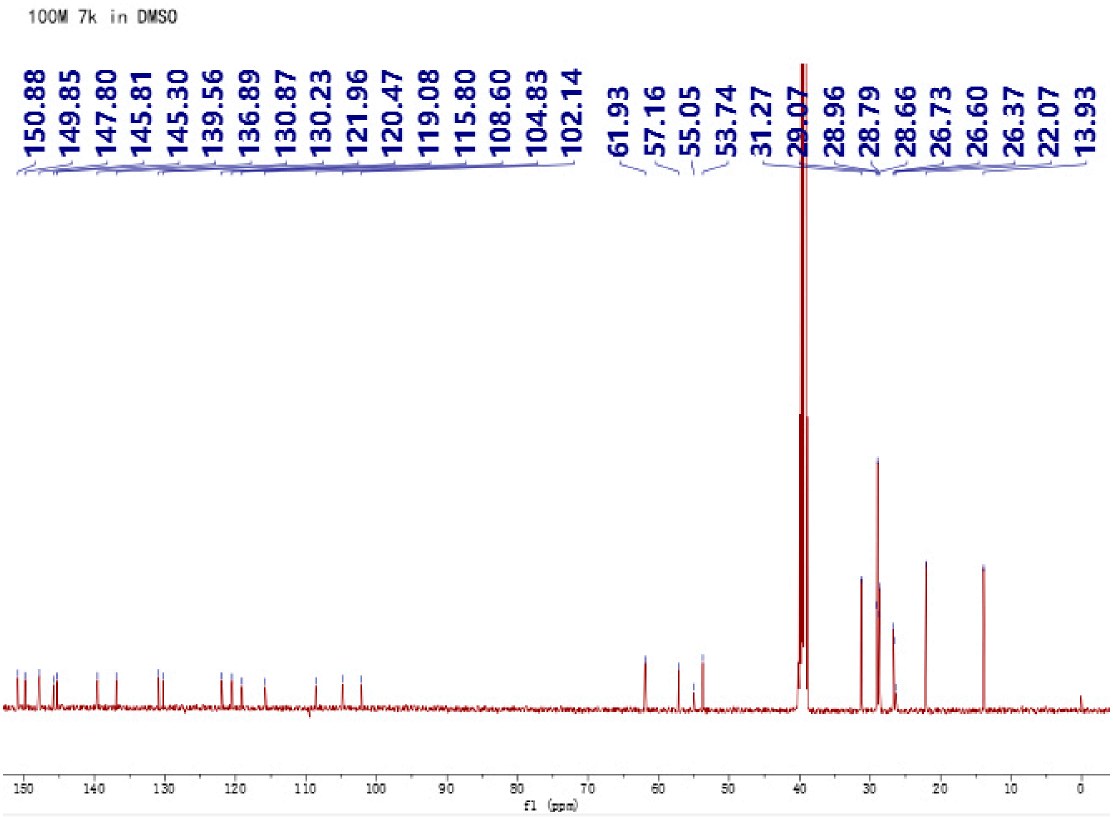

Figure S77. ^{13}C-NMR spectrum of compound **7k**

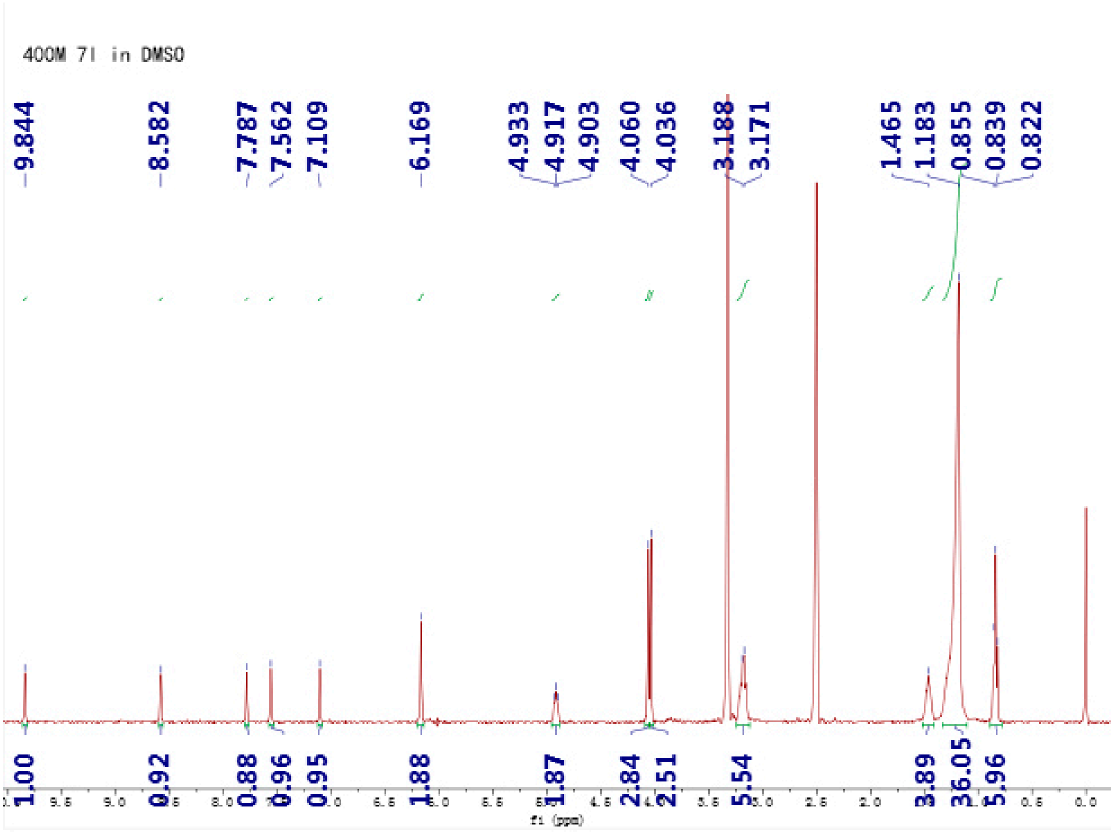

Figure S78. ^{1}H-NMR spectrum of compound **7l**

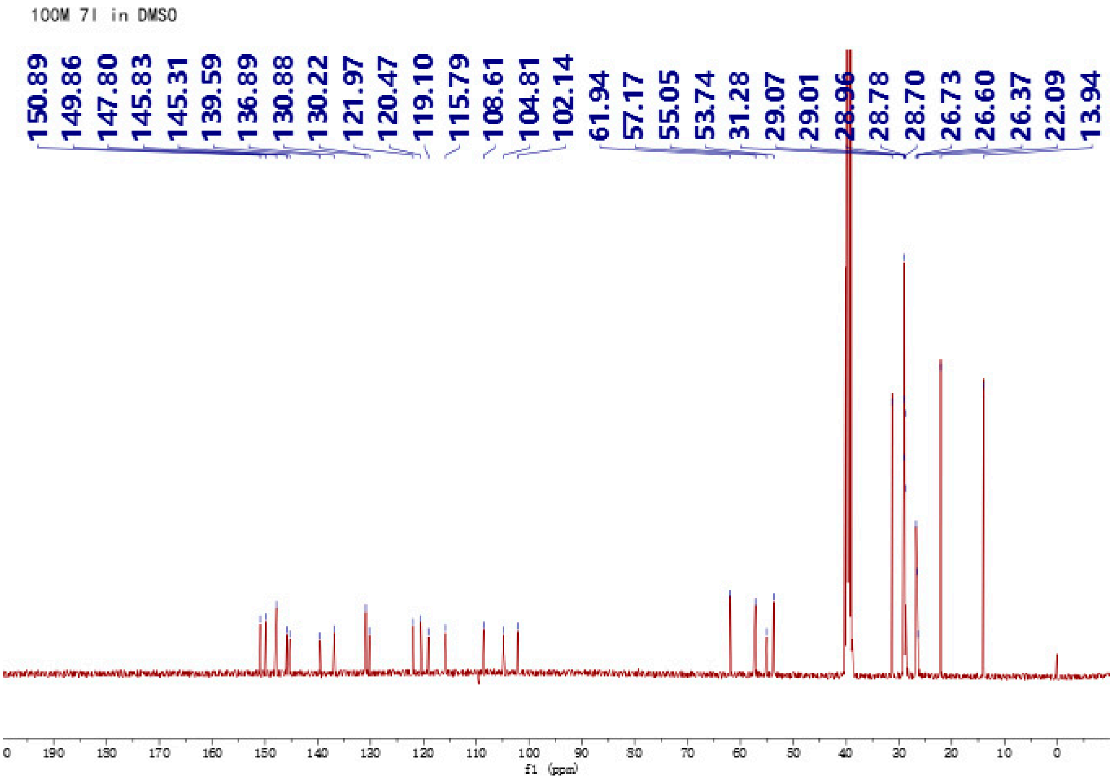

Figure S79. ^{13}C-NMR spectrum of compound **7l**

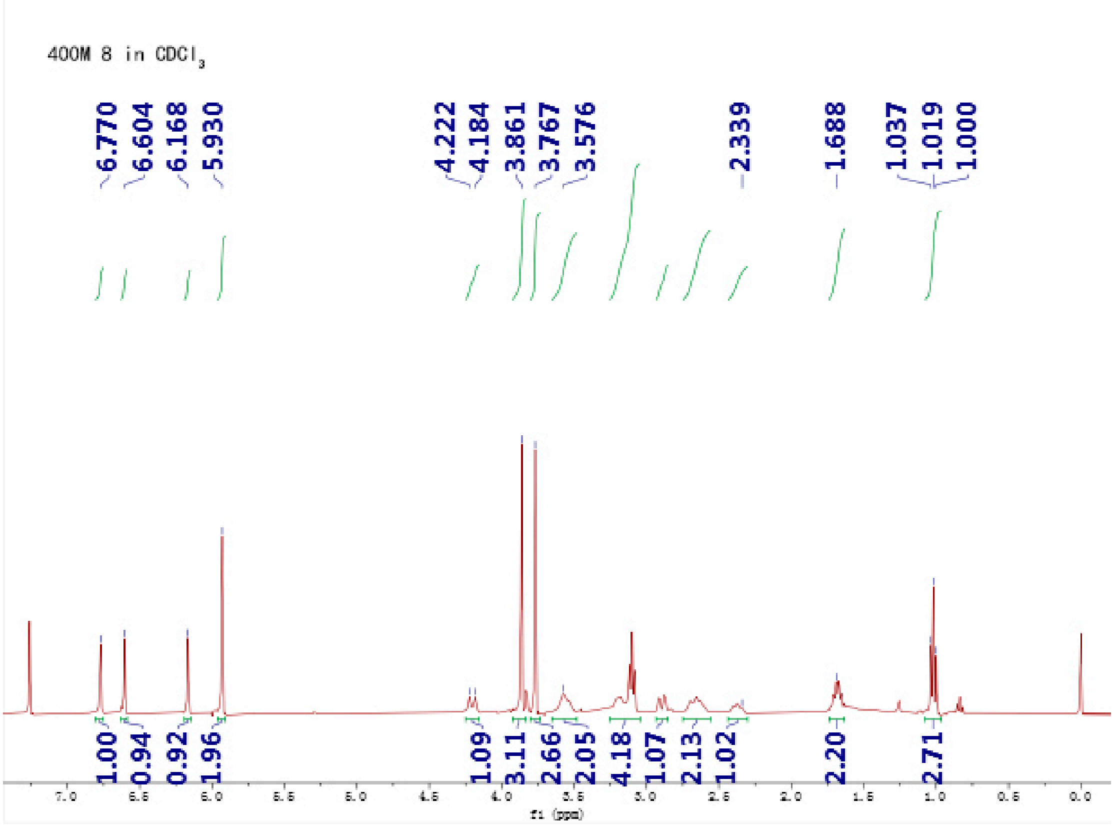

Figure S80. ^{1}H-NMR spectrum of compound **8**

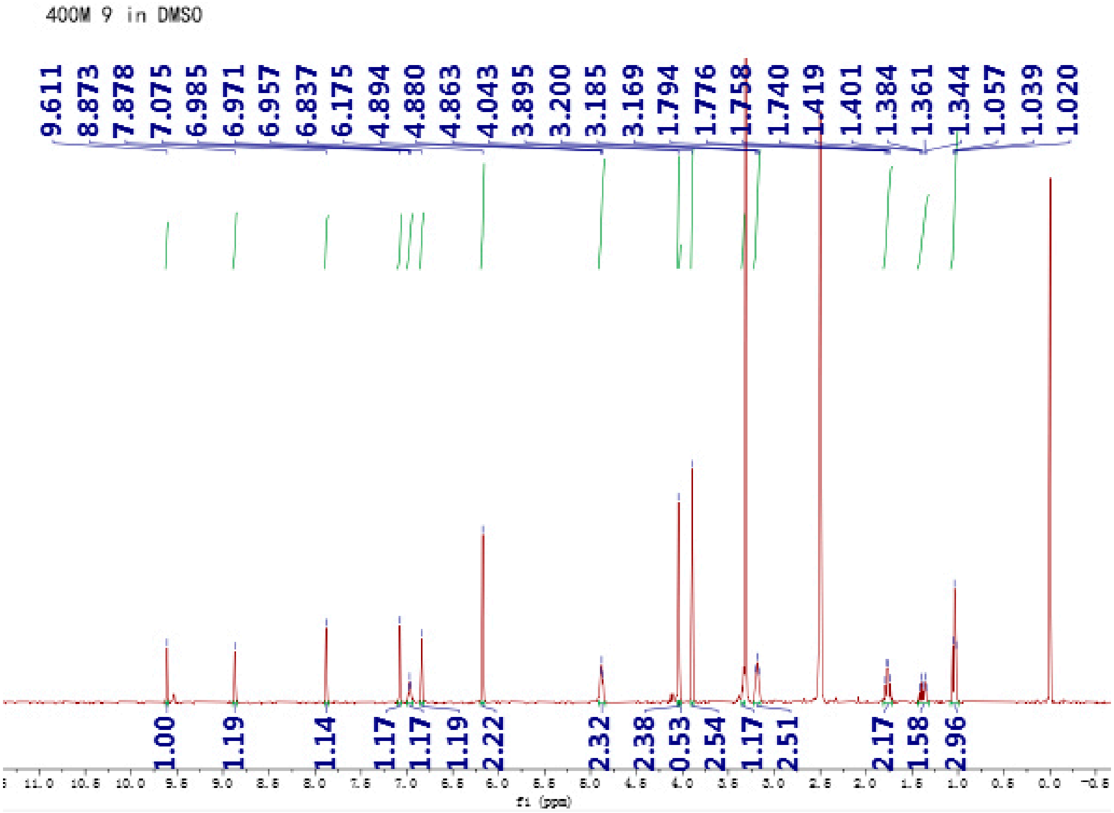

Figure S81. ^{1}H-NMR spectrum of compound **9**
